# Mg^2+^-Ion Dependence Revealed for a BAHD 13-*O*-β-Aminoacyltransferase from *Taxus* Plants

**DOI:** 10.1021/jacsau.4c00577

**Published:** 2024-09-30

**Authors:** Aimen Al-Hilfi, Zhen Li, Kenneth M. Merz, Kevin D. Walker

**Affiliations:** † Department of Chemistry, Michigan State University, East Lansing, Michigan 48824, United States; ‡ Department of Biochemistry and Molecular Biology, Michigan State University, East Lansing, Michigan 48824, United States

**Keywords:** new-generation taxanes, BAHD biocatalysis, molecular dynamics, Mg^2+^-dependence, paclitaxel

## Abstract

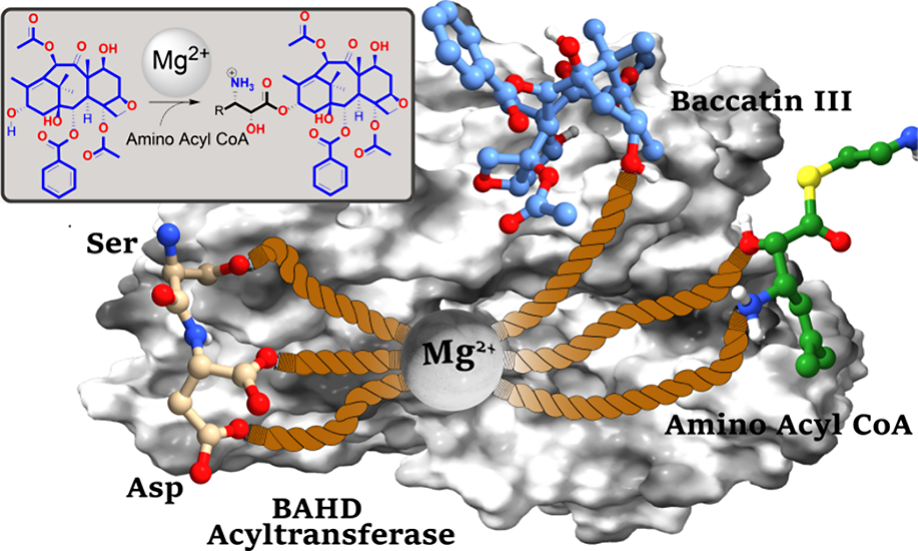

A *Taxus* baccatin III:3-amino-3-phenylpropanoyltransferase (BAPT, Accession: AY082804) in clade 6 of the BAHD family catalyzed a Mg^2+^-dependent transfer of isoserines from their corresponding CoA thioesters. An advanced taxane baccatin III on the paclitaxel biosynthetic pathway in *Taxus* plants was incubated BAPT and phenylisoserine CoA or isobutenylisoserinyl CoA with and without MgCl_2_. BAPT biocatalytically converted baccatin III to its 13-*O*-phenylisoserinyl and 3-(1',1'-dimethylvinyl)isoserinyl analogs, an activity that abrogated when Mg^2+^ ions were omitted. Baccatin III analogs that are precursors to new generation taxanes were also assayed with BAPT, the Mg^2+^ cofactor, and 3-(1',1'-dimethylvinyl)isoserinyl CoA to make paclitaxel derivatives at *k*_cat_/*K*_M_ ranging between 27 and 234 s^–1^ M^–1^. Molecular dynamics simulations of the BAPT active site modeled on the crystal structure of a BAHD family member (PDB: 4G0B) suggest that Mg^2+^ causes BAPT to use an unconventional active site space compared to those of other BAHD catalysts, studied over the last 25 years, that use a conserved catalytic histidine residue that is glycine in BAPT. The simulated six-membered Mg^2+^–coordination complex includes an interaction that disrupts an intramolecular hydrogen bond between the C13-hydroxyl and the carbonyl oxygen of the C4-acetate of baccatin III. A simulation snapshot captured an active site conformation showing the liberated C13-hydroxyl of baccatin III poised for acylation by BAPT through a potential substrate-assisted mechanism.

## Introduction

**Scheme 1:**
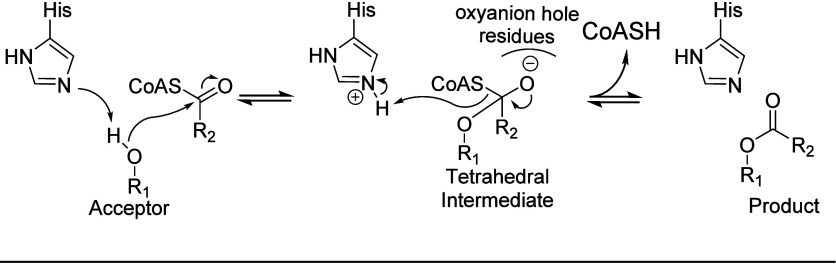
Proposed Catalytic Mechanism of BAHD Catalysis Generally Employs a His Residue as a General Base that Helps Deprotonate a Nucleophilic Hydroxy (or Amino) Group of the Acceptor Substrate as it Attacks the Carbonyl Group of the Acyl CoA The concerted proton transfer and nucleophilic attack leads to a tetrahedral oxyanion intermediate that collapses and displaces CoA–SH, which receives a proton from His to conclude the reaction cycle.

**Figure 1:**
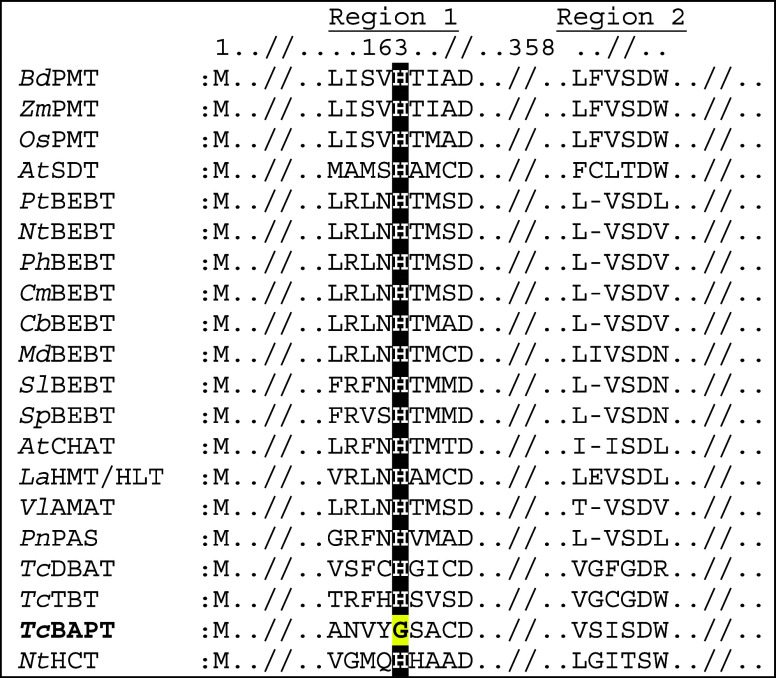
Partial sequence alignment of representative plant BAHD homologues from Clades 5 and 6. (Top Row) Residue numbers for *Tc***BAPT** (aka **BAPT** in this study) and regions containing *Tc***BAPT** active site residues are highlighted yellow. Enzyme abbreviations are below from these organisms (Accession numbers are in parentheses): *Bd***PMT**: *Brachypodium distachyon* (HG421450.1), *Zm***PMT**: *Zea mays* (BT042717.1), *Os***PMT**: *Oryza sativa* (XM015765814.1), *At***SDT**: *Arabidopsis thaliana* (NM_127915.2), *Pt***BEBT**: *Populus trichocarpa* (KP228019.1), *Nt***BEBT**: *Nicotiana tabacum* (AF500202), *Ph***BEBT**: *Petunia x hybrida* (AY611496), *Cm***BEBT**: *Cucumis melo* (AY859053), *Cb***BEBT**: *Clarkia breweri* (AF500200), *Md***BEBT**: *Malus domestica* (AY707098), *Sl***BEBT**: *Solanum lycopersicum*, *Sp***BEBT**: *Solanum pennelli* (KM975322 and KM975321.1), *At***CHAT**: *A. thaliana* (AF500201.1), *La***HMT**/**HLT**: *Lupinus albus* (AB181292.1), *Vl***AMAT**: *Vitis labrusca* (AY705388.1), *Pn***PAS**: *Piper nigrum* (MW354957), *Tc***DBAT**: *Taxus cuspidata* (AF193765), *Tc***TBT** (aka **DBBT**): *Taxus cuspidata* (AF297618), *Tc***BAPT**: *Taxus cuspidata* (AY082804), *Nt***HCT**: *N. tabacum* (Q8GSM7.1). Enzyme function: PMT: *p*-coumaroyl-CoA/monolignol transferase; SDT: spermidine disinapoyl acyltransferase from *Arabidopsis thaliana*; BEBT: benzoyl-CoA:benzyl alcohol *O*-benzoyltransferase; CHAT: acetyl coenzyme A: *Z*-3-hexen-1-ol acetyl transferase; HMT/HLT: 13-hydroxymultiflorine/hydroxylupanine *O*-tigloyltransferase; AMAT: methanol *O*-anthraniloyltransferase; PAS: piperoyl-CoA:piperidine piperoyl transferase; DBAT: 10-*O*-deacetylbaccatin III: acetyltransferase, TBT: taxane: 2-*O*-benzoyltransferase, BAPT: baccatin III: phenylpropanoyltransferase.; HCT: hydroxycinnamoyl-coenzyme A shikimate/quinate hydroxycinnamoyltransferase.

BAHD acyltransferases play crucial roles in modifying the characteristics of metabolites in plants and fungi by altering polarity, volatility, solubility, chemical stability, and biological activity.^1,2^ BAHD gene sequences have more recently been found in animals and insects (such as in *Saguinus oedipus* (accession: JASSZA010000010.1); *Bemisia tabaci* (accession: XP_018906383.1),^3^ demonstrating that the ubiquity of BAHD activity expands beyond plants. The BAHD acronym is derived from the names of the first four biochemically characterized enzymes of this superfamily (BEAT, AHCT, HCBT, and DAT), identified nearly 25 years ago.^2^ BAHD enzymes use acyl CoA donor substrates to acylate the amino or hydroxy groups of natural products, diversifying them to their esters and amides analogs.^2^ BAHD members typically share conserved HXXXD and DFGWG amino acid motifs (Figure 1), where the catalytic histidine functions as a general base involved in proton transfer (Scheme 1); the aspartate of the HXXXD sequence is essential structurally.^4^ The DFGW(or F)G(or K/A) sequence forms a portion of the acyl CoA access channel.^4,5^

The BAHD acyltransferases have been subdivided into clades, where clade 1 enzymes are involved primarily in the acylation of flavonoids, phenolic glucosides, and anthocyanins. Clade 2 catalysts elongate epicuticular waxes in *Arabidopsis thaliana* and *Zea mays*,^6^ and clade 3 contains a series of alcohol acyltransferases for making volatile lipids.^7^ Clade 3 members also modify alkaloids^8−10^ and *O*-acylate sugars with C2–C12 short-chain alkyl/aroyl groups in glandular trichomes.^11,12^ Clade 4 contains members such as the agmatine coumaroyltransferases (ACTs) found in barley and wheat^13,14^ and putrescine hydroxycinnamoyltransferases (PHTs) found in tobacco, rice, and tomato.^15−18^ Clade 5 mainly contains hydroxycinnamoyl-CoA:shikimate acid hydroxycinnamoyl transferases (HSTs) and hydroxycinnamoyl-CoA:quinate acid hydroxycinnamoyl transferase (HQTs) that use phenolic CoA thioesters as donors to catalyze acylation reactions with shikimic acid and quinic acid.^19^ Clade 6 contains acyltransferases that acylate medium-chain alkyl/aroyl alcohols with alkyl/aroyl groups donated by the cognate CoA thioesters. This clade contains a subgroup of *Taxus* acyltransferases involved in paclitaxel biosynthesis and contains similar conserved catalytic domains as seen among the entire BAHD family.^20−24^ However, the *Taxus* baccatin III:3-amino-3-phenylpropanoyltransferase (BAPT) sequence has a unique natural His → Gly mutation in the catalytic HXXXD motif (Figure 1).^24^ Based on the pervasive catalytic mechanism of BAHD enzymes, the natural mutation of the catalytic His → Gly in BAPT should abrogate activity. An earlier study proposed that the BAPT mechanism recruits substrate-assisted catalysis using the amino group of the β-amino-3-phenylpropanoyl CoA substrate to engage in proton transfer processes instead of the missing His residue to transfer the phenylpropanoyl group to the C13 oxygen the baccatin III acceptor.^24^

In the current study, we discovered that Mg^2+^, a biologically important cation in plant systems where the BAHD enzymes reside, stimulated BAPT activity. This discovery changes the optics of the ubiquitous BAHD mechanism that does not typically employ a metal cofactor. Accelerated molecular dynamics (MD) simulations suggest that the divalent metal cofactor liberates the C13-hydroxyl from its intramolecular H-bond tether within the baccatin III substrate, interacts with the amino hydroxy CoA cosubstrate to promote a substrate-assisted-catalysis mechanism, and reorganizes the active site residues. Here, we show, as proof-of-principle, the regioselective semibiocatalytic acylation of the C13-hydroxyl of baccatin III scaffolds (without silyl ether protection) by BAPT catalysis when Mg^2+^ is present. The kinetic parameters of the BAPT-catalyzed acylation for various *m*-(substituted)benzoyl acceptor cosubstrates and anisoserinyl CoA substrate variant were measured. This biocatalytic approach provides access to precursors of new-generation taxanes that bypass several steps in the decades-old procedures used to make these advanced taxane analogs.^25^

## Materials and Methods

### Materials

3-Methylbut-2-enal (97%), *p*-anisidine (99%), acetoxy acetyl chloride (97%), *tert*-butanol (≥99%), methanol (>99.5%), hexane (>99.5%), ethyl acetate (>99.5%), diisopropyl ether (≥98.5%), and immobilized CAL-B (lipase B from *Candida antarctica*) on the acrylic resin (L4777) were purchased from Sigma-Aldrich (St. Louis, MO). Ceric ammonium nitrate (99%) was purchased from Fisher Scientific Company (Fair Lawn, NJ). Coenzyme A (95%) was obtained from AmBeed (Arlington Hts, IL). Nickel-affinity chromatography resin (HisPur Ni-NTA Resin) was purchased from Thermo Fisher Scientific (Waltham, MA). ATP, DTT, isopropyl β-d-1-thiogalactopyranoside (IPTG), kanamycin, and phenylmethylsulfonyl fluoride (PMSF) were purchased from Gold Biotechnology (Olivette, MO). Baccatin III (>98%) and 10-*O*-deacetylbaccatin III (>98%) were purchased from Natland International Corporation (Research Triangle Park, NC). Triethylamine (100%) was obtained from J. T. Baker (Center Valley, PA). C18 silica gel resin (carbon 23%, 40–63 μm) was purchased from Silicycle, Quebec City, Quebec, Canada. The following compounds (Table 1) were biocatalyzed in earlier studies and were among the laboratory inventory.

**Table 1:**
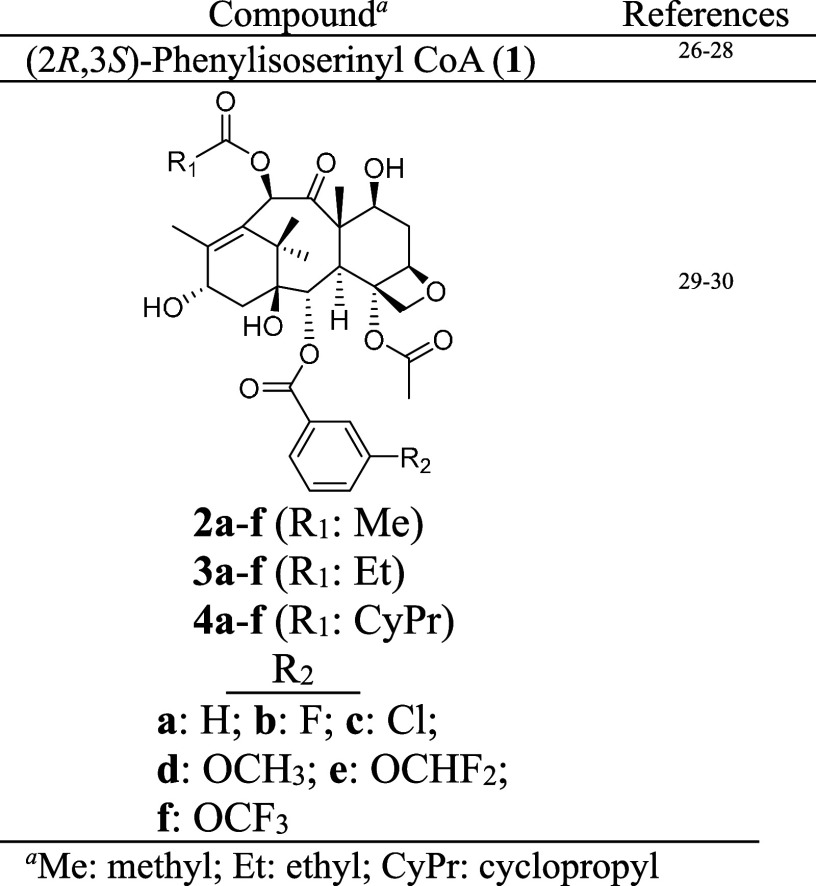
Compounds from Laboratory Inventory

a Me: methyl; Et: ethyl; CyPr: cyclopropyl.

### Synthesis of *N*-(*p*-Methoxyphenyl)-3-acetoxy-4-(2-methyl-1-propen-1-yl)azetidin-2-one *cis*-Racemate (**5**)

**Scheme 2:**
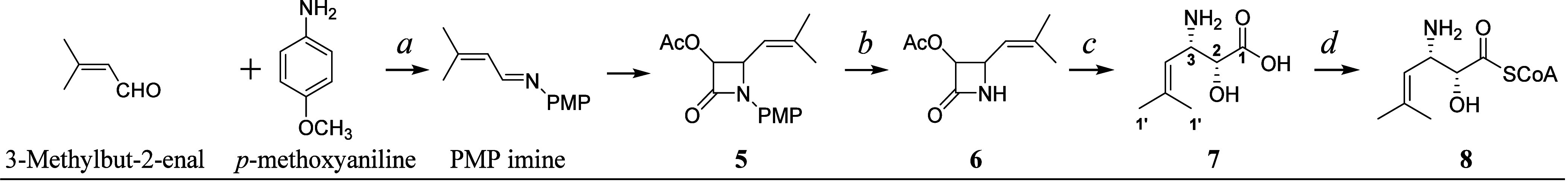
Summary of the Synthesis of (2*R*,3*S*)-3-(1′,1′-Dimethylvinyl)isoserine and Converting It Biocatalytically to Its CoA Thioester Using PheAT Reagent and conditions: (*a*) acetoxyacetyl chloride, TEA, CH_2_Cl_2_, 0 °C; (*b*) CAN, CH_3_CN, H_2_O, 0 °C-r.t.; (*c*) immobilized CAL-B, H_2_O, *i*Pr_2_O, r.t.; (*d*) PheAT, CoA, ATP, MgCl_2_·(6H_2_O).

The following methods to convert **5** to **7** (Scheme 2) are based on previous procedures.^31,32^ 3-Methylbut-2-enal (0.4 g, 4.8 mmol, 1 equiv) and *p*-methoxyaniline (1.48 g, 12 mmol, 2.5 equiv) were added to a 25 mL round-bottomed flask and dissolved in 15 mL dichloromethane. Oven-dried molecular sieves (∼1.5 g) were added to remove water formed during the reaction. The reaction was stirred at room temperature for 16 h. The molecular sieves were removed by filtration, and the filtrate was transferred to a clean, oven-dried round-bottomed flask, which was then sealed with a rubber septum. Triethylamine was added to this crude imine mixture (2.1 mL, 14.4 mmol, 3 equiv) and stirred at 0 °C. Acetoxyacetyl chloride (1 mL, 9.3 mmol, 2 equiv) was separately dissolved in dichloromethane, and the solution was added slowly to the reaction mixture. The reaction was stirred at 0 °C for an additional 5 min, then warmed to room temperature and stirred for 5 h. The solution was washed successively with 5% (w/v) NaHCO_3_ (15 mL), 5% v/v HCl (15 mL), and water (3 × 15 mL). The organic fraction was dried (MgSO_4_) and concentrated under a vacuum. The crude mixture was purified by silica gel column chromatography (1:4 EtOAc/hexane, v/v) to yield a pure product (**5**) as determined by NMR (Figures S1 and S2 of the Supporting Information).

### Synthesis of 3-Acetoxy-4-(2-methyl-1-propen-1-yl)azetidine-2-one *cis*-Racemate (**6**)^31^

*N*-(*p*-Methoxy phenyl)-3-acetoxy-4-(2-methyl-1-propen-1-yl)azetidine-2-one (**5**) (0.25 g, 0.86 mmol, 1 equiv) were added to a 50 mL round-bottomed flask, dissolved in CH_3_CN (14 mL), and the solution was cooled to 0 °C for 10 min. Ceric ammonium nitrate [(NH_4_)_2_Ce(NO_3_)_6_)] (2.58 g, 1.41 mmol, 3 equiv), dissolved in water (16 mL), was added dropwise to the solution. The mixture was stirred at 0 °C until the starting material disappeared by TLC analysis and then diluted with water (20 mL). The mixture was extracted with EtOAc (3 × 20 mL). The organic layer was washed with 5% (w/v) NaHCO_3_ (15 mL), and the aqueous extracts were washed with EtOAc (20 mL). The combined organic extracts were washed sequentially with 10% (w/v) Na_2_SO_3_ (15 mL), 5% (w/v) NaHCO_3_ (15 mL), and brine (15 mL). The combined extracts were dried over MgSO_4_ and concentrated under a vacuum. The mixture was purified by silica gel column chromatography (1:3 EtOAc/hexane, v/v) to yield a pure product (**6**) as determined by NMR (Figures S3 and S4 of the Supporting Information).

### Synthesis of (2*R*,3*S*)-3-(1′,1′-Dimethylvinyl)isoserine (**7**)

3-Acetoxy-4-(2-methyl-1-propen-1-yl)azetidin-2-one racemate (**6**) (100 mg, 0.55 mmol) was added to a 50 mL round-bottomed flask and dissolved in diisopropyl ether (30 mL). Immobilized CAL-B (1.5 g, 50 mg/mL) and H_2_O (2 mL) were added, and the mixture was stirred at 60 °C for 72 h. The reaction precipitate was filtered to remove the immobilized enzyme, and the filtrate was washed with water (3 × 15 mL). The water layers were collected and removed under a stream of nitrogen gas. The residue was dissolved in acetonitrile (100 μL), and an aliquot was analyzed by LC/ESI-MS to assess the conversion to the (2*R*,3*S*)-3-amino-2-hydroxy-5-methylhex-4-enoic acid (referred to herein as (2*R*,3*S*)-3-(1′,1′-dimethylvinyl)isoserine, see Scheme 2 for numbering) (**7**) (Figure S5 of the Supporting Information). The crude residue was loaded onto a C18 reverse-phase silica-gel column (1.2 mm × 32 cm) and eluted with 10% acetonitrile in water. The fractions containing the product were collected and lyophilized to yield a pure product (**7**) as determined by NMR (Figures S6 and S7 of the Supporting Information). These procedures were repeated as needed to obtain sufficient isoserine material for downstream enzyme kinetic analyses and scale-up procedures.

### Expression and Purification of the PheAT Enzyme

A glycerol stock of *E. coli* BL21 (DE3) transformed with the plasmid *KDW-pET28a-phe-at* encoding the *pheat* gene for expression of the PheAT enzyme^27^ was used to inoculate 250 mL of Lysogeny Broth (LB) containing kanamycin (50 μg/mL). The seed culture was incubated at 37 °C overnight, and the inoculum culture (25 mL) was added to LB media (10 × 1 L) containing kanamycin (50 μg/mL). The cells were incubated at 37 °C until OD_600_ = 0.6, then IPTG (250 μM final concentration) was added, and the strains were incubated at 16 °C. After 16 h, the cultures were centrifuged (2100*g*) for 1 h at 4 °C to pellet the cells. The cells were resuspended in 100 mL of Lysis Buffer (50 mM sodium phosphate (pH 8.0), 300 mM NaCl, 10 mM imidazole, and 5% (v/v) glycerol), and lysed by sonication (Misonix Sonicator; Danbury, CT): 10 s on, 20 s rest for 30 cycles) on ice. The cell debris was removed by centrifugation (1500*g*) for 45 min at 4 °C followed by high-speed centrifugation (25,000*g*) for 90 min at 2 °C to remove light membrane debris. The supernatant was loaded onto a column containing 3 mL of nickel-nitrilotriacetic acid (Ni-NTA) resin and eluted by gravity flow. The column was washed with 50 mL of Wash Buffer 1 (300 mM NaCl, 50 mM sodium phosphate (pH 8.0), 10 mM imidazole, and 5% (v/v) glycerol) and 20 mL of Wash Buffer 2 (300 mM NaCl, 50 mM sodium phosphate (pH 8.0), 50 mM imidazole, and 5% (v/v) glycerol). Protein was eluted with Elution Buffer (300 mM NaCl, 50 mM sodium phosphate (pH 8.0), 250 mM imidazole, and 5% (v/v) glycerol). Each eluted fraction was analyzed by SDS-PAGE to establish the presence and purity of the protein. Fractions containing enzymes with a molecular weight consistent with PheAT (∼70 kDa) were combined and loaded onto a size-selective centrifugal filtration unit (30,000 NMWL, Millipore Sigma, Burlington, MA). The protein solution was concentrated to 1 mL and diluted several cycles until the imidazole and salt concentrations were <1 μM. The quantity of PheAT (12 mg) was measured using a Nanodrop spectrophotometer, and the purity was assessed by SDS-PAGE with Coomassie Blue staining (Figure S8 of the Supporting Information). These procedures were repeated as needed to obtain sufficient catalyst for downstream kinetic analyses and scale-up procedures.

### Screening PheAT Activity with (2*R*,3*S*)-3-(1′,1′-dimethylvinyl)isoserine (**7**) and CoA

A solution of **7** (1 mM) in 50 mM NaH_2_PO_4_/Na_2_HPO_4_ buffer (pH 8) (1 mL) was incubated with purified PheAT (25 μg/mL). CoA (1 mM), ATP (1 mM), and MgCl_2_·(6H_2_O) (5 mg) were added to the solution, and the assay mixture was incubated at 31 °C on a rocking shaker for 4 h. The reaction was stopped by adding 8.8% formic acid to pH 4 to precipitate PheAT. The reaction was centrifuged at 5000*g* for 10 min to pellet the precipitate. The supernatant was collected, and the pellet was washed with water (pH 4, adjusted with formic acid) and centrifuged. Supernatants were combined and filtered through a Millipore Amicon Ultra 30 kDa concentrator to remove trace protein. The flow-through was collected, flash-frozen in liquid nitrogen, and lyophilized. The residue was dissolved in acetonitrile (100 μL), and an aliquot was analyzed by LC/ESI-MS to assess the relative conversion to the (2*R*,3*S*)-3-(1′,1′-dimethylvinyl)isoserinyl CoA (**8**) (Figure S9 of the Supporting Information).

### Kinetics Evaluation of PheAT Catalysis with **7**

The steady-state conditions for protein concentration and time were established for PheAT and **7** separately incubated at low (0.05 mM) and high (1 mM) concentrations in 10 mL of assay buffer [50 mM NaH_2_PO_4_/Na_2_HPO_4_ buffer (pH 8)] containing PheAT (150 μg/mL) and CoA (1 mM), ATP (1 mM), and MgCl_2_·(6H_2_O) (100 mg) at 31 °C on a rocking shaker. Aliquots (1 mL) were removed, and the biocatalysis reaction was stopped by adding 8.8% formic acid at 10, 15, 30, and 45 min and 1, 2, 3, 5, 7, and 10 h. (2*R*,3*S*)-Phenylisoserinyl CoA (0.15 mM) was added as the internal standard to correct the loss of analyte during the isolation of the product. Each sample was flash-frozen in liquid nitrogen and lyophilized. The resultant residue from each assay was separately resuspended in acetonitrile (100 μL) and quantified by LC/ESI-MS/MS. A stop time was established for the steady-state time range, and PheAT (150 μg/mL) and CoA (1 mM), ATP (1 mM), and MgCl_2_·(6H_2_O) (100 mg) were incubated with varying concentrations of **7** (0.05–1 mM), respectively, in triplicate assays at 31 °C on a rocking shaker for 3 h. As described above, assay products were extracted from the reaction mixture and quantified by LC/ESI-MS/MS. The kinetic parameters (*K*_M_ and *k*_cat_) were calculated by nonlinear regression with Origin Pro 9.0 software (Northampton, MA) using the Michaelis–Menten equation: *v*_0_ = *k*_cat_[*E*_0_][*S*]/(*K*_M_ + [*S*]) (Figure S10 of the Supporting Information).

### Production Scale-Up and Purification of (2*R*,3*S*)-3-(1′,1′-Dimethylvinyl)isoserinyl CoA (**8**)

A large-scale preparative PheAT enzymatic assay was carried out to complete the overall semisynthesis of **8** (0.31 mmol, 50 mg) by adding a concentrated solution of PheAT (36 mg/mL) in 50 mM NaH_2_PO_4_/Na_2_HPO_4_ (pH 8) (10 test tubes × 2 mL assay in each tube), (2*R*,3*S*)-3-(1′,1′-dimethylvinyl)isoserinyl (**8**), MgCl_2_·(6H_2_O) (20 mg) to the buffer. Separately, ATP (0.31 mmol) and CoA (0.31 mmol) were dissolved in 1 mL each of 50 mM phosphate buffer (pH 8). The ATP and CoA solutions were then added to the PheAT solution, and the mixture was incubated for 14 h at 31 °C on a rocking shaker. Additional ATP and CoA were added, and the reaction was incubated for 7 h at 31 °C. This reaction sequence was repeated as needed to obtain adequate acyl CoA for kinetic analyses and the scale-up assays with the downstream acyltransferase enzyme. The reaction was stopped by adding 8.8% formic acid to pH 4 to precipitate PheAT. The precipitated reaction was centrifuged at 5000*g* for 10 min. The supernatant was collected, and the pellet was washed with water (pH 4, adjusted with formic acid) and centrifuged. Supernatants were combined and filtered through a Millipore Amicon Ultra 30 kDa concentrator to remove trace protein. The flow-through was collected and lyophilized, and the crude product was dissolved in ultrapure water (2 mL, pH 4) for preparative HPLC purification.

An aliquot (100 μL) of the crude (2*R*,3*S*)-3-(1′,1′-dimethylvinyl)isoserinyl CoA (**8**) was loaded onto a preparative C18 column (Atlantis C18 OBD, 5 μm, 19 mm × 150 mm). The column was eluted at 5 mL/min with 5% solvent B (100% acetonitrile) and 95% solvent A (0.1% trifluoroacetic acid in water) with a 5 min hold, a linear gradient to 30% solvent B over 15 min, then increased to 100% solvent B over 4 min, and finally lowered to 5% solvent B over 5 min. Peak fractions were collected, flash-frozen, and lyophilized to yield a pure product as determined by NMR. A portion of the purified residue was dissolved in acetonitrile (100 μL), and an aliquot was analyzed by LC-MS/MS for fragmentation analysis and monoisotopic mass calculation (Figures S11–S13 of the Supporting Information).

### Expression and Purification of the BAPT Enzyme

A glycerol stock of *E. coli* BL21(DE3) engineered to express the NterBAPT,^28^ enzyme (referred to herein as BAPT) from the *bapt*-pET28a-*bapt* plasmid containing the *bapt* gene was used to inoculate Lysogeny Broth (LB) (400 mL) containing kanamycin (50 μg/mL) and incubated at 37 °C overnight. This inoculum culture (50 mL) was added to fresh LB media (8 × 1 L) containing kanamycin (50 μg/mL). The cells were incubated at 37 °C until OD_600_ ≈ 0.6, IPTG (250 μM final concentration) was added, and the strains were incubated at 16 °C for 16 h. The cultures were centrifuged (2100*g*) for 1 h at 4 °C to pellet the cells. The cells were resuspended in 100 mL of lysis buffer (50 mM sodium phosphate (pH 8.0), 300 mM NaCl, 10 mM imidazole, and 5% (v/v) glycerol) and lysed by sonication (Misonix Sonicator (Danbury, CT): 10 s on, 20 s rest for 30 cycles) on ice. The cell debris was removed by centrifugation (1500*g*) for 45 min at 4 °C, followed by high-speed centrifugation (25,000*g*) for 90 min at 2 °C to remove light membrane debris. The supernatant was loaded onto a column containing nickel-nitrilotriacetic acid (Ni-NTA) resin (3 mL) and eluted by gravity flow. The column was washed with 50 mL of Wash Buffer 1 (300 mM NaCl, 50 mM sodium phosphate (pH 8.0), 10 mM imidazole, and 5% (v/v) glycerol) and 20 mL of Wash 2 Buffer (300 mM NaCl, 50 mM sodium phosphate (pH 8.0), 50 mM imidazole, and 5% (v/v) glycerol). Protein was eluted with Elution Buffer (300 mM NaCl, 50 mM sodium phosphate (pH 8.0), 250 mM imidazole, and 5% (v/v) glycerol).

Fractions containing enzymes of a molecular weight consistent with that of BAPT (∼51 kDa) were combined and loaded onto a size-selective centrifugal filtration unit (30,000 NMWL, Millipore-Sigma, Burlington, MA). The protein solution was concentrated to 1 mL and diluted several cycles until the imidazole and salt concentrations were <1 μM. The quantity of BAPT (8 mg total) was measured using a NanoDrop spectrophotometer, and the purity of the enzyme was assessed by SDS-PAGE and Coomassie Blue staining (Figure S14 of the Supporting Information). These procedures were repeated as needed to obtain enough catalyst for downstream kinetic analyses and scale-up procedures.

### Screening BAPT Activity with (2*R*,3*S*)-Phenylisoserinyl CoA (**1**), (2*R*,3*S*)-3-(1′,1′-Dimethylvinyl)isoserinyl CoA (**8**), and Baccatin III (**2a**)

**Figure 2:**
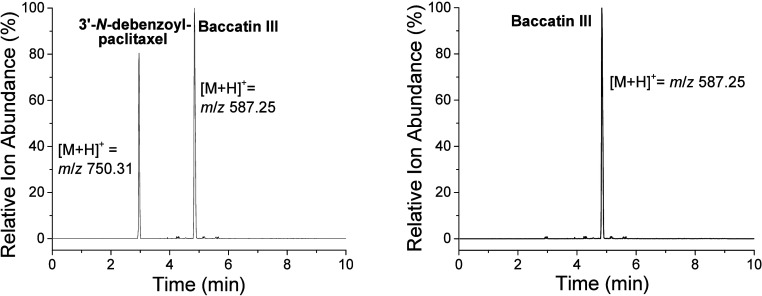
LC/ESI-MS in selected ion mode scanning for [M + H]^+^ ions for the putative biocatalysis product 3′-*N*-debenzoylpaclitaxel and baccatin III in assays containing BAPT, phenylisoserinyl CoA, and (A) MgCl_2_ or (B) no MgCl_2_.

**Figure 3:**
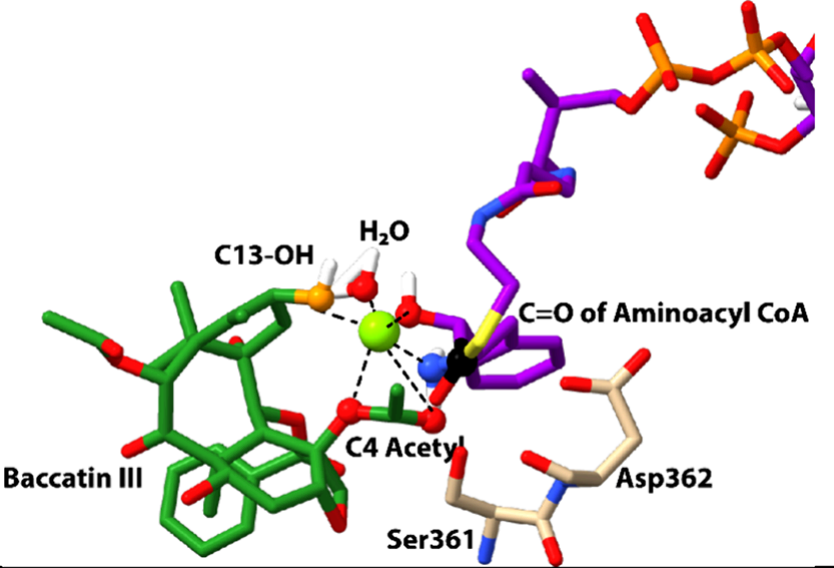
Structures of baccatin III (**2a**) (green sticks), phenylisoserinyl CoA (purple sticks, partial structure), and Mg^2+^ (green sphere) within the BAPT active site resulted from MD simulations. The atoms estimated to form a hexacoordinated complex with Mg^2+^ are drawn as spheres. Proximate residues Ser361 and Asp362 are shown for reference (see Figure 4). The donor carbonyl reaction center of the CoA thioester and the acceptor C13-hydroxyl of baccatin III are shown as black and orange balls, respectively.

A solution of baccatin III (**2a**) (1 mM), purified BAPT (25 μg/mL), biocatalyzed (2*R*,3*S*)-3-(1′,1′-dimethylvinyl)isoserinyl CoA (1 mM) (**8**) in 50 mM NaH_2_PO_4_/Na_2_HPO_4_ buffer (pH 7.4) (1 mL) were incubated separately without and with MgCl_2_·(6H_2_O) (1 mM). Semibiocatalyzed (2*R*,3*S*)-phenylisoserinyl CoA (**1**) (1 mM, from the laboratory chemical inventory, Table 1) was used in place of **8** in identical assays with BAPT (separately without and with MgCl_2_·(6H_2_O) (1 mM)) as a positive control.^26−28^ The assays were mixed at 31 °C on a rocking shaker for 4 h. The reaction was then stopped with EtOAc (3 × 1 mL). The EtOAc extracts were combined, and the solvent was removed under a stream of nitrogen gas. The residue was dissolved in acetonitrile (100 μL), and an aliquot was analyzed by LC/ESI-MS to assess ions consistent with 3′-*N*-debenzoylpaclitaxel (see Figure 2) and 3′-*N*-de(*tert*-butoxycarbonyl)-SB-T-1212 (**9a**) (see Figure 5).

### Kinetics Evaluation of BAPT Catalysis with (2*R*,3*S*)-3-(1′,1′-Dimethylvinyl)isoserinyl CoA and Baccatin III (and Its Analogs)

The steady-state conditions for protein concentration and time were established for BAPT and acyl CoA **8** separately incubated at low (0.05 mM) and high (1 mM) concentrations in 10 mL of assay buffer [50 mM NaH_2_PO_4_/Na_2_HPO_4_ buffer (pH 8)] containing BAPT (250 μg/mL), taxane analogs (1 mM), and MgCl_2_·(6H_2_O) (40 mg) at 31 °C on a rocking shaker. At each time point, the reaction was stopped by adding EtOAc (500 μL), and docetaxel (0.15 mM) was added as the internal standard to correct the loss of analyte during the isolation of the product. Each sample was extracted with EtOAc (4 × 1 mL), the organic fractions were combined, and the solvent was removed under a stream of nitrogen. The resultant residue from each assay was separately resuspended in acetonitrile (100 μL) and quantified by LC/ESI-MS/MS. A stop time was established for the steady-state time range, and BAPT (250 μg/mL), taxane analogs (1 mM), and MgCl_2_·(6H_2_O) (40 mg) were incubated with varying concentrations of (2*R*,3*S*)-3-(1′,1′-dimethylvinyl)isoserinyl CoA (**8**) (0.05–1 mM), respectively, in triplicate assays at 31 °C on a rocking shaker for 2 h. As described above, assay products were extracted from the reaction mixture and quantified by LC/ESI-MS/MS. The kinetic parameters (*K*_M_ and *k*_cat_) were calculated by nonlinear regression with Origin Pro 9.0 software (Northampton, MA) using the Michaelis–Menten equation: *v*_0_ = *k*_cat_[*E*_0_][*S*]/(*K*_M_ + [*S*]) (Figures S15–S17 of the Supporting Information).

### Production Scale-Up of 3′-*N*-De(*tert*-butoxycarbonyl)-SB-T-121(**2**/**3**/**4**) Analogs

A concentrated solution of BAPT (250 μg/mL, ∼8 mg total) was incubated in 50 mM NaH_2_PO_4_/Na_2_HPO_4_ assay buffer (pH 7.4) (15 test tubes × 2 mL assay in each tube) containing (1 mM) of taxane analogs (**2**, **3**, or **4** (**a**–**f**)) and (2*R*,3*S*)-3-(1′,1′-dimethylvinyl)isoserinyl CoA (1 mM), and MgCl_2_·(6H_2_O) (80 mg) at 31 °C on a rocking shaker for 4 h. The reaction was then stopped with ethyl acetate (2 × 3 mL) to extract the taxane substrates from the assay. The EtOAc extracts were combined, and the solvent was removed under a stream of nitrogen.

Alternatively, PheAT and BAPT activities were coupled as described previously,^28^ using the scale-up parameters described in this document and run serially to acquire enough acyl CoA substrate for the downstream BAPT-catalyzed reaction and enough product for NMR characterization. Typically, the 20 mL assays (10 assay tubes × 2 mL) contained MgCl_2_ (1 mM) ATP (1 mM), 3-(1′,1′-dimethyl)vinylisoserine (1 mM), and baccatin III (or its analog) (1 mM) were prepared on ice and incubated at 31 °C for 5 min before PheAT (∼3 mg/mL) was added. After 1 h, BAPT (∼250 μg/mL) was added to each 2 mL assay tube and allowed to react for 14 h. Additional enzyme (PheAT at ∼500 μg/mL and BAPT at ∼50 μg/mL) was titrated into each assay tube and incubated for ∼7 h. The reaction was basified to pH 9 with a concentrated aqueous NaHCO_3_ solution and extracted with EtOAc (3 × 3 mL) to remove the taxanes from the assay. The EtOAc extracts were combined, and the solvent was removed under a stream of nitrogen. The residue was purified by basic-alumina gel column-chromatography (50/50 hexane:EtOAc) to yield a pure product, as determined by NMR (Figures S18–S53 of the Supporting Information). The purified residue was dissolved in acetonitrile (100 μL), and an aliquot was analyzed by LC-MS/MS for fragmentation analysis and monoisotopic mass calculation (Figures S54–S56 of the Supporting Information).

### Molecular Modeling Analysis

Structure optimizations on baccatin III and (2*R*,3*S*)-isoserinyl CoA were conducted using Gaussian 16 in a four-step pattern,^33^ starting from HF 3-21G* single point to HF 3-21G* optimization, then to B3LYP 3-21G*, and finally to B3LYP 6-31G*. MD simulations were performed using AMBER22.^34^ The system was prepared in three steps. First, the antechamber, prepin, and parmchk2 programs in the AmberTools23 package^35^ generated the charge and force constants. The 12-6-4 LJ-type nonbonded model was used to establish the Mg^2+^ parameters,^36^ and the system was dissolved in OPC water.^37^ Minimization was done in five stages, gradually removing restrictions from the protein backbone to the side chain. Each step yields 10,000 steps of the steepest descendent and 10,000 steps of conjugate gradient methods. A quick 9 ps *NPT* simulation was conducted to avoid the formation of bubbles during heating. Afterward, a 36 ns *NVT* heating was performed with the temperature increasing gradually from 0 to 300 K. Then another 20 ns simulation was performed to equilibrate the system in the *NPT* ensemble, and the last 2000 frames were used for distance analysis. The PME method and PBC were used for the simulations, and the Langevin algorithm with a 2.0 ps^–1^ friction frequency coefficient was used for maintaining the temperature.^38^ The Berendsen barostat method was used for pressure control with a relaxation time of 1.0 ps.^39^ The time step was 1.0 fs, with the SHAKE function constraining the hydrogen atom bonds.^40^

## Results and Discussion

### Establishing the Mg^2+^ Dependency of the BAHD Enzyme BAPT

BAPT is a member of the larger BAHD family of plant acyltransferases, which invariably use a catalytic histidine residue to facilitate the transfer of an acyl group from a CoA carrier to an acceptor molecule.^41^ A sequence comparison between BAPT and its BAHD homologues shows that BAPT has a glycine residue instead of histidine (cf. Figure 1). An earlier study posited that BAPT likely proceeded through substrate-assisted catalysis, where the side chain amino group of the isoserinyl CoA replaced the function of the missing histidine.^24^ In the current study, when BAPT was incubated with (2*R*,3*S*)-phenylisoserinyl CoA (**1**) and baccatin III (**2**). The putative product made biocatalytically was screened by LC/ESI-MS selected-ion monitoring for ion *m*/*z* 750.31 corresponding to [M + H]^+^ for 3′-*N*-debenzoylpaclitaxel (Figure 3).

Unexpectedly, the product, 3′-*N*-debenzoylpaclitaxel, was below the detection limit of the mass spectrometer. This result was contrary to those in the original study describing the first characterization of BAPT, where BAPT was assayed within a ∼500-μg milieu of crude protein isolated from the expression host *Escherichia coli*, 100 μM acyl CoA, and 70 μM [^3^H]baccatin III with sensitive radioactive detection.^24^ In the current study, BAPT was purified to near homogeneity, assayed at 25 μg/mL with 1 mM of phenylisoserinyl CoA and baccatin III, and thus projected to turnover baccatin III robustly to 3′-*N*-debenzoylpaclitaxel.

**Scheme 3:**
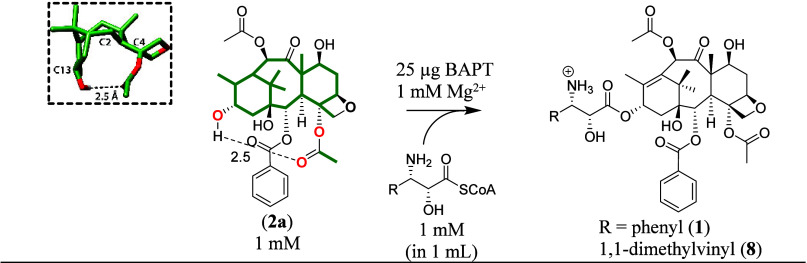
Biocatalysis Assay to Assess the Proposed Metal Ion Stimulation of the BAPT Reaction with Phenylisoserinyl CoA or 3-(1′,1′-dimethylvinyl)isoserinyl CoA The dotted line (representing 2.5 Å) shows the putative H-bond between the C13–OH and the C=O group of the C4-acetyl of baccatin III (**2a**). Inset: the endo configuration of the taxane A, B, and C rings of (**2a**) tethered by the intramolecular H-bond.

Perplexed by the lack of activity, our recent data on another BAHD *Taxus* acyltransferase *m*TBT (derived from *wt*-TBT, Accession: AF297618; “*m*” denotes mutations in the N-terminus of the enzyme to increase its soluble expression) suggested that an intramolecular hydrogen bond (H-bond) within the baccatin III substrate (**2**) potentially precluded BAPT activity.^42^ Based on the substrate specificity of the *Taxus m*TBT catalysis and MD simulations on the substrate/enzyme interactions, we saw how an intramolecular H-bond between the 13-hydroxyl and the 4-acetoxy of the taxane substrate affected *m*TBT activity (Scheme 3).^29^ Oxidizing the 13-hydroxyl group of the taxane substrate to a keto group stimulated *m*TBT activity by enabling the taxane to orient properly for acylation.^29^

BAPT and *m*TBT use structurally similar baccatin substrates, except BAPT acylates the C13-hydroxyl group of baccatin III, while *m*TBT acylates the C2-hydroxyl of 2-*O*-debenzoylbaccatin III. The propensity of the C13-hydroxyl to H-bond intramolecularly to the carbonyl oxygen of the C4 acetoxy of baccatin III potentially makes the C13-hydroxyl inaccessible for acylation by BAPT catalysis. We hypothesized that the intramolecular H-bond must be disrupted to stimulate BAPT catalysis to liberate the C13-OH of baccatin III for nucleophilic attack on the reactive carbonyl group of the acyl CoA donor.

Examples in the literature describe that natural biological systems use metal cations such as the monovalent alkali cations (Li^+^, Na^+^, K^+^) and the divalent alkaline earth metal cations (Mg^2+^ and Ca^2+^) to effect intra- and extracellular responses due to their different interactions with nitrogen or oxygen donor ligands.^43,44^ One study used molecular modeling to show how alkaline earth metal Mg^2+^ and Ca^2+^ ions affected the H-bond network of inositol 2-phosphate and consequently altered the ring conformations in a simulated protein environment.^45^ An orthogonal study showed that Mg^2+^, Ca^2+^, and Ba^2+^ could disrupt the intermolecular H-bonding between ferrocenemethanol and the carboxylate groups of 3-mercaptopropionic acid on a monolayer.^46,47^

These early studies describing the relationship between metal ions and hydrogen-bond interactions inspired us to explore a metal ion to stimulate BAPT catalysis by interrupting the putative H-bond between the C13-hydroxyl and the carbonyl of the C4-acetate of baccatin III analogs, suggested by MD simulations (Figure 2).^29^ The selection of Mg^2+^ as a putative BAPT cofactor was significantly driven by an earlier study in which BAPT was used in a sequential reaction assay with a Mg^2+^-dependent acyl CoA ligase whose activity was promoted by 3.5 mM Mg^2+^ cations.^28^ In the earlier coupled reaction assay containing BAPT and the acyl CoA ligase, Mg^2+^ likely not only facilitated the CoA ligase activity but may have unknowingly stimulated the BAPT activity. Therefore, purified BAPT was incubated with baccatin III, (2*R*,3*S*)-phenylisoserinyl CoA, and MgCl_2_·(6H_2_O) as a cofactor, buoyed by the compulsory need for Mg^2+^ in green plants (including *Taxus* sp.) for chlorophyll maintenance and enzyme activation, as examples.^48^ The BAPT biocatalysis reaction was screened by LC/ESI-MS/MS in selected-ion monitoring mode and identified an ion *m*/*z* 750.31 putatively assigned to the [M + H]^+^ ion for 3′-*N*-debenzoylpaclitaxel made biocatalytically (Figure 2) that was not present when Mg^2+^ was omitted from the assay.

### Molecular Modeling of the BAPT-Catalyzed (2*R*,3*S*)-Isoserinyl Acyltransfer Reaction

A homology model of BAPT was constructed using the SWISS-MODEL^49^ program based on a native HCT (hydroxycinnamoyl-coenzyme A shikimate/quinate hydroxycinnamoyltransferase) from *Coffea canephora* (PDB ID: 4G0B) within the BAHD acyltransferase family.^50^ The Mg^2+^ ion, the Gaussian-optimized baccatin III (**2a**) and (2*R*,3*S*)-phenylisoserinyl CoA (**1**) were docked to the reaction site using AutoDock Vina^51^ and UCSF Chimera^51,52^ to visualize and analyze all the binding poses. MD simulations in this study conducted a thermodynamics analysis on a series of conformations accessible to **2a** and **1** while docked in BAPT. The intrinsic intermolecular and intramolecular stabilization energies of Mg^2+^ and the ligands were calculated within the context of the proximate residues in the enzyme active site. The simulations found several low-energy local minima that were reasonably optimized conformational snapshots. These snapshots aided in finding low-energy, catalytically competent structural conformations, one of which suggested that the BAPT active site accommodates Mg^2+^ in a hexacoordinated complex with the C4-carbonyl and ester oxygens of baccatin III, the N (amino) and O (hydroxyl) atoms of the isoserinyl side chain and water (Figure 3).

**Scheme 4:**
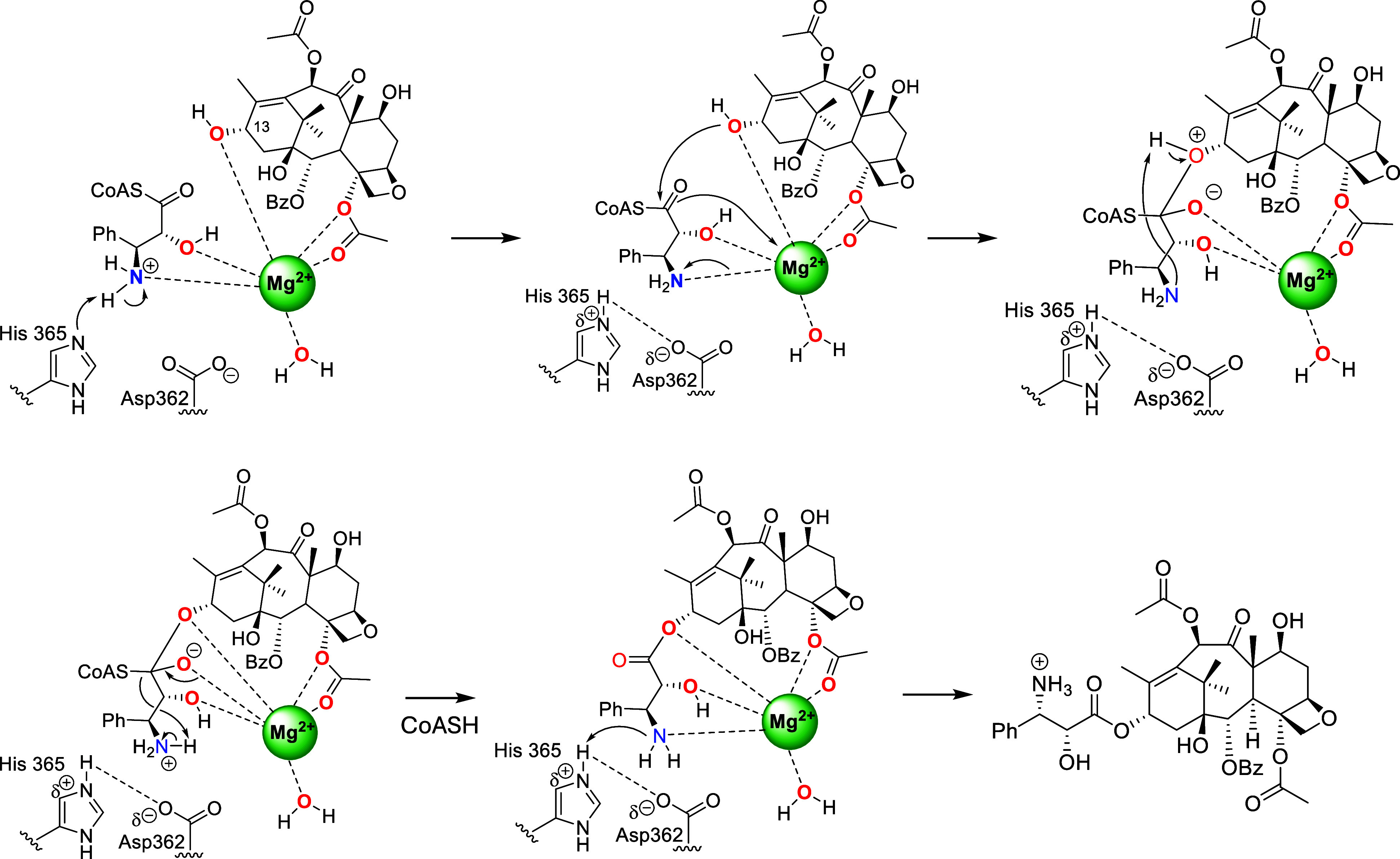
Proposed Mg^2+^-Dependent 13-*O*-Acylation Mechanism of BAPT Catalysis

The low-energy snapshot also shows that Mg^2+^ liberates the C13-hydroxyl group for nucleophilic attack by disrupting the purported intramolecular H-bond with the C4 acetate. The conformational pose of the Mg^2+^ and the reactive ligands places the C13-hydroxyl 3.9 Å from the reactive carbonyl group of the acyl CoA substrate, primed for acyl group transfer (Figure 3). While an earlier study highlighted that a substrate-assisted catalysis mechanism for BAPT was plausible,^24^ a modified mechanism for BAPT activation by Mg^2+^ is proposed where the Lewis acidity of the central ion organizes the BAPT active site and facilitates the H-transfer processes involving the β-amino group of the isoserinyl CoA side chain (Scheme 4). With the assay buffer at pH 7.4, the protonated amino group of the phenylisoserinyl CoA needs to be neutralized (by an unknown mechanism) so it can interact with Mg^2+^ through a proposed, weak dative-bond interaction. In its neutral state, the amino group can engage in concerted, substrate-assisted proton transfer to activate the C13-hydroxyl for nucleophilic displacement of the CoASH via an oxyanion tetrahedral intermediate (Scheme 4).

### Producing (2*R*,3*S*)-3-(1′,1′-Dimethylvinyl)isoserinyl CoA Biocatalytically

**Figure 4:**
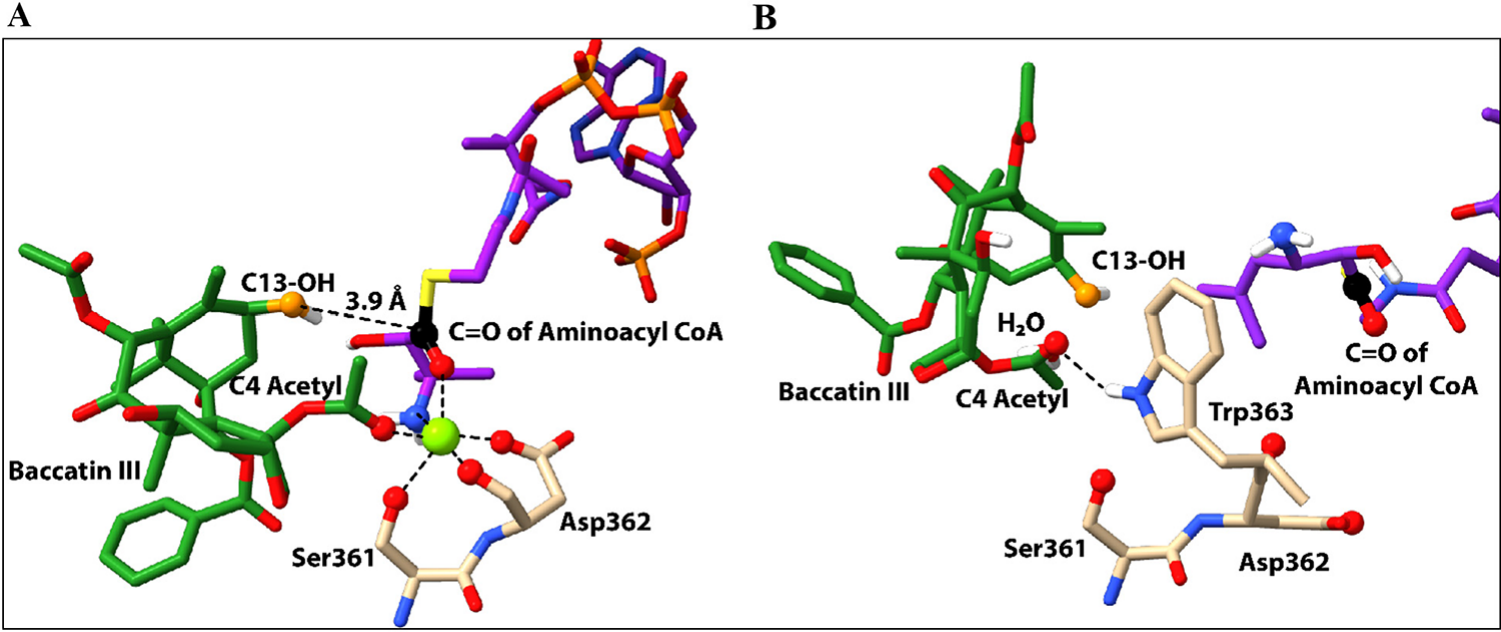
Structures of baccatin III (**2a**) (green sticks), 3-(1′,1′-dimethylvinyl)isoserinyl CoA (**8**) (purple sticks, partial structure) with (A) Mg^2+^ (green sphere) and (B) without Mg^2+^ within the BAPT active site resulting from MD simulations. The atoms estimated to form a hexacoordinated complex are drawn as spheres and include contributions from residues Ser361 and Asp362. The donor carbonyl reaction center of the CoA thioester and the acceptor C13-hydroxyl of baccatin III are shown as black and orange balls, respectively.

After confirming that BAPT catalyzed the Mg^2+^-dependent coupling of phenylisoserinyl with baccatin III, additional MD simulations of BAPT with baccatin III (**2a**) and (2*R*,3*S*)-3-(1′,1′-dimethylvinyl)isoserinyl CoA (**8**) ligands, with and without Mg^2+^, showed compelling support that the non-natural CoA substrate would likely be turned over by BAPT (Figure 4A). The conformational pose of the Mg^2+^ and the reactive ligands shows the C13-hydroxyl ∼3.9 Å from the reactive carbonyl group of the acyl CoA substrate, primed for acyl group transfer (Figure 4A). The low-energy conformational snapshot generated by MD simulation shows that in the BAPT/metal complex, Mg^2+^ coordinates with active site residues Ser361 and Asp362 when 3-(1′,1′-dimethylvinyl)isoserinyl CoA (**8**) is used in the simulation in place of phenylisoserinyl CoA (cf. Figure 3). The different steric demands of the dimethylvinyl compared to the phenyl ring caused the corresponding acyl CoA substrates to adopt conformationally distinct low-energy poses in their interactions with the BAPT/Mg^2+^ complex.

When Mg^2+^ was not included in the simulation, the BAPT/ligand structures showed a disorganized, catalytically unproductive model (Figure 4B). While the intramolecular H-bond within baccatin III was disrupted by other active site elements besides Mg^2+^, such as water, to liberate the C13–OH for nucleophilic attack on the acyl CoA, the absence of Mg^2+^ and the conserved histidine residue caused the calculated minimal spacing between the C13–OH acceptor of baccatin III and the carbonyl functional group of the acyl CoA to increase beyond a constructive collision distance (Figure 4B). These MD simulation predictions are consistent with the experimental results described herein.

We then exercised a proof-of-principle analysis to assess if the BAPT could turnover (2*R*,3*S*)-3-(1′,1′-dimethylvinyl)isoserinyl CoA (**8**), acylate baccatin III analogs, and, as an added benefit, make precursors of next-generation paclitaxel analogs effective against multiple-drug resistant cancer cells.^53^

**Scheme 5:**
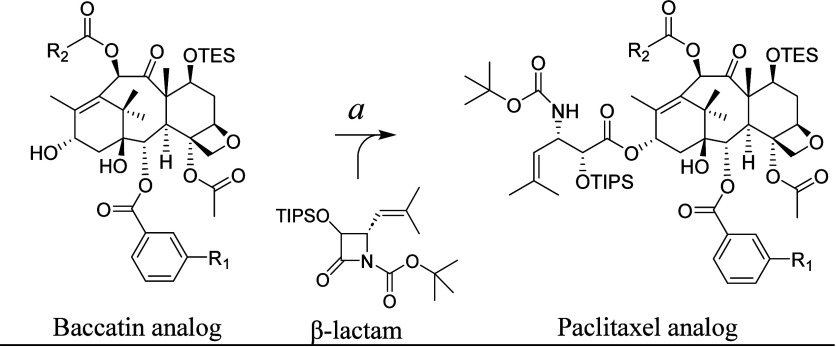
An Example Step in the Semisynthesis of a Paclitaxel Analog Where a Baccatin Analog Directly Ring-Opens a β-Lactam Intermediate (a) LiHMDS (1.5 equiv), dry THF, −40 °C, 2 h. R_1_ (alkyl, halo, or alkoxy) and R_2_ (typically alkyl) are variables. TES: triethylsilyl; TIPS: triisopropylsilyl.

To setup these assays, (2*R*,3*S*)-3-(1′,1′-dimethylvinyl)isoserinyl CoA (**8**) was semisynthesized following an established approach used to synthesize alkyl/arylisoserinyl CoA racemates via β-lactams assembled by a conrotatory Staudinger [2 + 2] cycloaddition of an imine and ketene.^27,54,55^ In the commonly used semisynthetic assembly of paclitaxel analogs, the β-lactam is directly ring-opened by the C13-hydroxyl nucleophile of the baccatin III coupling partner to install the isoserine side chain (Scheme 5).^25,53^

By contrast, in biocatalysis studies using the function of BAPT to assemble paclitaxel analogs, the β-lactam precursors were acid hydrolyzed to their corresponding isoserine racemates, and the carboxylic acids were converted to their CoA thioesters using a truncated TycA module (PheAT) of a nonribosomal peptide synthase (NRPS), tyrocidine synthase, from *Bacillus brevis* to function as an isoserinyl CoA ligase (Scheme 2).^26−28^ In this study, immobilized CAL-B (lipase B from *Candida antarctica*) was added as a stereospecific resolution step to hydrolyze the synthetically derived β-lactam to the (2*R*,3*S*)-3-(1′,1′-dimethylvinyl)isoserine isomer (**7**). Earlier studies showed immobilized CAL-B-catalyzed β-lactam ring cleavage with high enantioselectivities for (2*R*,3*S*)-isoserine analogs.^32^ The choice to include the stereospecific resolution step was driven by results from an earlier study identifying that PheAT stereospecifically thioesterified (2*R*,3*S*)-arylisoserine isomers, while the (2*S*,3*R*)-enantiomer was not turned over.^26,27^

PheAT was incubated with (2*R*,3*S*)-3-(1′,1′-dimethylvinyl)isoserine (**7**), CoA, ATP, and MgCl_2_·(6H_2_O). The biosynthetically derived thioester product was analyzed by LC/ESI-MS/MS (negative-ion mode) with selected-ion monitoring set for *m*/*z* 907.09, which putatively identified (2*R*,3*S*)-3-(1′,1′-dimethylvinyl)isoserinyl CoA (**8**) (Figure S9). The Michaelis–Menten kinetics parameters of PheAT catalysis for (2*R*,3*S*)-3-(1′,1′-dimethylvinyl)isoserine (**7**) under steady-state conditions were found to be *k*_cat_ = 0.5 min^–1^ and *K*_M_ = 112 μM. These calculated parameters of PheAT were used as a guide to scale up (mg-laboratory scale) the production of **8** and confirm its structure using NMR (Figures S11–S13 of the Supporting Information).

#### Laboratory Scale-Up

The assay with **7** and PheAT was scaled up to obtain 23 mg (46% yield based on the isoserine) of the thioester product **8** for downstream biocatalysis reactions. The ^1^H NMR spectra of purified biocatalysis-derived product had chemical shifts of the isoserinyl side chain at δ 4.82 (H2’), δ 4.21 (H3′), and δ 5.12 (H4’) (Figure S9 of the Supporting Information) that were upfield of those for the (2*R*,3*S*)-3-(1′,1′-dimethylvinyl)isoserine (**7**) at δ 5.18, δ 4.57, and δ 5.42, respectively (Figures 6 and S6, S11 of the Supporting Information), suggesting the exchange of a carboxylate to a thioester linkage catalyzed by PheAT. The ^13^C NMR chemical shift (δ 198) for the carbonyl (C1′) (Figure S12 of the Supporting Information) of **8** was shifted downfield compared to that for the isoserine **7** (δ 175) starting material (Figure S7 of the Supporting Information), which further supported the thioesterfication catalysis by PheAT. Moreover, LC/ESI-MS/MS monoisotopic-mass analysis verified a biocatalyzed product of the correct molecular weight of [M-H]^−1^ at *m*/*z* 907.0951 for **8**.

### Evaluating BAPT Activity with (2*R*,3*S*)-3-(1′,1′-Dimethylvinyl)isoserinyl CoA, Baccatin III, and Mg^2+^

**Figure 5:**
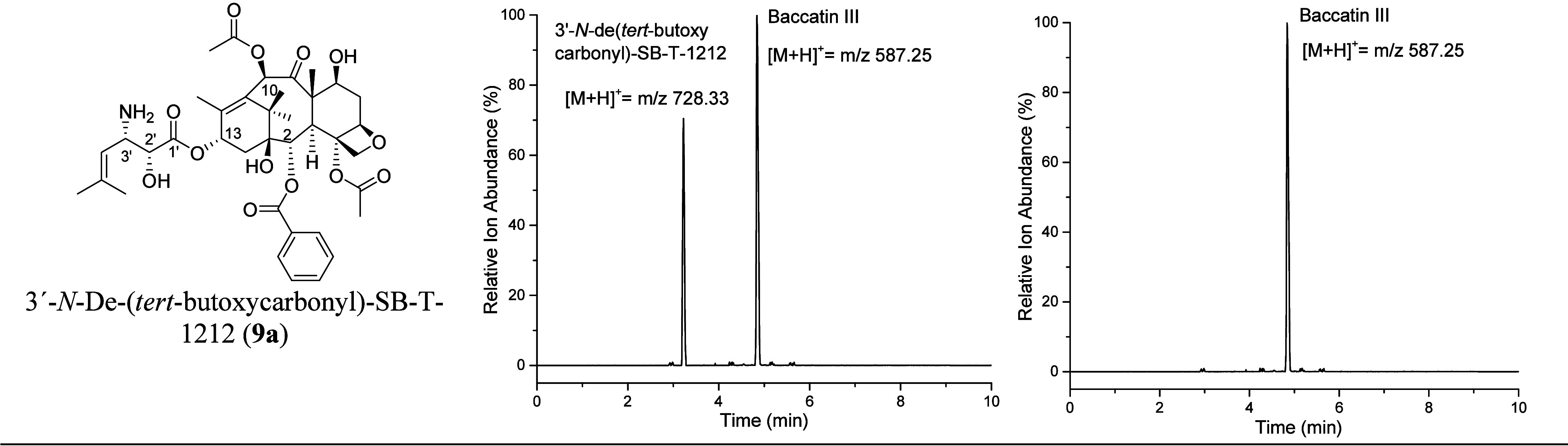
Putative product 3′-*N*-de-(*tert*-butoxycarbonyl)-SB-T-1212 (**9a**) catalyzed by BAPT from baccatin III (**2a**) and 3-(1′,1′-dimethylvinyl)isoserinyl CoA (**8**). (A) LC/ESI-MS/MS in selected-ion mode scanning for [M + H]^+^ ions in an aliquot of the reaction pool after BAPT catalysis assay with MgCl_2_ and (B) without MgCl_2_.

**8** was incubated with baccatin III, BAPT, and the Mg^2+^ cofactor, using the same conditions as the phenylisoserinyl coupling assay. Selected ion *m*/z 728.33 was identified in the LC/ESI-MS/MS profile and putatively assigned to the [M + H]^+^ ion for 13-*O*-[3′-*N*-deBoc-(2*R*,3*S*)-3-(1′,1′-dimethylvinyl)isoserinyl]baccatin III (**9a**) (referred to herein as 3′-*N*-de(*tert*-butoxycarbonyl)-SB-T-1212 or 3′-*N*-deBoc-SB-T-1212) (Figure 5). The “SB-T″ taxanes designation is derived from a series of new-generation chemotherapeutic compounds developed at Stony Brook University (Stony Brook, NY).^56,57^

**Figure 6:**
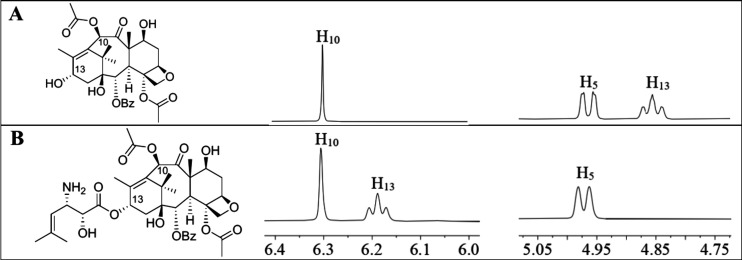
Partial ^1^H NMR spectra of (A) Baccatin III and (B) 3′-*N*-de-(*tert-*butoxycarbonyl)-SB-T-1212.

Obtaining a biocatalysis-derived product with a mass consistent with that of SB-Taxane **9a** motivated us to scale up the production level for further product characterization. The assay with the isoserinyl CoA (**8**), BAPT, Mg^2+^, and baccatin III was scaled up to obtain 18 mg (42% yield based on the baccatin III) of **9a**. The ^1^H NMR spectra of purified biocatalysis products support that BAPT selectively acylated the C13 hydroxyl. The H13 chemical shift (δ 6.19) was shifted downfield for the biocatalyzed products compared to that for baccatin III (δ 4.97) (Figure 6).

**Scheme 6:**
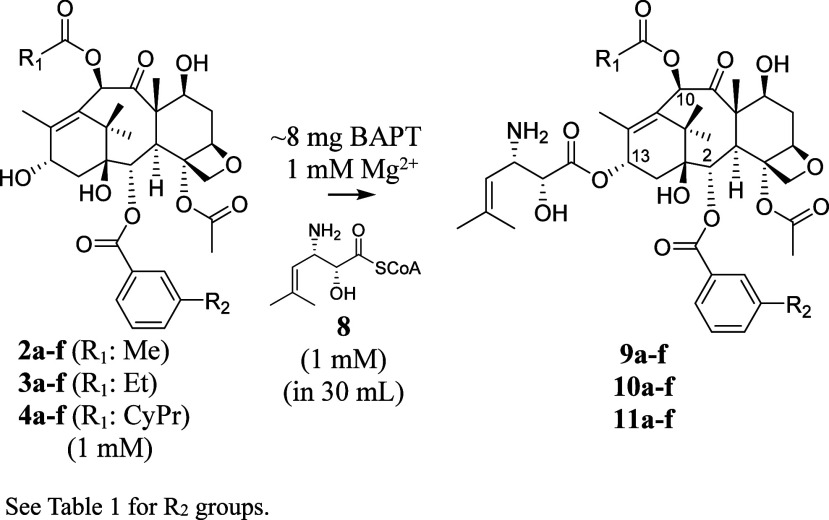
Proposed Biocatalytic Coupling of 3-(1′,1′-Dimethylvinyl)isoserinyl CoA (**8**) and Baccatin III Analogs (**2**, **3**, and **4** (**a**–**f**)) (cf. Table 1) by BAPT Catalysis to Make SB-Taxanes (**9**, **10**, and **11** (**a**–**f**))*^a^*

Recent structure–activity relationship studies have provided sufficient evidence that new-generation paclitaxel analogs with modifications of the parent drug at C2 (benzoyl replaced with *m*-(trifluoromethyl or difluoromethoxy)benzoyl), C10 (acetyl replaced with cyclopropane carbonyl or propionyl), and C13 (*N*-benzoyl phenylisoserinyl replaced with *N*-Boc-(1,1-dimethylvinyl)isoserine) exhibit higher antineoplastic activity over paclitaxel against the drug-sensitive and multiple drug-resistant cancer cells.^58,59^ Herein, BAPT catalysis was used to couple the isoserinyl moiety from its activated CoA thioester **8**, derived semibiocatalytically, with different baccatin III analogs, also derived semibiocatalytically in an earlier study (Scheme 6)^30^ to make precursors of new-generation taxanes. (cf. Schemes 2 and 5).

### Kinetic Evaluation of BAPT with (2*R*,3*S*)-3-(1′,1′-Dimethylvinyl)isoserinyl CoA and Taxanes

The *K*_M_ and *k*_cat_ values of the BAPT acylation catalysis reaction were calculated under steady-state conditions by incubating purified BAPT with various concentrations of (2*R*,3*S*)-3-(1′,1′-dimethylvinyl)isoserinyl CoA, baccatin III and its analogs, and MgCl_2_·(6H_2_O). The catalytic efficiency (*k*_cat_/*K*_M_) values, driven mostly by differences in *k*_cat_, of BAPT for baccatin III and its C10-acyl analogs with a C2-benzoyl group (**2a**, **3a**, and **4a**) (see Table 1) were similar to those for the analogs with a 3-F benzoyl group at C2 (**2b**, **3b**, and **4b**), respectively (see Scheme 6 for numbering). The hydrogen and fluoride bioisosteres on the C2-aryl ring likely did not affect the most catalytically competent conformation of the baccatin substrate, resulting in similar catalytic efficiency numbers. However, within the **2a**, **3a**, and **4a** and **2b**, **3b**, and **4b** series, the catalytic efficiencies decreased slightly with increasing steric capacity (acetyl < propionyl < cyclopropane carbonyl) at C10 of the baccatin III substrate, suggesting that the BAPT reaction is sensitive to the acyl group size at C10.

The catalytic efficiency (*k*_cat_/*K*_M_) values tended to decrease with increasing steric volume when the C2-benzoyl group was substituted with a *meta*-Cl (**c**), −OCH_3_ (**d**), −OCHF_2_ (**e**), or −OCF_3_ (**f**), with C2-(*m*-OCH_3_)benzoyl baccatin III analogs (**2d**, **3d**, and **4d**) causing the BAPT efficiency (27, 17, and 14 s^–1^ M^–1^, respectively) to suffer the most compared to the other C2-(*m*-substituted)benzoyl baccatin III analogs within the respective groups. The generally accepted coplanar conformation adopted by the OCH_3_ group (i.e., the ether oxygen and carbon are nearly coplanar with the aromatic ring)^60^ likely amplifies the steric interactions between the substrate and the BAPT active site residues. Also, the baccatin analogs with 3-OCHF_2_-benzoyl group (**2e**, **3e**, and **4e**) were turned over faster than those with 3-OCF_3_-benzoyl (**2f**, **3f**, and **4f**) at C2. The earlier X-ray and MD studies also show that the 3-OCHF_2_ group has more conformational (in-/out-of)-plane flexibility than 3-OCF_3_, which prefers an orthogonal conformation (i.e., the ether oxygen and carbon bond is nearly orthogonal to the aromatic ring plane).^61^ The flexibility of the OCHF_2_ to vacillate between in-plane and out-of-plane stabilization on the phenyl ring of the baccatin III analogs likely increases its ability to adopt a favorable catalytic binding configuration, and thus enabling **2e**, **3e**, and **4e** to turnover more efficiently than their OCF_3_ and OCH_3_ bioisosteric counterparts.

#### Biocatalytic Scale-Up of 3′-*N*-De(*tert*-butoxycarbonyl)-SB-T-121(**2**/**3**/**4**) Analogs

BAPT catalyzed the transfer of a non-natural acyl group from 3-(1′,1′-dimethylvinyl)isoserinyl CoA to a natural substrate acceptor baccatin III and its analogs (**2**, **3**, and **4** (**a**–**f**)) as evidenced in kinetic studies. The scale-up reactions used a combination of methods; one directly transferred the isoserinyl moiety from its acyl CoA to the baccatin acceptor with the Mg^2+^ cofactor present in an assay. Another method from an earlier study demonstrated a complementary proof-of-concept approach to recycle an acyl CoA with a CoA ligase to increase the titers of biocatalyze acylated taxane;^62^ those principles were employed in this study. To not deplete the precious 3-(1′,1′-dimethylvinyl)isoserinyl CoA (**8**) during the reaction, PheAT (functioning as an isoserinyl CoA ligase), Mg^2+^ (needed for PheAT and BAPT catalysis), ATP, 3-(1′,1′-dimethylvinyl)isoserine, and CoA were preincubated before supplementing the reaction pool with BAPT and the baccatin III acceptor at ∼0.6 mmol (30–35 mg) to recycle the 3-(1′,1′-dimethylvinyl)isoserinyl CoA.

The catalytic efficiency values (Table 2) forecasted that the 3′-*N*-de(*tert*-butoxycarbonyl)-SB-T-121(**2**/**3**/**4**) analogs with a 2-*O*-benzoyl (**9a**–**11a**) and −3-fluorobenzoyl (**9b**–**11b**) would scale the highest (18 mg based on conversion), ranging between ∼23 and ∼42% converted yield (Table 2). The remaining compounds scaled to comparatively lower levels within each C10-acyl series between 6% converted yield (∼3 mg for the 10-*O*-cyclopropane carbonyl-2-*O*-3-methoxybenzoyl analog (**11d**)) and ∼27% converted yield (∼12 mg for the 10-*O*-acetyl-2-*O*-3-chlorobenzoyl analog (**9c**)) with the 2-*O*-3-difluoromethoxybenzoyl analogs (**9e**–**11e**) exceeding expectations, consistent with the kinetic analyses.

**Table 2:**
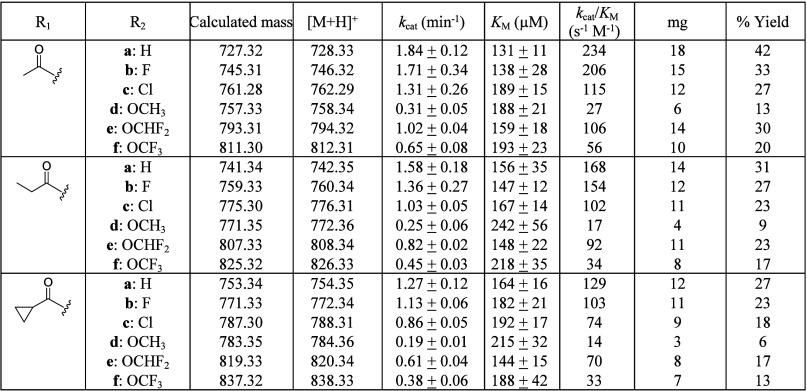
Relative Kinetics of BAPT for 3-(1′,1′-Dimethylvinyl)isoserinyl CoA and Taxane Analogs **2**, **3**, and **4** (**a**–**f**) (cf. Table 1)

## Conclusions

Our earlier sequential two-enzyme assay that contained a Mg^2+^-ATP dependent CoA ligase and BAPT and another study suggesting that an intramolecular H-bond tether in baccatin III (also the BAPT substrate) precluded turnover of a BAHD benzoyltransferase because of an intramolecular hydrogen bond tether^28,29^ informed us that Mg^2+^ ions may also facilitate BAPT catalysis. Choosing Mg^2+^ to disrupt the intramolecular hydrogen bond gave foundational results by imparting a putative substrate-assisted pathway toward stimulating the *Taxus* plant BAHD acyltransferase BAPT, which naturally lacks a critical catalytic histidine residue. To our knowledge, BAPT is the only BAHD enzyme needing a metal cation for activity. MD simulations found low-energy snapshot poses of Mg^2+^-BAPT complexes coordinated with the oxygen and nitrogen atoms of the backbone residues and the isoserinyl CoA and taxane substrates, which supported a potential substrate-assisted mechanism. There is an increasing gap between protein sequences and structures^63^ often created by the intrinsic difficulties of obtaining protein crystal structures. Therefore, the authors used MD simulations to predict that a hexacoordinated Mg^2+^ reorganizes the active site, effectively disentangles an intramolecular H-bond within baccatin III, and liberates the reactive C13-hydroxyl of baccatin III for acylation. These predictions transferred to an effective catalysis combining BAPT and the Mg^2+^ cofactor to guide a highly regioselective 13-*O*-isoserinylation reaction, using non-natural cosubstrates to make penultimate precursors to new-generation taxanes.

The Mg^2+^-ion dependence of BAPT changes the optics of BAHD catalysis that could potentially guide engineering efforts to convert other BAHD acyltransferases to become metal-ion dependent and selectively transfer non-natural β-amino acyl groups to their acceptor substrates. We acknowledge that other multicoordinate cations can potentially organize the ligands and BAPT active site residues to promote catalysis, and these cations will be explored in future studies. As an added benefit, the biocatalytic approach described here provides an alternative route to produce the vital intermediates of the next-generation taxoids by complementing existing synthetic methodology, thus reducing traditional reagents and protecting group manipulations used in the synthetic pipeline. Future studies will explore using the PheAT “CoA ligase” to access more halovinyl-, alkyl-, and aryl-isoserinyl CoA acyl group donors and bypass the need to synthesize these thiol linkages. Conceivably, linear biocatalytic cascades can be constructed, as with PheAT and BAPT in this study, where enzymes upstream on the baccatin III assembly pathway can be reacted in a single reaction vessel without isolating intermediates. Further, we envision product yields can ultimately be scaled through process optimization by adjusting substrate concentrations and biocatalyst selectivity through mutagenesis techniques to reach minimum volumetric productivity acceptable for implementation at an industrial scale.

## Supporting Information Available

Protein purification gel images, NMR profiles, LC-mass spectrometry profiles, and enzyme kinetics plots (PDF)
