# Supplementary material for: Mg2+-Ion Dependence Revealed for a BAHD 13-O-β-Aminoacyltransferase from Taxus Plants
Source: JACS Au. 2024 Sep 30;4(11):4249–62. doi: 10.1021/jacsau.4c00577 (PMC11600153; doi:10.1021/jacsau.4c00577)
Supplement: Supplementary file 1 — au4c00577_si_001.pdf [file au4c00577_si_001.pdf]

Supporting Information

Mg^{2+}-Ion Dependence Revealed for a BAHD 13-O-β-Aminoacyltransferase from *Taxus* Plants

Aimen Al-Hilfi, Zhen Li, Kenneth M. Merz Jr., and Kevin D. Walker*

AUTHOR INFORMATION

Corresponding Author

Kevin D. Walker – *Department of Chemistry, Michigan State University, East Lansing, Michigan 48824, United States; Department of Biochemistry and Molecular Biology, Michigan State University, East Lansing, Michigan, 48824, United States; ORCID: 0000-0001-5208-6692;*

Email: walke284@msu.edu

Authors

Aimen Al-Hilfi – *Department of Chemistry, Michigan State University, East Lansing, Michigan 48824, United States; ORCID: 0009-0000-1044-3698*

Zhen Li – *Department of Chemistry, Michigan State University, East Lansing, Michigan 48824, United States; ORCID: 0000-0001-7947-3887*

Kenneth M. Merz, Jr. – *Department of Chemistry, Michigan State University, East Lansing, Michigan 48824, United States; Department of Biochemistry and Molecular Biology, Michigan State University, East Lansing, Michigan, 48824, United States; ORCID: 0000-0001-9139-5893*

NMR Data for *N*-(*p*-Methoxyphenyl)-3-acetoxy-4-(2-methyl-1-propen-1-yl)azetidin-2-one (5). ^{1}H NMR (500 MHz, $CDCl_3$) δ: 7.28 (d, J = 5.4 Hz, 2H), 6.82 (d, J = 5.1 Hz 2H), 5.71 (d, J = 4.9 Hz, 1H), 5.06 (d, 4.2 Hz, 1H), 4.88 (dd, J = 9.4, 9.1 Hz, 1H), 3.70 (s, 3H), 2.05 (s, 3H), 1.75 (s, 3H), 1.72 (s. 3H) (Figure S1). ^{13}C NMR (126 MHz, $CDCl_3$) δ: 169.35, 161.22, 156.36, 141.95, 130.65, 118.42, 117.49, 114.01, 75.94, 56.35, 55.04, 25.78, 20.37, 18.35 (Figure S2).

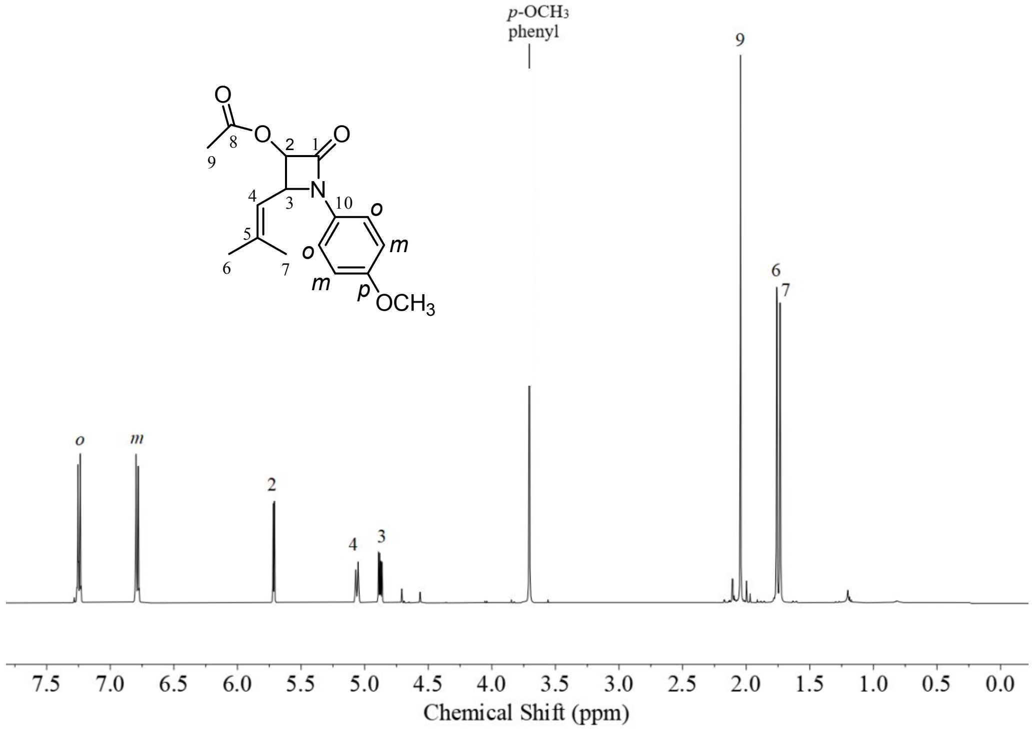

Figure S1. ^{1}H NMR of *N*-(*p*-methoxyphenyl)-3-acetoxy-4-(2-methyl-1-propen-1-yl)azetidin-2-one.

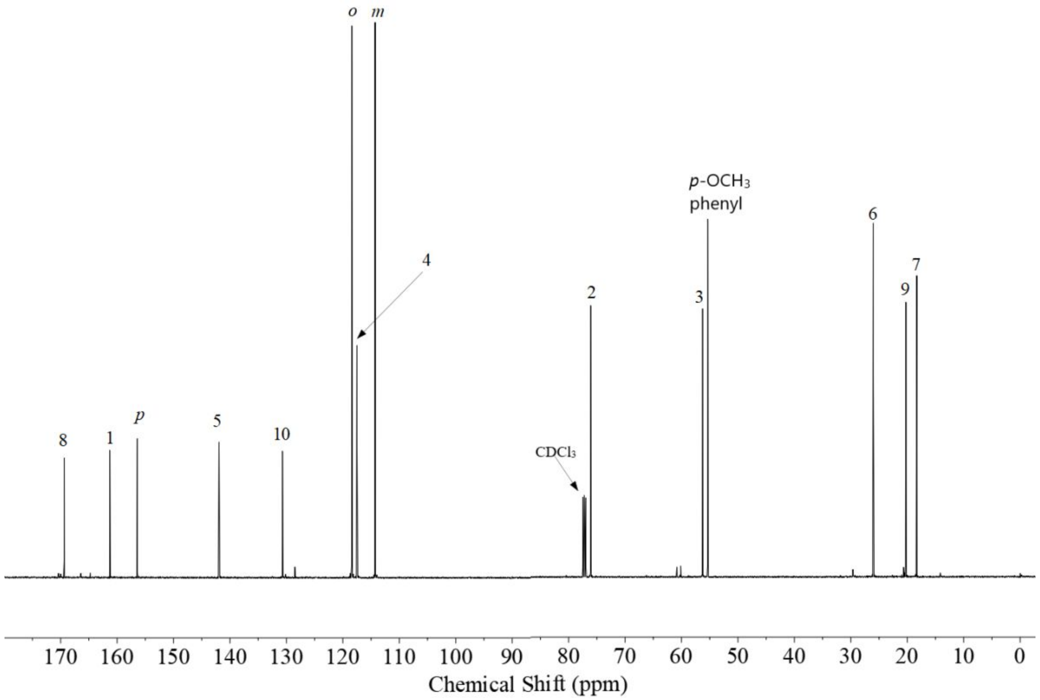

Figure S2. ^{13}C NMR of *N*-(*p*-methoxyphenyl)-3-acetoxy-4-(2-methyl-1-propen1-yl)azetidin-2-one.

NMR Data for 3-acetoxy-4-(2-methyl-1-propen-1-yl) azetidine-2-one (6). ^{1}H NMR (500 MHz, $CDCl_3$) δ: 5.65 (d, J = 4.7 Hz, 1H), 5.06 (d, J = 4.5 Hz, 1H,), 4.56 (dd, J = 9.1, 9.2 Hz, 1H), 2.11 (s, 3H), 1.69 (s, 3H), 1.61 (s. 3H) (Figure S3). ^{13}C NMR (126 MHz, $CDCl_3$) δ: 169.46, 166.36, 141.43, 118.88, 77.68, 52.49, 25.91, 18.26, 17.54 (Figure S4).

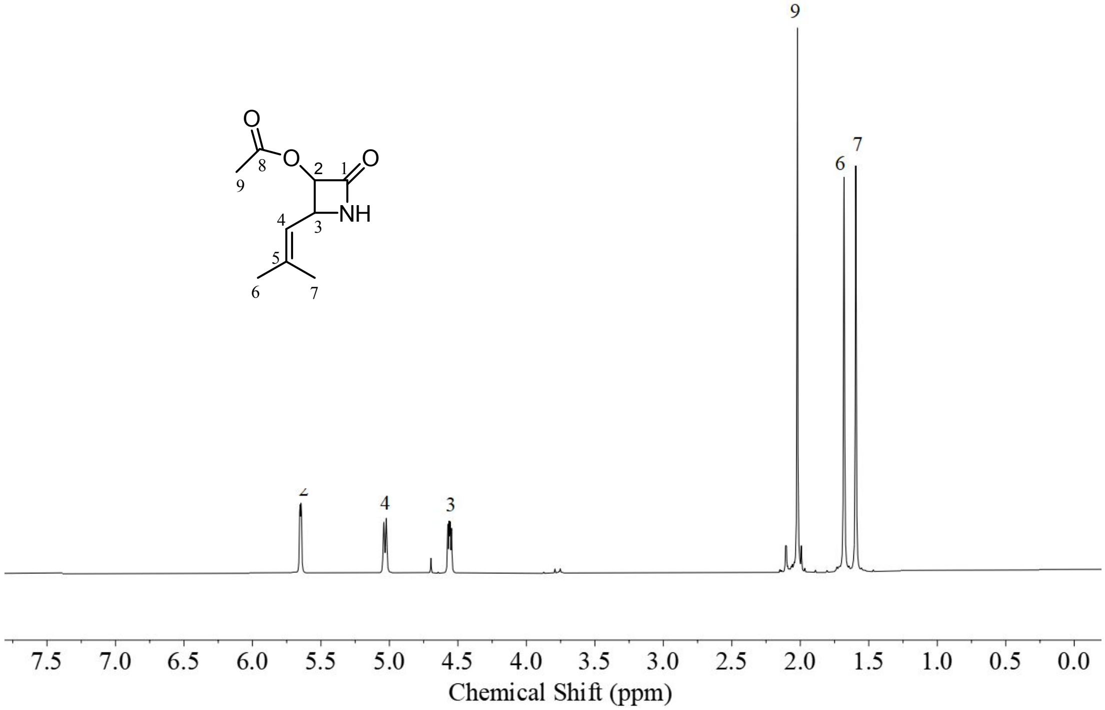

Figure S3. ^{1}H NMR of 3-acetoxy-4-(2-methyl-1-propen1-yl)azetidin-2-one.

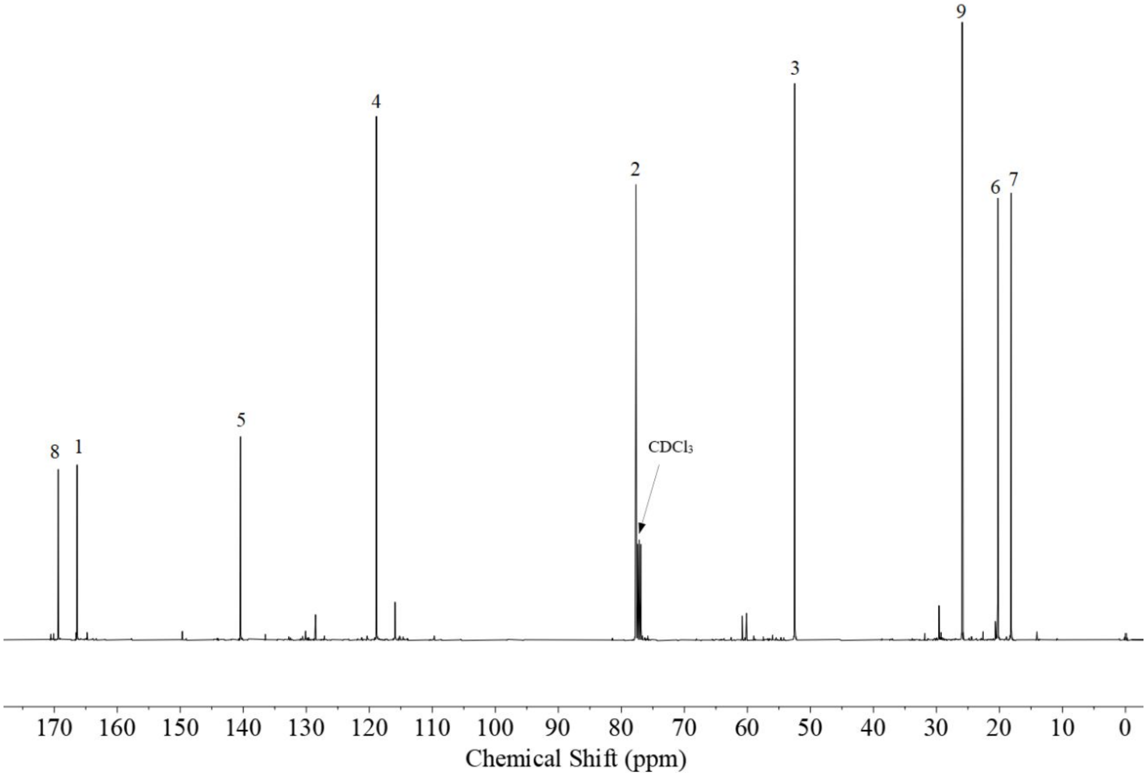

Figure S4. ^{13}C NMR of 3-acetoxy-4-(2-methyl-1-propen1-yl)-azetidin-2-one.

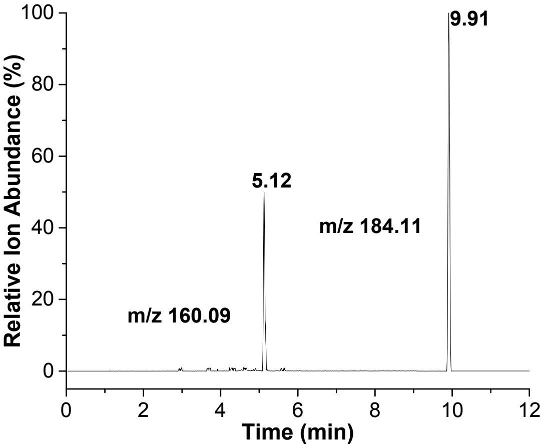

Figure S5. LC/ESI-MS in selected-ion mode scanning the $[M+H]^+$ ions for the putative (2*R*,3*S*)-3-(1',1'-dimethylvinyl)isoserine (**7**) (*m*/*z* 160) and the unreacted azetidinone enantiomer (*m*/*z* 184) of racemate (**6**)

NMR Data for (2*R*,3*S*)-3-(1',1'-dimethylvinyl)isoserine (7). ^{1}H NMR (500 MHz, $CDCl_3$) δ: 5.43 (d, *J* = 4.7 Hz, 1H), 5.11 (d, 4.8, 1H), 4.56 (dd, *J* = 4.7, 4.6 Hz, 1H), 1.78 (s, 3H), 1.69 (s, 3H) (Figure S6). ^{13}C NMR (126 MHz, CDCl3) δ: 169.46, 137.36, 129.48, 75.88, 77.68, 47.12, 22.91, 18.56, 17.14 (Figure S7). LC/ESI-MS monoisotopic exact mass *m/z* 160.0928 $[M + H]^{+}$; calculated for $C_7H_{14}NO_3$: 160.0973.

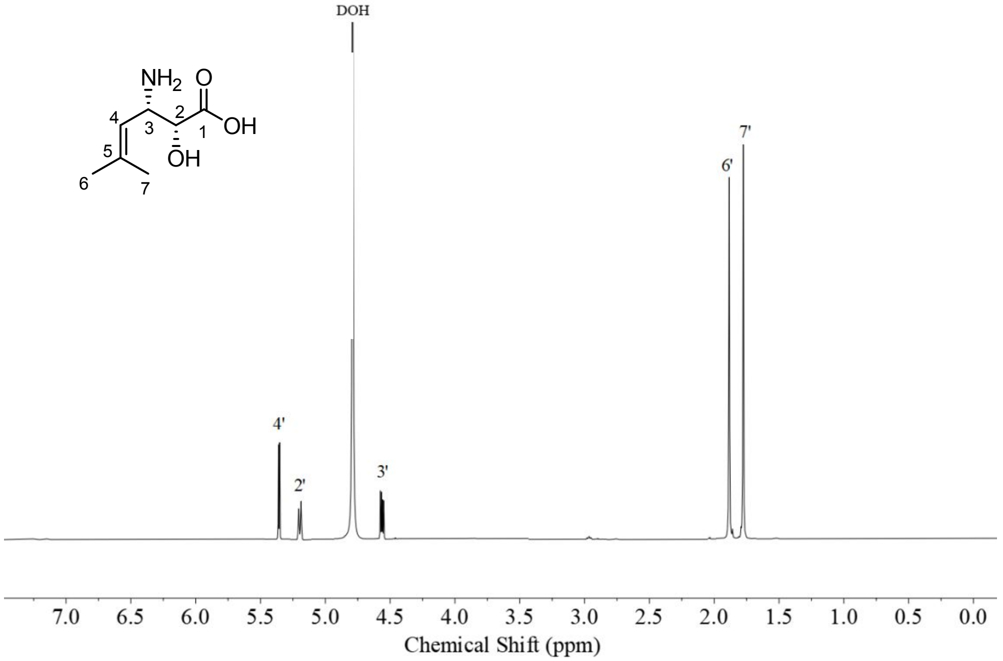

Figure S6. ^{1}H NMR of (2*R*,3*S*)-3-(1',1'-dimethylvinyl)isoserine.

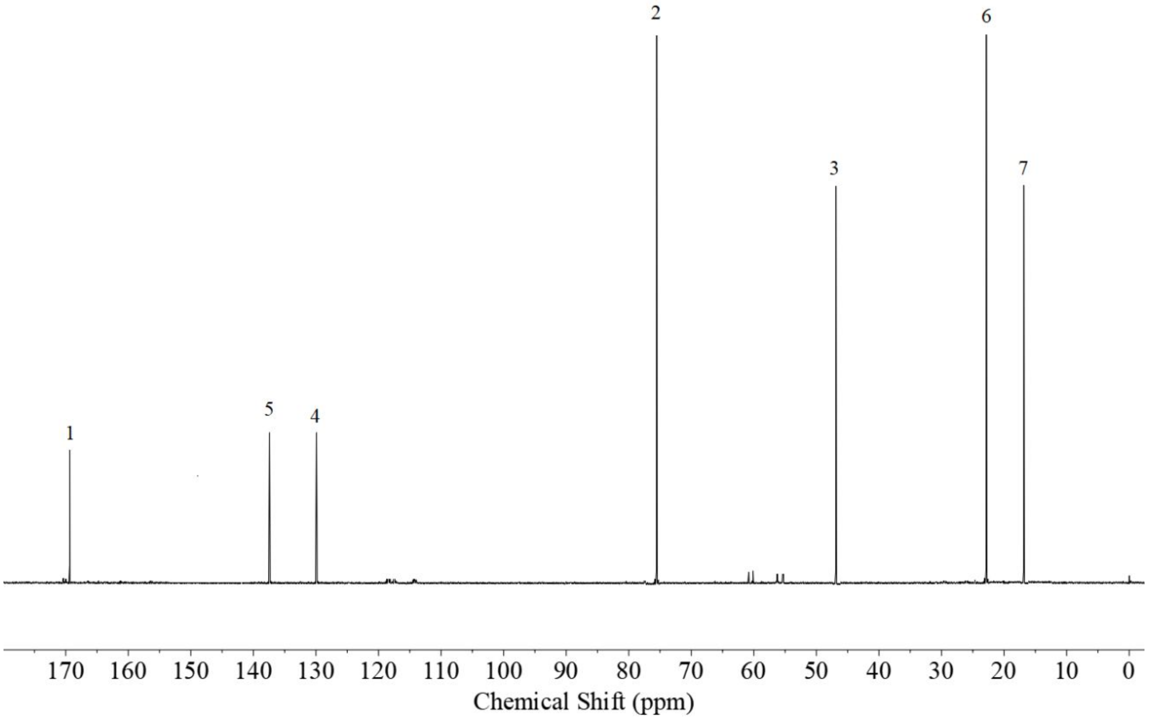

Figure S7. ^{13}C NMR of (2*R*,3*S*)-3-(1',1'-dimethylvinyl)isoserine.

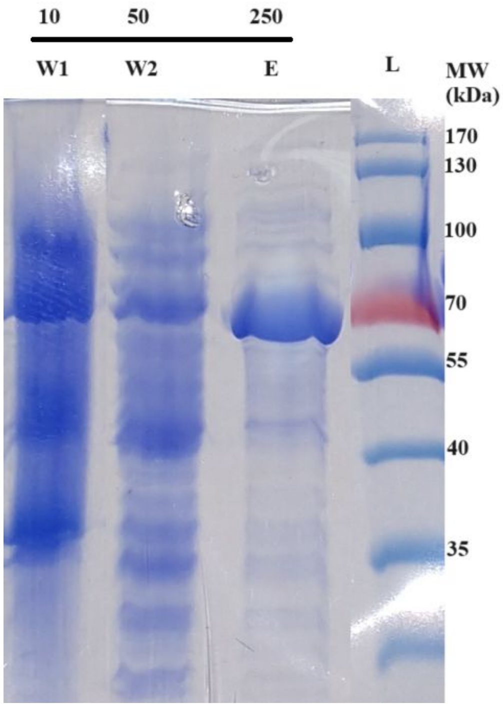

Figure S8. Coomassie Blue stained SDS-PAGE gel of aliquots from the fractions collected from the Ni-NTA affinity exchange column used to purify the PheAT enzyme. Lanes represent protein contained in the Wash Buffer (**W1** and **W2**) and Elution Buffer (**E**) fractions. The numbers above the bar are the mM concentrations of imidazole in the respective buffers. Molecular weight references are in the rightmost lane.

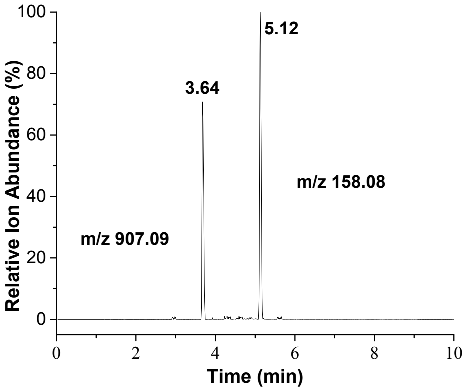

Figure S9. LC/ESI-MS in selected-ion mode scanning the $[M-H]^-$ ions for the putative (2*R*,3*S*)-3-(1',1'-dimethylvinyl)isoserinyl CoA (**8**) (*m*/*z* 907) and the unreacted (2*R*,3*S*)-3-(1',1'-dimethylvinyl)isoserine (*m*/*z* 158) (**7**).

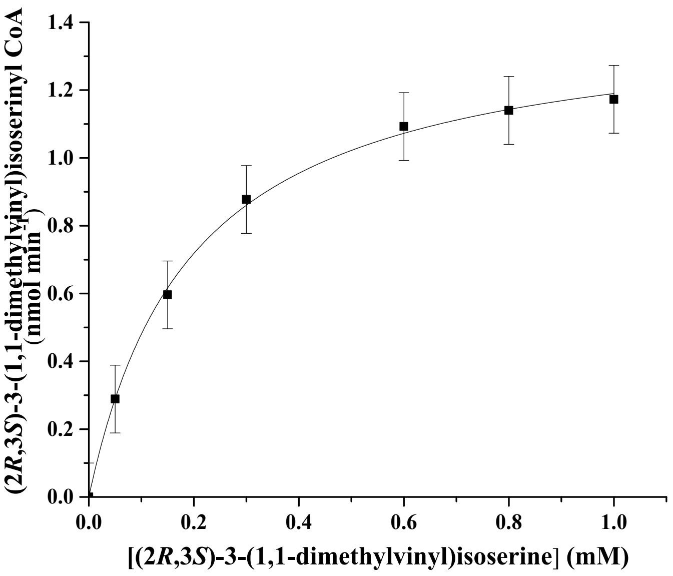

Figure S10. Michaelis-Menten kinetics profile for the turnover of (2*R*,3*S*)-3-(1',1'-dimethylvinyl)isoserine (**7**) to its thioester (**8**) by PheAT catalysis.

NMR Data for (2*R*,3*S*)-3-(1',1'- dimethylvinyl)isoserinyl CoA (8). ^{1}H NMR (500 MHz, $CDCl_3$) δ: 8.39 (s, 1H), 8.11 (s, 1H), 6.04 (d, *J* = 6.2 Hz, 1H), 5.12 (d, *J* = 4.5 Hz, 1H), 4.83 (d, *J* = 4.6, 1H), 4.72 (m, 1H), 4.68 (d, *J* = 5.9 Hz, 1H), 4.47 (t, J = 2.7 Hz, 1H), 4.21 (dd, *J* = 4.7, 4.6 Hz, 1H), 4.11 (m, 2H), 3.84 (s, 1H), 3.73 (dd, *J* = 4.8, 4.6 Hz, 1H), 3.38 (dd, *J* = 4.8, 4.5 Hz, 1H), 3.31 (t, *J* = 6.6 Hz, 2H), 3.17 (t, *J* = 6.6 Hz, 2H), 2.46 (t, *J* = 6.6 Hz, 2H), 2.34 (t, *J* = 6.6 Hz, 2H), 1.82 (s, 3H), 1.69 (s, 3H), 0.86 (s, 6H) (Figure S11). ^{13}C NMR (126 MHz, CDCl3) δ: 198.5, 178.9, 178.6, 152.1, 147.8, 147.6, 141.2, 138.11, 127.84, 117.83, 96.12, 87.52, 81.62. 75.36, 73.45, 64.82, 48.21, 41.23, 37.64, 34.72, 29.95, 21.65, 18.52, 16.42 (Figure S12). LC/ESI-MS monoisotopic exact mass *m/z* 907.1842 $[M - H]^{-1}$; calculated for $C_{28}H_{46}N_8O_{18}P_3S$: 907.1864.

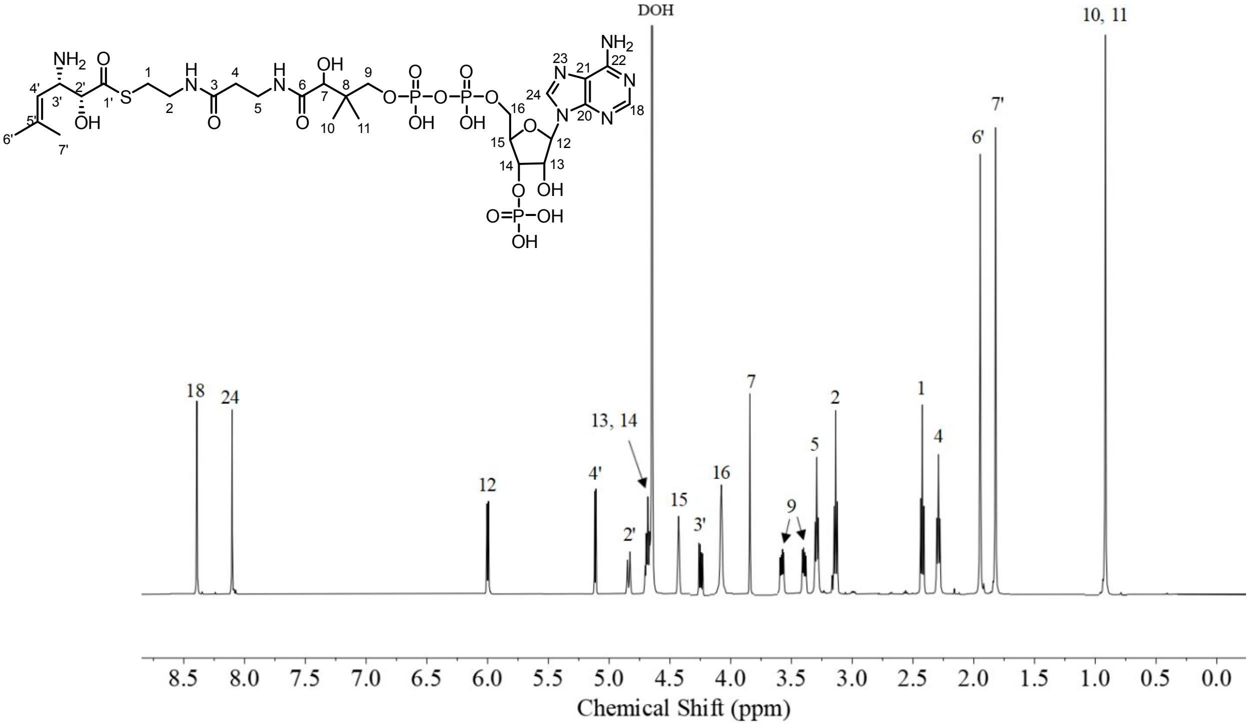

Figure S11. ^{1}H NMR of (2*R*,3*S*)-3-(1',1'-dimethylvinyl)isoserinyl CoA.

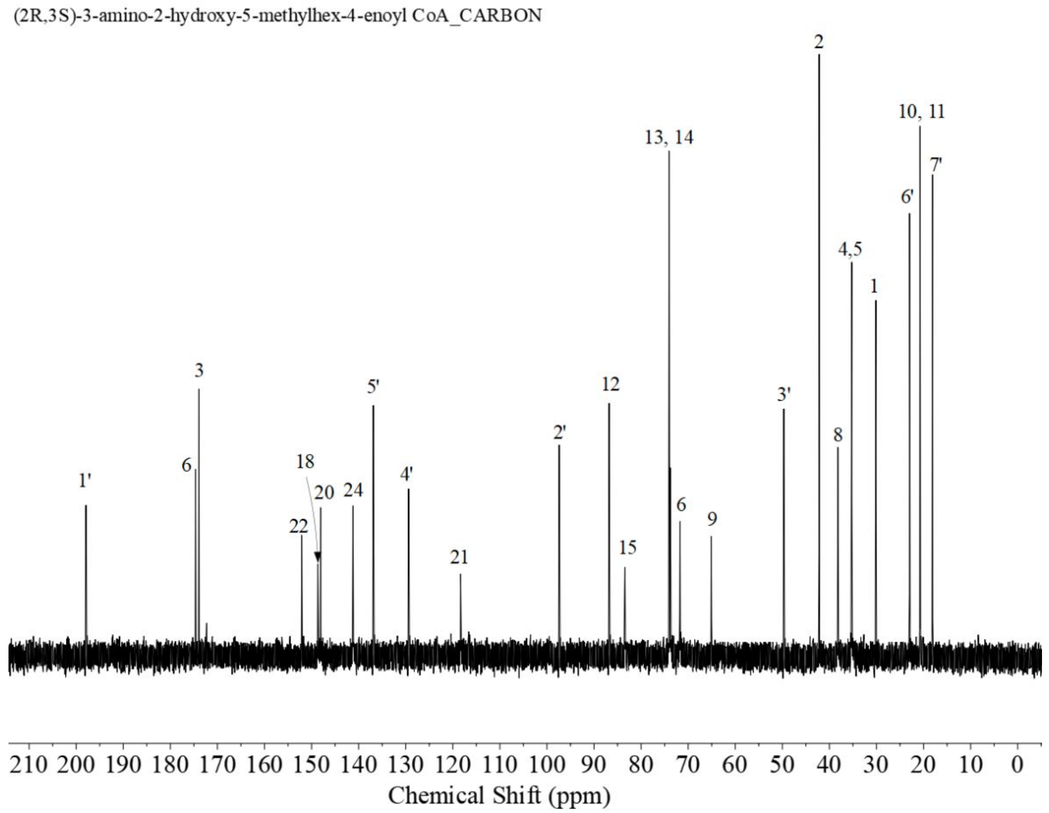

Figure S12. ^{13}C NMR of (2*R*,3*S*)-3-(1',1'-dimethylvinyl)isoserinyl CoA.

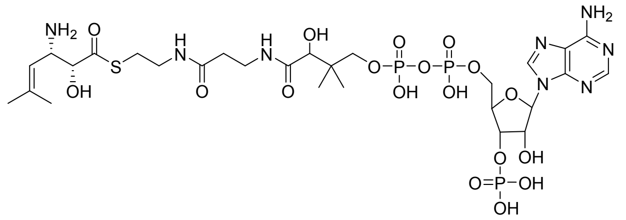

m/z 907.2

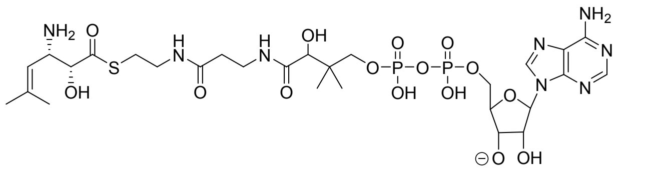

m/z 827.2

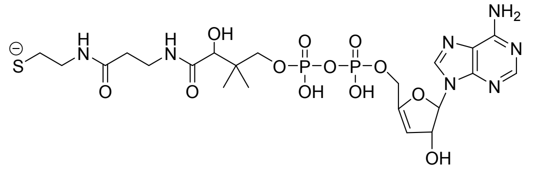

m/z 668.1

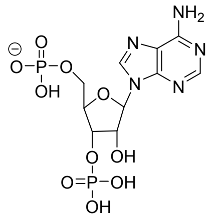

m/z 426.0

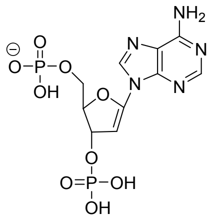

m/z 408.0

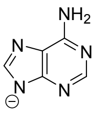

m/z 134.0

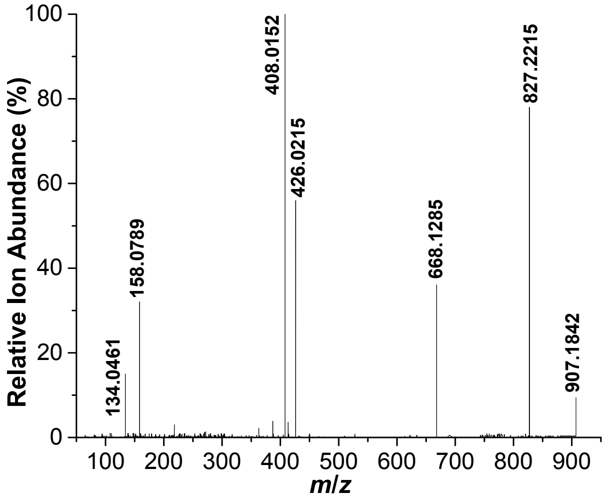

Figure S13. LC/ESI-MS/MS positive-ion mode of purified of (2*R*,3*S*)-3-(1',1'-dimethylvinyl)isoserinyl CoA **(8)**.

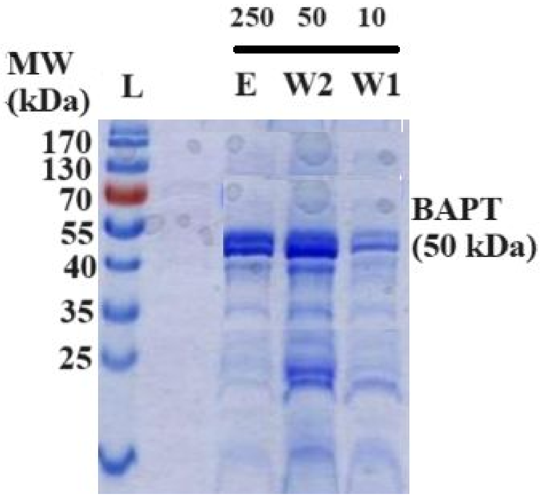

Figure S14. Coomassie Blue stained SDS-PAGE gel of aliquots from the fractions collected from the Ni-NTA affinity exchange column used to purify the BAPT enzyme. Lanes represent protein contained in the Wash Buffer (**W1** and **W2**) and Elution Buffer (**E**) fractions. The numbers above the bar are the mM concentrations of imidazole in the respective buffers. Molecular weight references are in the leftmost lane.

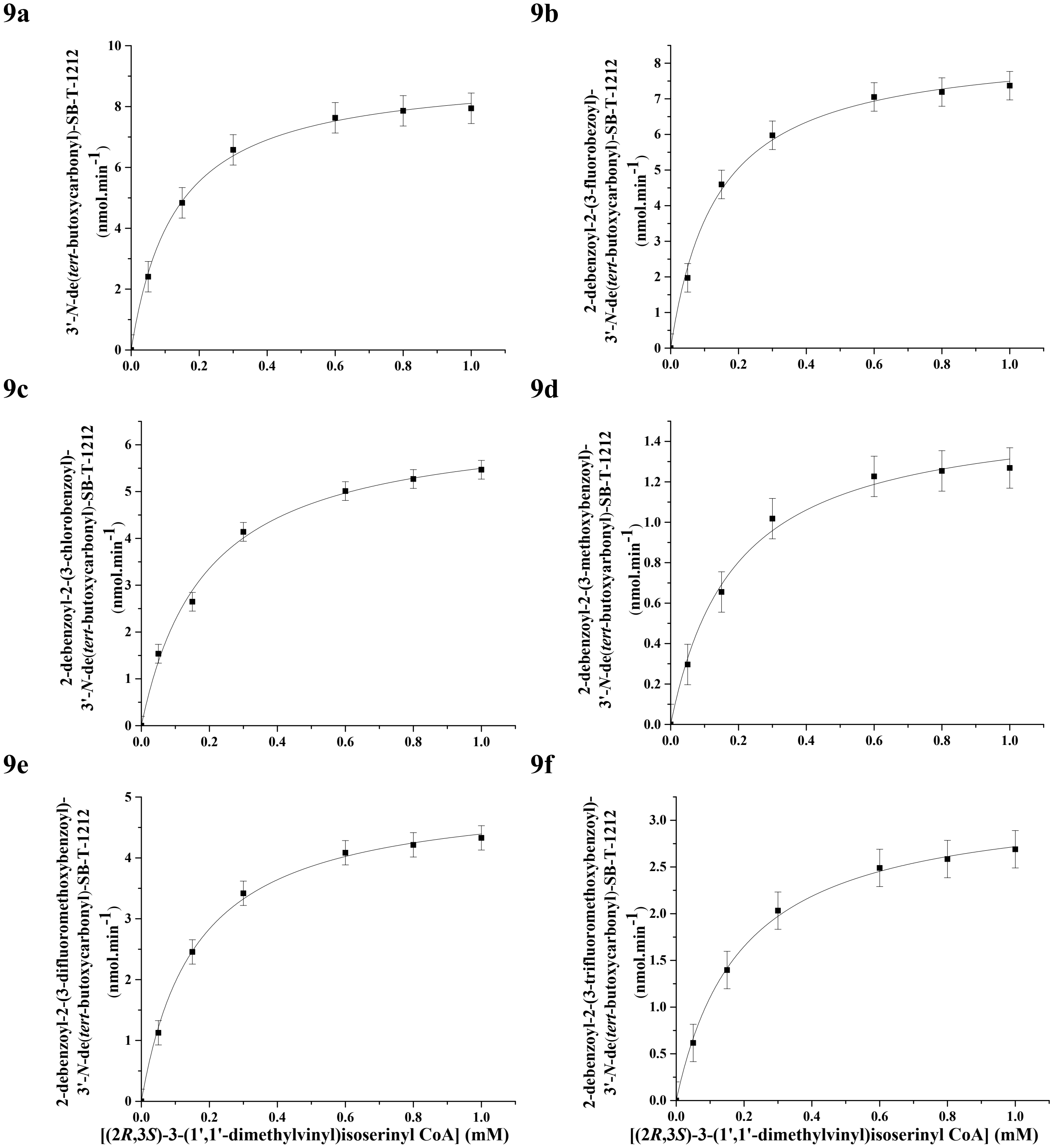

Figure S15. Michaelis-Menten kinetic profiles for the turnover of (2*R*,3*S*)-3-(1',1'-dimethylvinyl)isoserinyl CoA and a baccatin III variant to 3'-*N*-de(*tert*-butoxycarbonyl)-SB-T-1212 analogs **9a-f** by BAPT

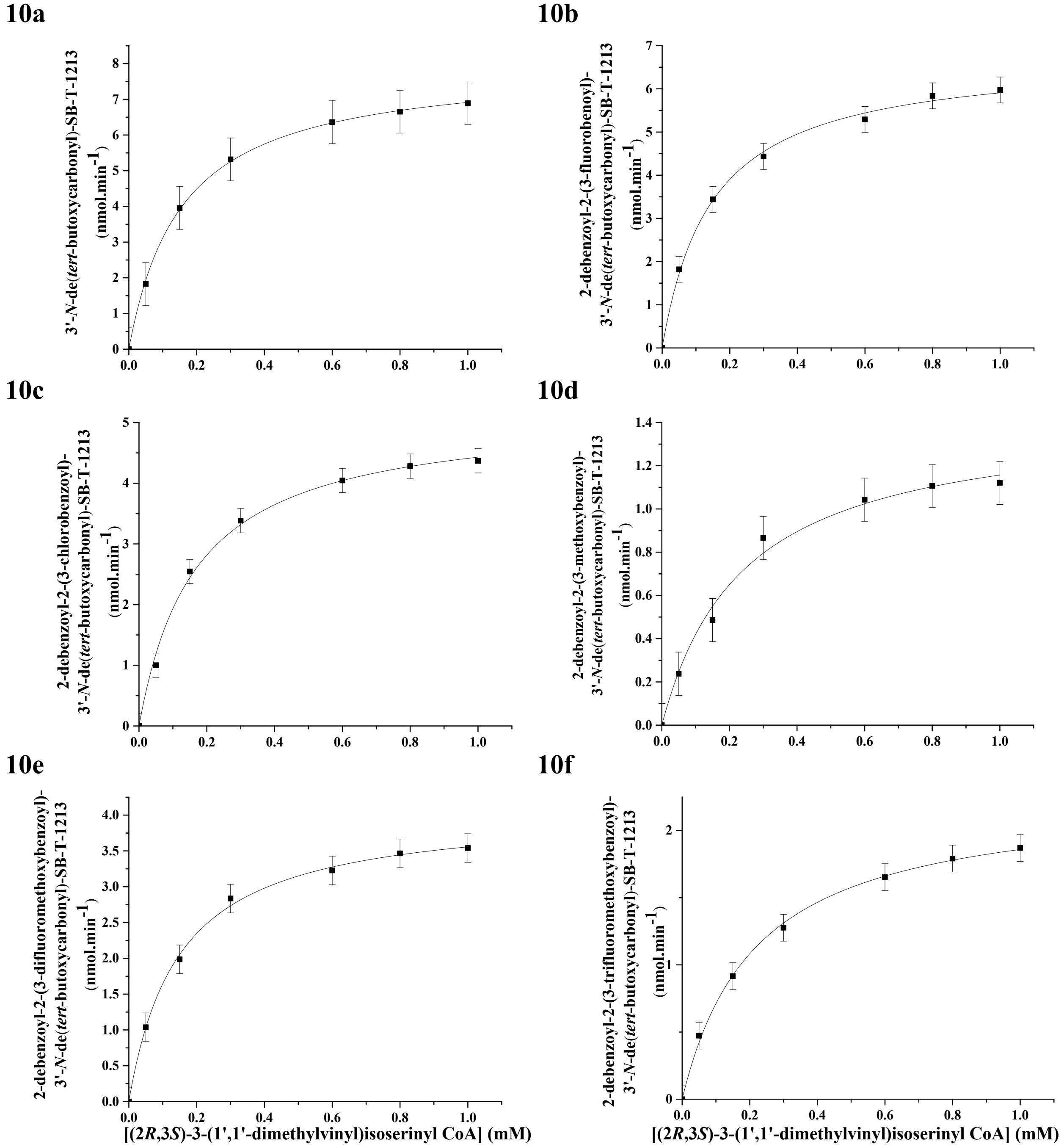

Figure S16. Michaelis-Menten kinetic profiles for the turnover of (2*R*,3*S*)-3-(1',1'-dimethylvinyl)isoserinyl CoA and a baccatin III variant to 3'-*N*-de(*tert*-butoxycarbonyl)-SB-T-1213 analogs **10a-f** by BAPT.

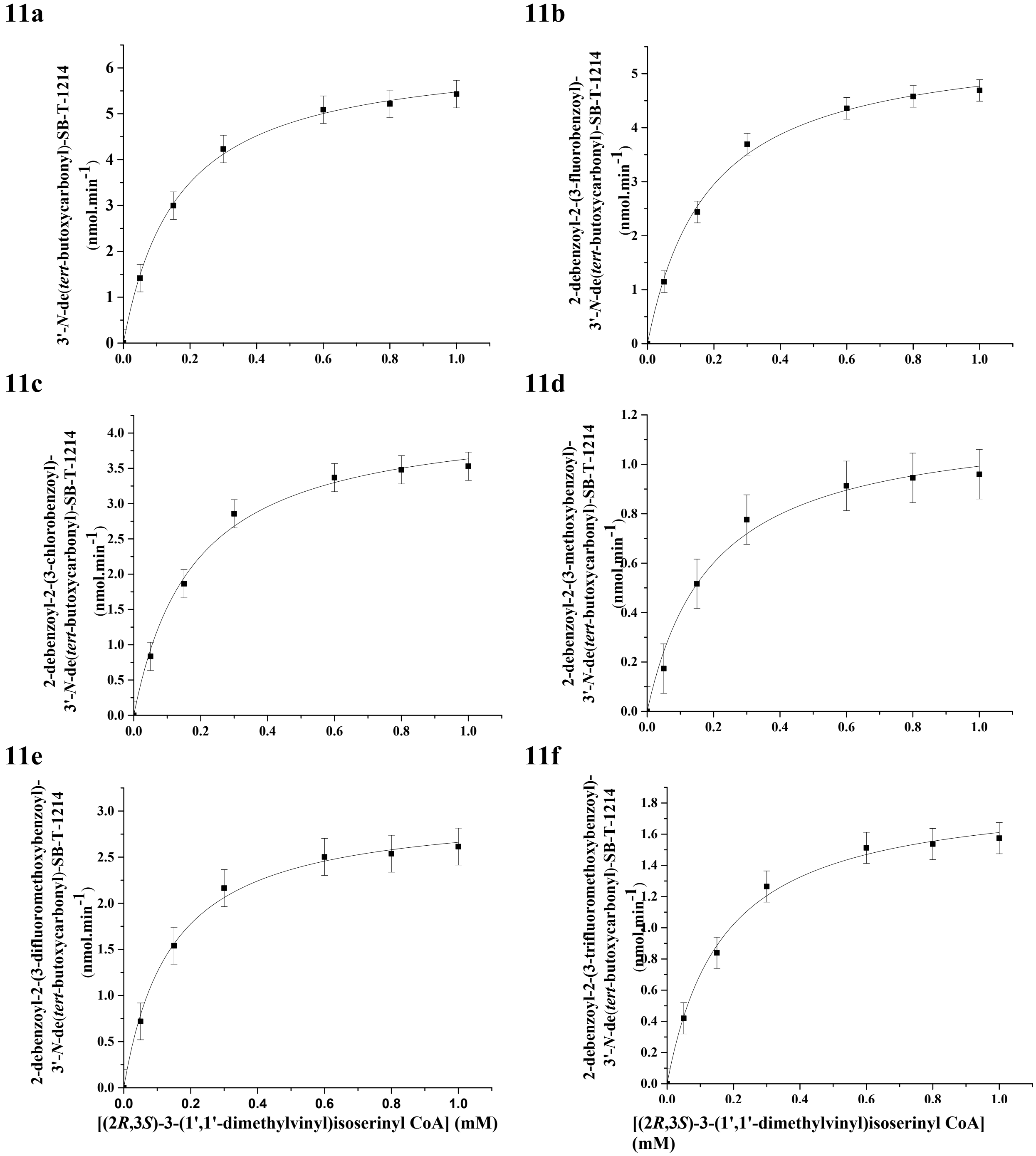

Figure S17 Michaelis-Menten kinetic profiles for the turnover of (2*R*,3*S*)-3-(1',1'-dimethylvinyl)isoserinyl CoA and a baccatin III variant to 3'-*N*-de(*tert*-butoxycarbonyl)-SB-T-1214 analogs **11a-f** by BAPT.

NMR Data for 3′-*N*-de(*tert*-butoxycarbonyl)-SB-T-1212 (9a). ^{1}H NMR (500 MHz, $CDCl_3$) δ: 8.10 (d, *J* = 8.4 Hz, 2H), 7.61 (dd, *J* = 7.4, 7.3 Hz, 1H), 7.46 (dd, *J* = 8.4, 7.5 Hz, 2H), 6.32 (s, 1H), 6.19 (t, *J* = 8.7 Hz, 1H), 5.61 (d, *J* = 7.1 Hz, 1H), 5.43 (d, *J* = 4.5 Hz, 1H), 5.11 (d, *J* = 4.7, 1H), 4.98 (d, *J* = 9.7, 1H), 4.62 (dd, *J* = 4.7, 4.5 Hz, 1H), 4.46 (dd , *J* = 9, 9 Hz, 1H), 4.30 (d, *J* = 9 Hz, 1H), 4.15 (d, *J* = 9 Hz, 1H), 3.85 (d, *J* = 7.1 Hz, 1H), 2.55 (m, 1H), 2.32 (q, *J* = 7.6 Hz, 2H), 2.28 (s, 3H), 2.24, (s, 3H), 2.11 (s, 3H), 2.06 (s, 3H), 1.87 (m, 1H), 1.76 (s, 3H), 1.66 (s, 3H), 1.13 (s, 6H) (Figure S18). ^{13}C NMR (126 MHz, CDCl3) δ: 204.18, 171.36, 170.66, 167.14, 165.78, 146.46, 140.82, 133.69, 131.75, 130.12, 129.31, 128.63, 118.76, 84.43, 80.73, 79.07, 77.81, 77.12, 76.23, 74.89, 72.32, 67.92, 58.68, 52.54, 46.12, 42.69, 38.57, 35.59, 26.95, 26.01, 22.59, 20.92, 18.24, 15.62, 8.43 (Figure S19). LC/ESI-MS monoisotopic exact mass *m/z* 728.3194 $[M + H]^{+}$; calculated for $C_{38}H_{50}NO_{13}$: 728.3282.

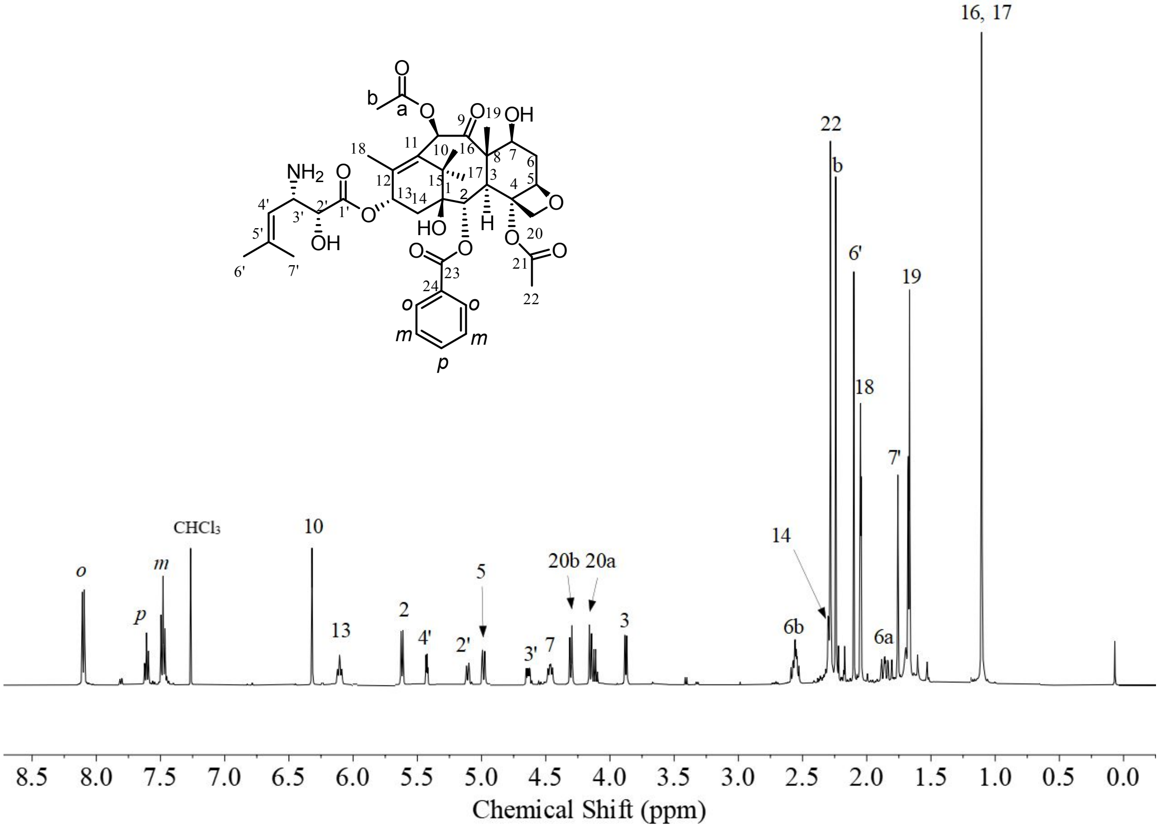

Figure S18. ^{1}H NMR of 3'-*N*-de(*tert*-butoxycarbonyl)-SB-T-1212.

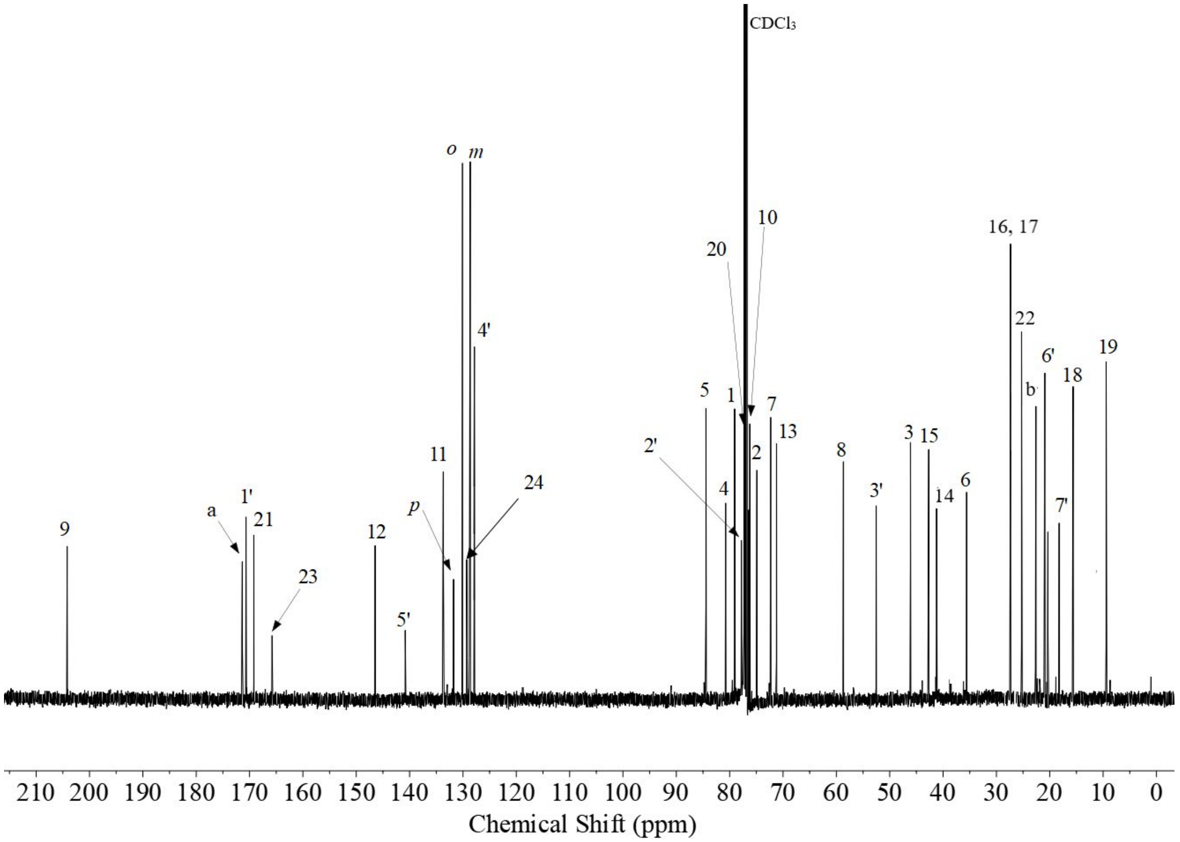

Figure S19. ^{13}C NMR of 3'-*N*-de(*tert*-butoxycarbonyl)-SB-T-1212.

NMR Data for 2-debenzoyl-2-(3-fluorobenzoyl)-3'-*N*-de(*tert*-butoxycarbonyl)-SB-T-1212 (9b). ^{1}H NMR (500 MHz, $CDCl_3$) δ: 8.11 (d, *J* = 7.4 Hz, 1H), 7.86 (s, 1H) 7.75 (d, *J* = 7.0 Hz, 1H), 7.49 (dd, *J* = 8.4, 7.5 Hz, 1H), 6.32 (s, 1H), 6.19 (t, *J* = 8.5 Hz, 1H), 5.62 (d, *J* = 7.1 Hz, 1H), 5.42 (d, *J* = 4.5 Hz, 1H), 5.13 (d, *J* = 4.6 Hz, 1H), 4.98 (d, *J* = 9.7 Hz, 1H), 4.64 (dd, *J* = 4.7, 4.5 Hz, 1H), 4.47 (*J* = 9, 9Hz, 1H), 4.30 (d, *J* = 9 Hz, 1H), 4.14 (d, *J* = 9 Hz, 1H), 3.89 (d, *J* = 7.1 Hz, 1H), 2.56 (m, 1H), 2.31 (q, *J* = 7.6 Hz, 2H), 2.29 (s, 3H), 2.25, (s, 3H), 2.11 (s, 3H), 2.05 (s, 3H), 1.87 (m, 1H), 1.76 (s, 3H), 1.67 (s, 3H), 1.11 (s, 6H) (Figure S20). ^{13}C NMR (126 MHz, $CDCl_3$) δ: 204.17, 171.38, 170.73, 168.08, 163.52, 146.44, 140.91, 133.71, 130.19, 129.28, 128.64, 125.91, 120.87, 118.67, 84.47, 80.76, 79.09, 77.73, 77.16, 76.24, 74.91, 72.32, 67.92, 58.68, 52.69, 46.13, 42.68, 38.55, 35.57, 26.96, 26.08, 22.64, 20.94, 18.25, 15.64, 8.46 (Figure S21). LC/ESI-MS monoisotopic exact mass *m/z* 746.3094 [M + H]$^+$; calculated for $C_{38}H_{49}FNO_{13}$: 746.3187.

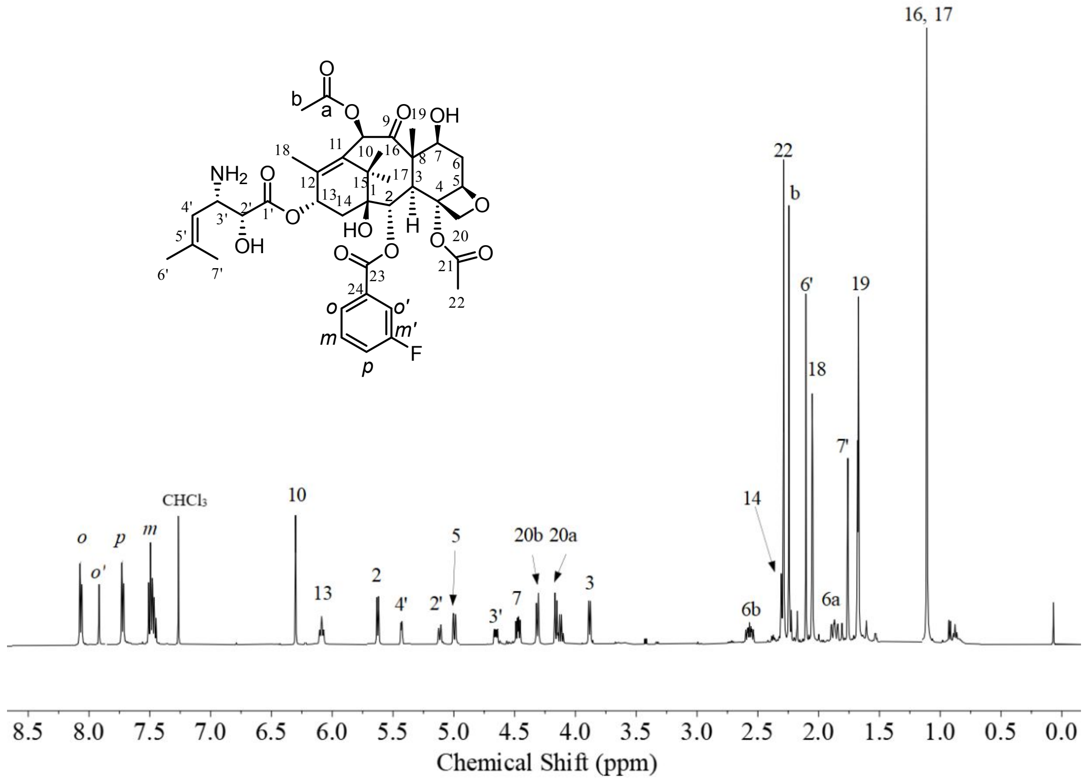

Figure S20. ^{1}H NMR of 2-debenzoyl-2-(3-F-benzoyl)-3'-*N*-de(*tert*-butoxycarbonyl)-SB-T-1212.

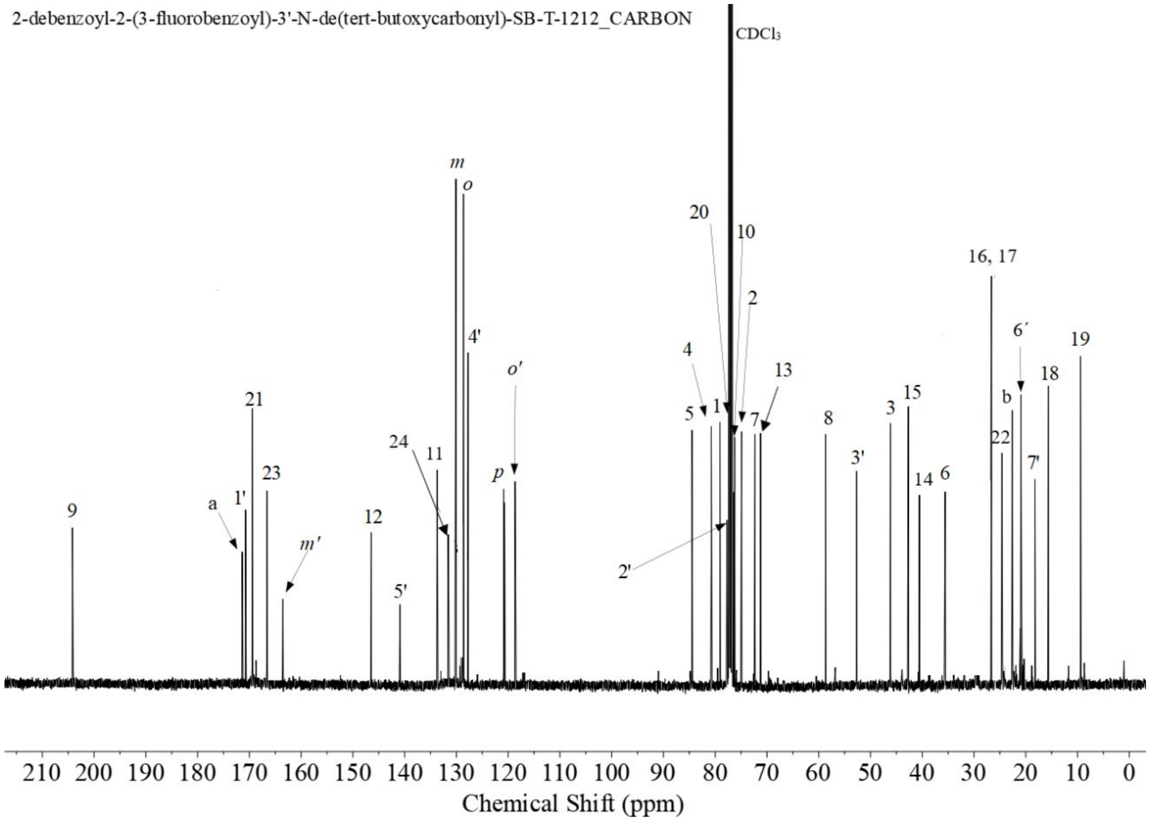

Figure S21. ^{13}C NMR of 2-debenzoyl-2-(3-F-benzoyl)-3'-*N*-de(*tert*-butoxycarbonyl)-SB-T-1212.

NMR Data for 2-debenzoyl-2-(3-chlorobenzoyl)-3'-*N*-de(*tert*-butoxycarbonyl)-SB-T-1212 (9c). ^{1}H NMR (500 MHz, $CDCl_3$) δ: 8.12 (s, 1H), 7.89 (d, *J* = 7.4 Hz, 1H), 7.77 (d, *J* = 7.2 Hz, 1H), 7.49 (dd, *J* = 8.5, 7.4 Hz, 1H), 6.32 (s, 1H), 6.19 (t, *J* = 8.5 Hz, 1H), 5.62 (d, *J* = 7.1 Hz, 1H), 5.42 (d, J = 4.5 Hz, 1H), 5.13 (d, J = 4.6 Hz, 1H), 4.98 (d, *J* = 9.7 Hz, 1H), 4.64 (dd, *J* = 4.7, 4.5 Hz, 1H), 4.47 (*J* = 9, 9Hz, 1H), 4.30 (d, *J* = 9 Hz, 1H), 4.14 (d, *J* = 9 Hz, 1H), 3.89 (d, *J* = 7.1 Hz, 1H), 2.56 (m, 1H), 2.31 (q, *J* = 7.6 Hz, 2H), 2.29 (s, 3H), 2.25, (s, 3H), 2.11 (s, 3H), 2.05 (s, 3H), 1.87 (m, 1H), 1.76 (s, 3H), 1.67 (s, 3H), 1.11 (s, 6H) (Figure S22). ^{13}C NMR (126 MHz, CDCl3) δ: 204.15, 171.35, 170.72, 168.09, 165.12, 146.43, 140.91, 135.12, 134.28, 131.62, 130.28, 129.82, 129.76, 127.65, 126.84, 84.47, 80.76, 79.09, 77.73, 77.16, 76.24, 74.91, 72.32, 71.92, 58.68, 53.69, 46.13, 42.68, 39.55, 35.57, 26.96, 24.08, 21.64, 20.94, 17.25, 15.64, 8.44 (Figure S23). LC/ESI-MS monoisotopic exact mass *m/z* 762.2786 [M + H]$^+$; calculated for $C_{38}H_{49}FNO_{13}$: 762.2892.

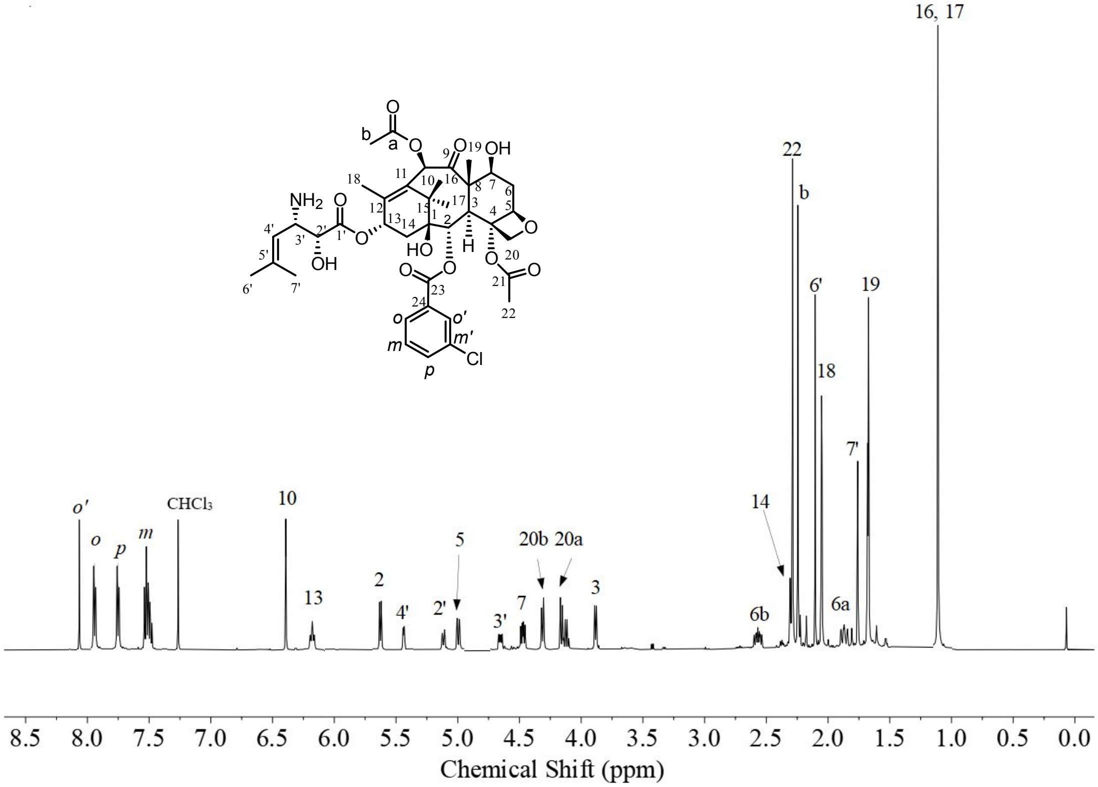

Figure S22. ^{1}H NMR of 2-debenzoyl-2-(3-Cl-benzoyl)-3'-*N*-de(*tert*-butoxycarbonyl)-SB-T-1212.

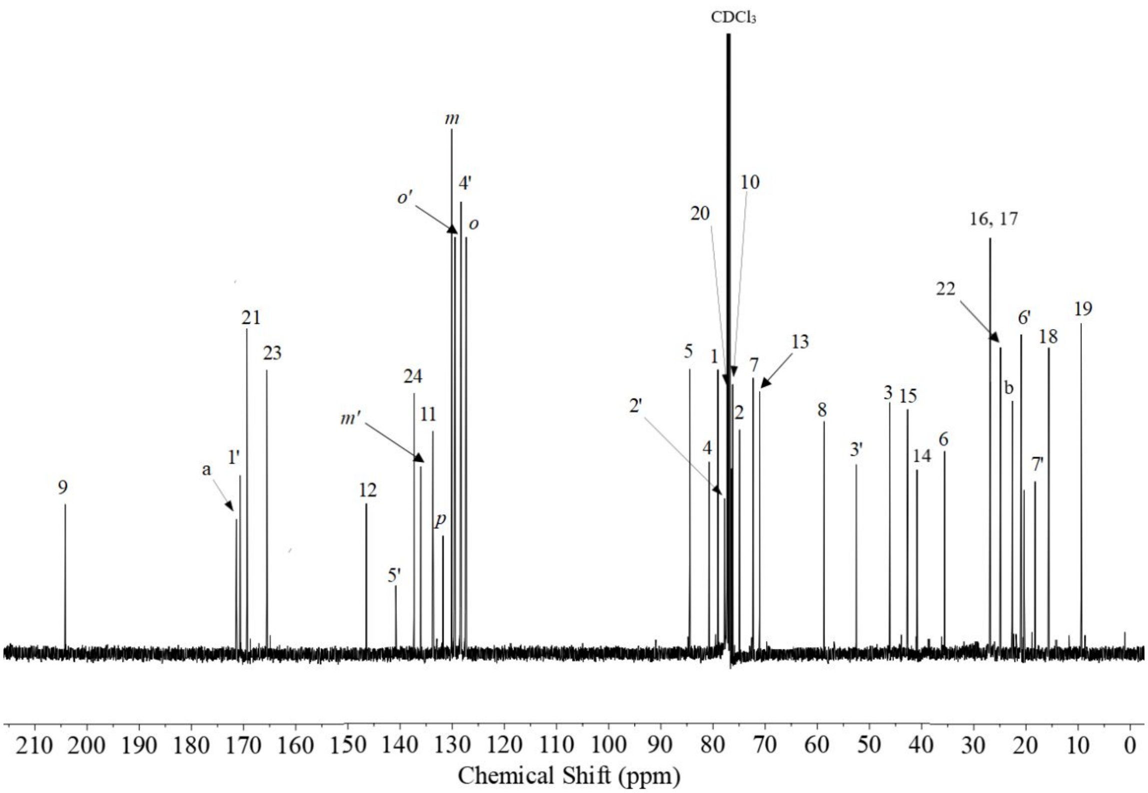

Figure S23. ^{13}C NMR of 2-debenzoyl-2-(3-Cl-benzoyl)-3'-*N*-de(*tert*-butoxycarbonyl)-SB-T-1212.

NMR Data for 2-debenzoyl-2-(3-methoxybenzoyl)-3'-*N*-de(*tert*-butoxycarbonyl)-SB-T-1212 (9d). ^{1}H NMR (500 MHz, $CDCl_3$) δ: 8.09 (d, *J* = 7.4 Hz, 1H), 7.67 (s, 1H) 7.59 (d, *J* = 7.0 Hz, 1H), 7.47 (dd, *J* = 8.4, 7.5 Hz, 1H), 6.32 (s, 1H), 6.19 (t, *J* = 8.5 Hz, 1H), 5.62 (d, *J* = 7.1 Hz, 1H), 5.42 (d, *J* = 4.5 Hz, 1H), 5.13 (d, *J* = 4.6 Hz, 1H), 4.98 (d, *J* = 9.7 Hz, 1H), 4.64 (dd, *J* = 4.7, 4.5 Hz, 1H), 4.47 (*J* = 9, 9Hz, 1H), 4.30 (d, *J* = 9 Hz, 1H), 4.14 (d, *J* = 9 Hz, 1H), 3.89 (d, *J* = 7.1 Hz, 1H), 3.86 (s, 3H), 2.56 (m, 1H), 2.31 (q, *J* = 7.6 Hz, 2H), 2.29 (s, 3H), 2.25, (s, 3H), 2.11 (s, 3H), 2.05 (s, 3H), 1.87 (m, 1H), 1.76 (s, 3H), 1.67 (s, 3H), 1.11 (s, 6H) (Figure S24). ^{13}C NMR (126 MHz, CDCl3) δ: 203.42, 171.35, 170.71, 168.16, 165.14, 158.62, 146.47, 140.93, 131.62, 130.42, 129.76, 128.64, 121.57, 117.84, 114. 63, 84.47, 80.76, 79.09, 77.73, 77.16, 76.24, 74.91, 72.32, 71.92, 58.68, 55.8, 53.69, 46.13, 42.68, 39.55, 35.57, 26.96, 24.08, 21.64, 20.94, 17.25, 15.64, 8.44 (Figure S25).LC/ESI-MS monoisotopic exact mass *m/z* 758.3293 [M + H] $^{+}$; calculated for $C_{39}H_{52}NO_{14}$: 758.3387.

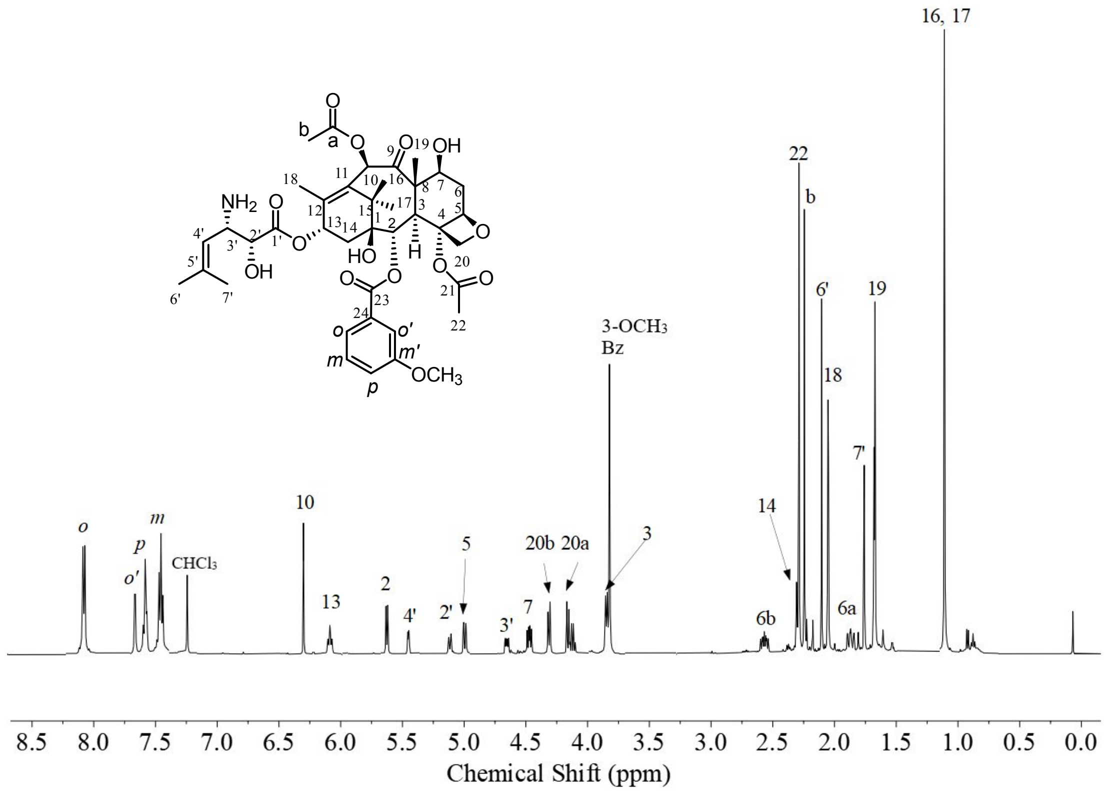

Figure S24. ^{1}H NMR of 2-debenzoyl-2-(3-OCH$_3$-benzoyl)-3'-*N*-de(*tert*-butoxycarbonyl)-SB-T-1212.

2-debenzoyl-2-(3-methoxybenzoyl)-3'-N-de(tert-butoxycarbonyl)-SB-T-1212_CARBON

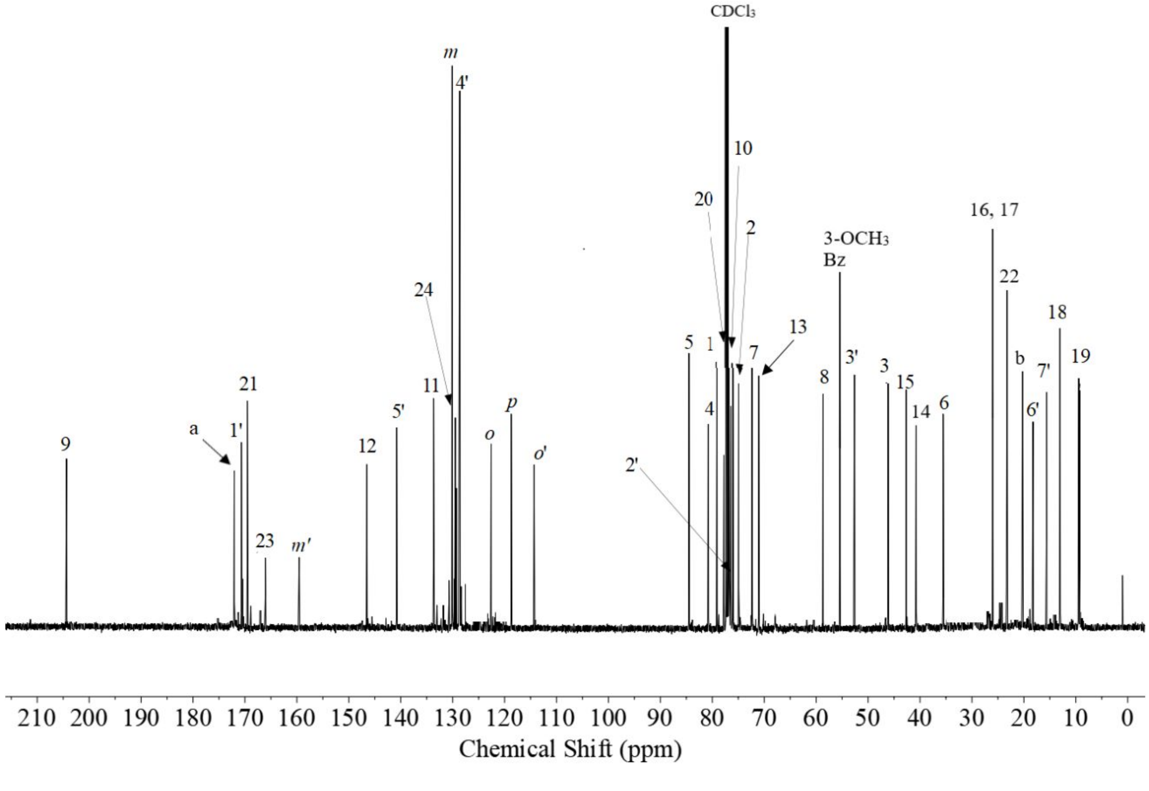

Figure S25. ^{13}C NMR of 2-debenzoyl-2-(3-OCH_3-benzoyl)-3'-*N*-de(*tert*-butoxycarbonyl)-SB-T-212.

NMR Data for 2-debenzoyl-2-(3-difluoromethoxybenzoyl)-3'-*N*-de(*tert*-butoxycarbonyl)-SB-T-1212 (9e). ^{1}H NMR (500 MHz, $CDCl_3$) δ: 8.12 (d, *J* = 7.4 Hz, 1H), 7.85 (s, 1H), 7.71 (d, *J* = 7.1Hz, 1H), 7.64 (s, 1H), 7.48 (dd, *J* = 8.4, 7.5 Hz, 1H), 6.32 (s, 1H), 6.19 (t, *J* = 8.5 Hz, 1H), 5.62 (d, *J* = 7.1 Hz, 1H), 5.42 (d, *J* = 4.5 Hz, 1H), 5.13 (d, *J* = 4.6 Hz, 1H), 4.98 (d, *J* = 9.7 Hz, 1H), 4.64 (dd, *J* = 4.7, 4.5 Hz, 1H), 4.47 (*J* = 9, 9Hz, 1H), 4.30 (d, *J* = 9 Hz, 1H), 4.14 (d, *J* = 9 Hz, 1H), 3.89 (d, *J* = 7.1 Hz, 1H), 2.56 (m, 1H), 2.31 (q, *J* = 7.6 Hz, 2H), 2.29 (s, 3H), 2.25, (s, 3H), 2.12 (s, 3H), 2.06 (s, 3H), 1.88 (m, 1H), 1.76 (s, 3H), 1.68 (s, 3H), 1.12 (s, 6H) (Figure S26). ^{13}C NMR (126 MHz, $CDCl_3$) δ: 205.28, 171.42, 170.65, 169.32, 167.46, 164.81, 147.35, 141.23, 132.82, 131.36, 129.52, 128.41, 127.91, 118.65, 116.72, 84.47, 80.76, 79.09, 77.73, 77.16, 76.24, 74.91, 72.32, 67.92, 58.68, 52.69, 46.13, 42.68, 38.55, 35.57, 26.96, 26.08, 22.64, 20.94, 18.25, 15.64, 8.47 (Figure S27). LC/ESI-MS monoisotopic exact mass *m/z* 794.3096 $[M + H]^+$; calculated for $C_{39}H_{50}F_2NO_{14}$: 794.3199.

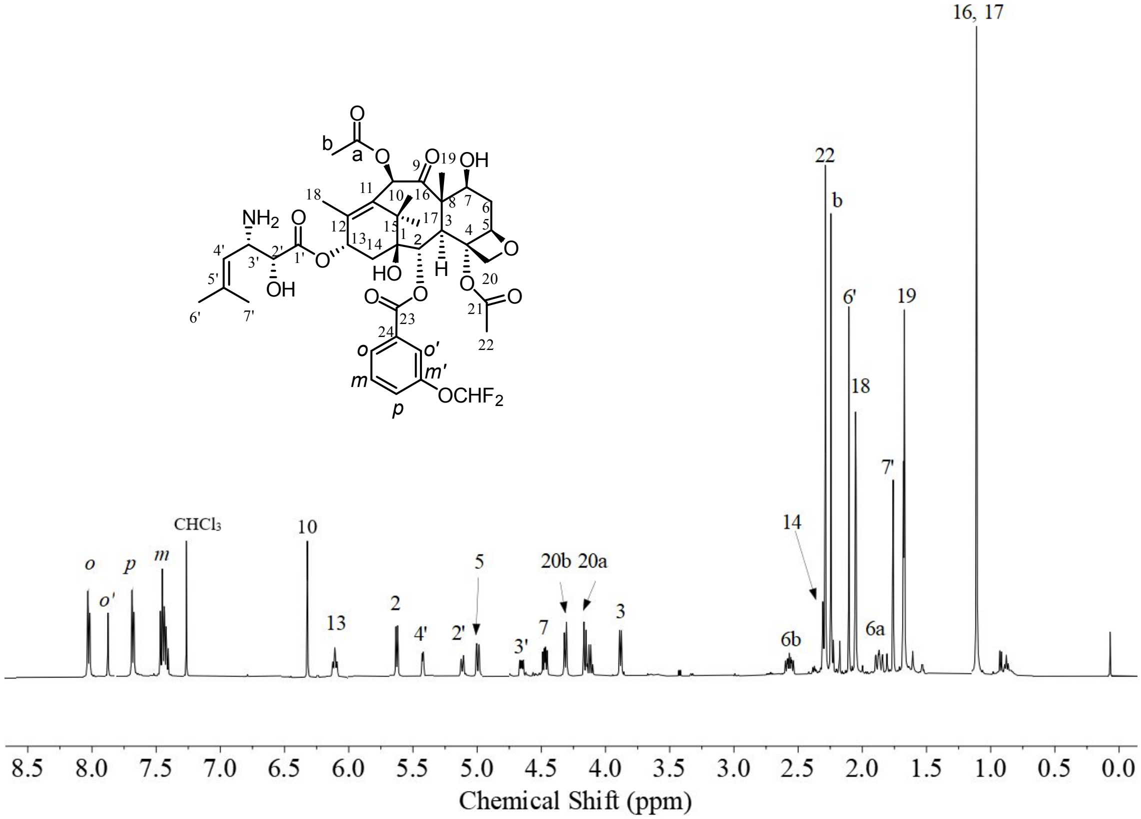

Figure S26. ^{1}H NMR of 2-debenzoyl-2-(3-$OCHF_2$-benzoyl)-3'-*N*-de(*tert*-butoxycarbonyl)-SB-T-1212.

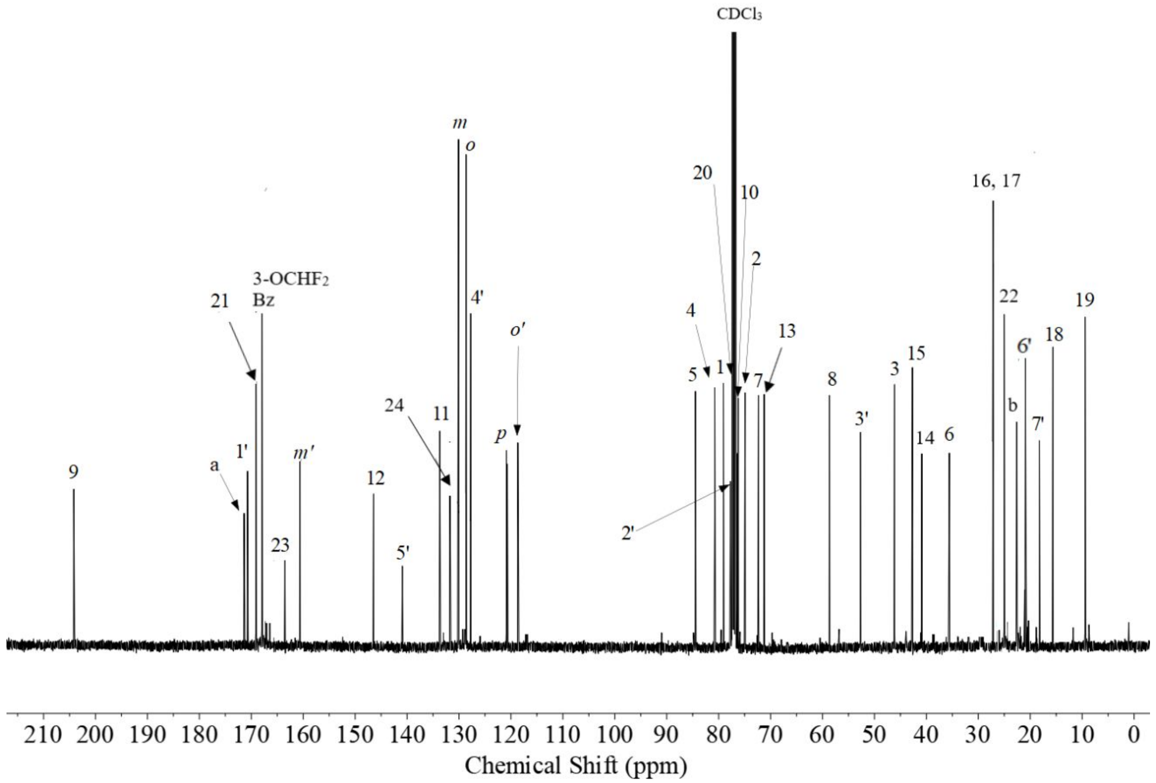

Figure S27. ^{13}C NMR of 2-debenzoyl-2-(3-OCHF$_2$-benzoyl)-3'-*N*-de(*tert*-butoxycarbonyl)-SB-T-1212.

NMR Data for 2-debenzoyl-2-(3-trifluoromethoxybenzoyl)-3'-*N*-de(*tert*-butoxycarbonyl)-SB-T-1212 (9f). ^{1}H NMR (500 MHz, $CDCl_3$) δ: 8.09 (d, *J* = 7.5 Hz, 1H), 7.87 (s, 1H) 7.68 (d, *J* = 7.2 Hz, 1H), 7.45 (dd, *J* = 8.2, 7.8 Hz, 1H), 6.32 (s, 1H), 6.19 (t, *J* = 8.4 Hz, 1H), 5.63 (d, *J* = 7.1 Hz, 1H), 5.42 (d, *J* = 4.5 Hz, 1H), 5.13 (d, *J* = 4.6 Hz, 1H), 4.98 (d, *J* = 9.7 Hz, 1H), 4.64 (dd, *J* = 4.7, 4.5 Hz, 1H), 4.47 (*J* = 9, 9Hz, 1H), 4.30 (d, *J* = 9 Hz, 1H), 4.14 (d, *J* = 9 Hz, 1H), 3.89 (d, *J* = 7.1 Hz, 1H), 2.56 (m, 1H), 2.31 (q, *J* = 7.6 Hz, 2H), 2.29 (s, 3H), 2.25, (s, 3H), 2.12 (s, 3H), 2.06 (s, 3H), 1.87 (m, 1H), 1.78 (s, 3H), 1.66 (s, 3H), 1.12(s, 6H) (Figure S28). ^{13}C NMR (126 MHz, $CDCl_3$) δ: 204.27, 171.31, 170.82, 169.12, 167.35, 165.11, 146.62, 141.11, 132.18, 130.47, 129.12, 127.75, 127.72, 126.36, 121.68, 117.55, 84.47, 80.76, 79.09, 77.73, 77.16, 76.24, 74.91, 72.32, 71.92, 58.68, 53.69, 46.13, 42.68, 39.55, 35.57, 26.96, 24.08, 21.64, 20.94, 17.25, 15.64, 8.44 (Figure S29). LC/ESI-MS monoisotopic exact mass *m/z* 812.3085 [M + H]$^+$; calculated for $C_{39}H_{49}F_2NO_{14}$: 812.3105.

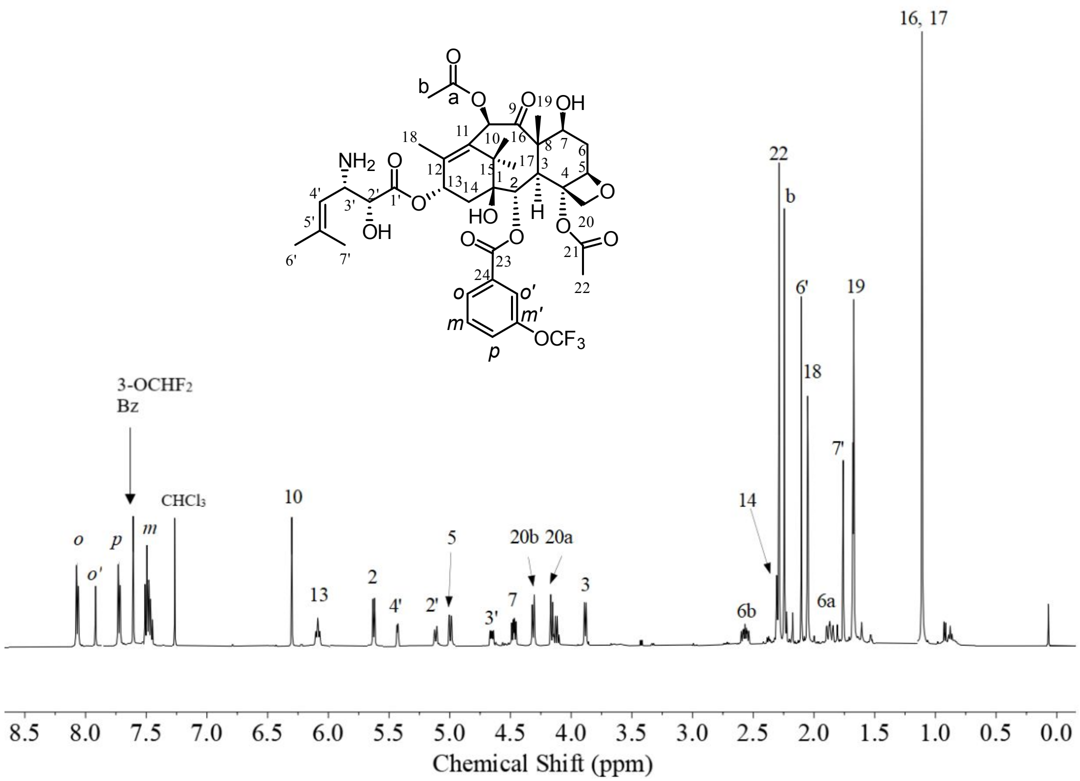

Figure S28. ^{1}H NMR of 2-debenzoyl-2-(3-OCF$_3$-benzoyl)-3'-*N*-de(*tert*-butoxycarbonyl)-SB-T-1212.

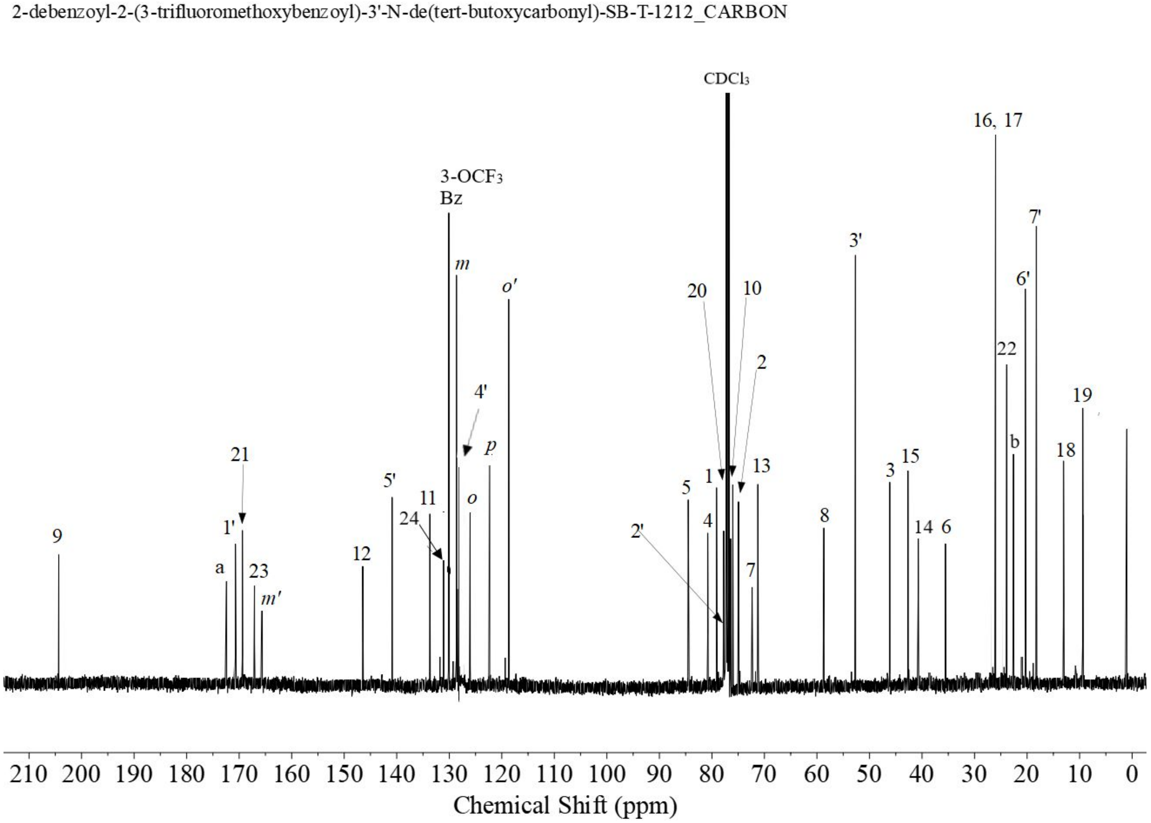

Figure S29. ^{13}C NMR of 2-debenzoyl-2-(3-OCF$_3$-benzoyl)-3'-*N*-de(*tert*-butoxycarbonyl)-SB-T-1212.

NMR Data for 3′-*N*-de(*tert*-butoxycarbonyl)-SB-T-1213 (10a). ^{1}H NMR (500 MHz, $CDCl_3$) δ: 8.08 (d, *J* = 8.4 Hz, 2H), 7.62 (dd, *J* = 7.4, 7.3 Hz, 1H), 7.46 (dd, *J* = 8.4, 7.5 Hz, 2H), 6.31 (s, 1H), 6.19 (t, *J* = 8.7 Hz, 1H), 5.61 (d, *J* = 7.1 Hz, 1H), 5.42 (d, *J* = 4.5 Hz, 1H), 5.11 (d, *J* = 4.7, 1H), 4.98 (d, *J* = 9.7, 1H), 4.62 (dd, *J* = 4.7, 4.5 Hz, 1H), 4.46 (dd , *J* = 9, 9 Hz, 1H), 4.30 (d, *J* = 9 Hz, 1H), 4.15 (d, *J* = 9 Hz, 1H), 3.85 (d, *J* = 7.1 Hz, 1H), 2.54 (m, 1H), 2.53 (q, *J* = 8.2 Hz, 2H), 2.30 (q, *J* = 7.6 Hz, 2H), 2.28 (s, 3H), 2.09 (s, 3H), 2.03 (s, 3H), 1.85 (m, 1H), 1.75 (s, 3H), 1.64 (s, 3H), 1.23 (t, *J* = 7.6 Hz, 3H), 1.09 (s, 6H) Figure S30). ^{13}C NMR (126 MHz, CDCl3) δ: 204.28, 174.68, 172.85, 168.23, 166.89, 147.45, 142.78, 134.66, 132.76, 130.98, 129.32, 128.64, 118.76, 84.46, 81.75, 79.65, 77.68, 76.53, 76.14, 74.91, 72.43, 67.85, 58.66, 53.69, 46.26, 42.76, 38.96, 35.62, 26.92, 22.58, 20.73, 18.95, 15.91, 8.45, 8.25(Figure S31). LC/ESI-MS monoisotopic exact mass *m/z* 742.3378 $[M + H]^+$; calculated for $C_{39}H_{52}NO_{13}$: 742.3439.

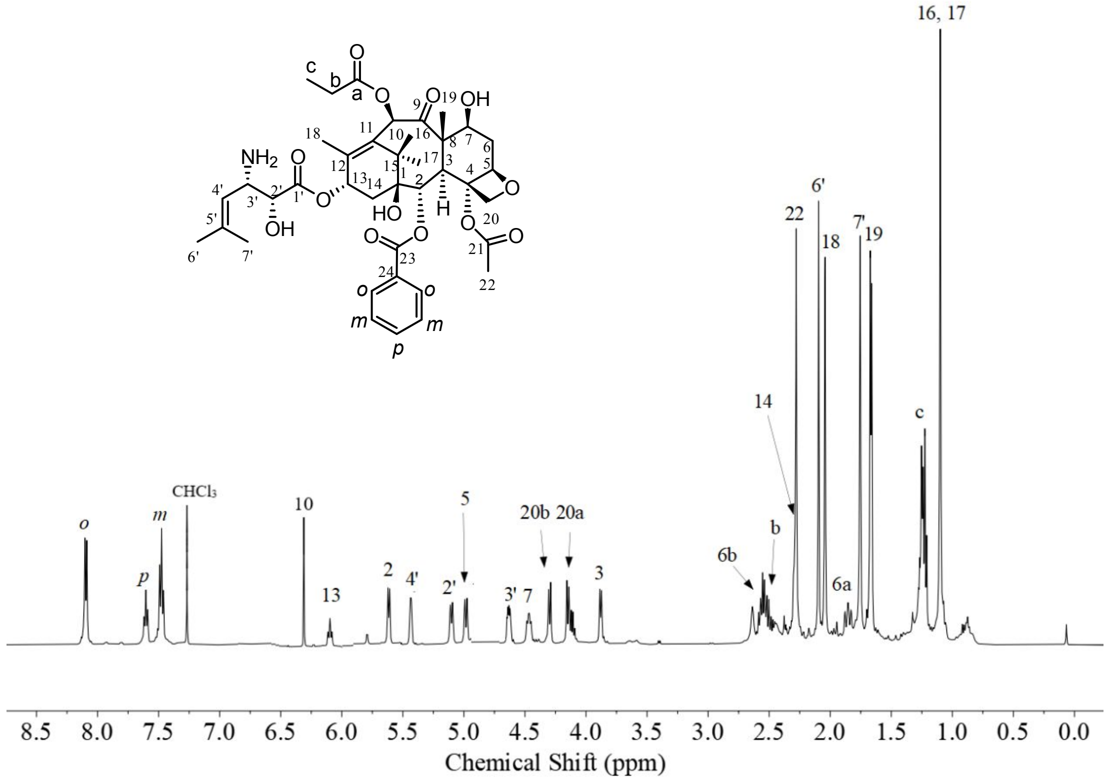

Figure S30. ^{1}H NMR of 3'-*N*-de(*tert*-butoxycarbonyl)-SB-T-1213.

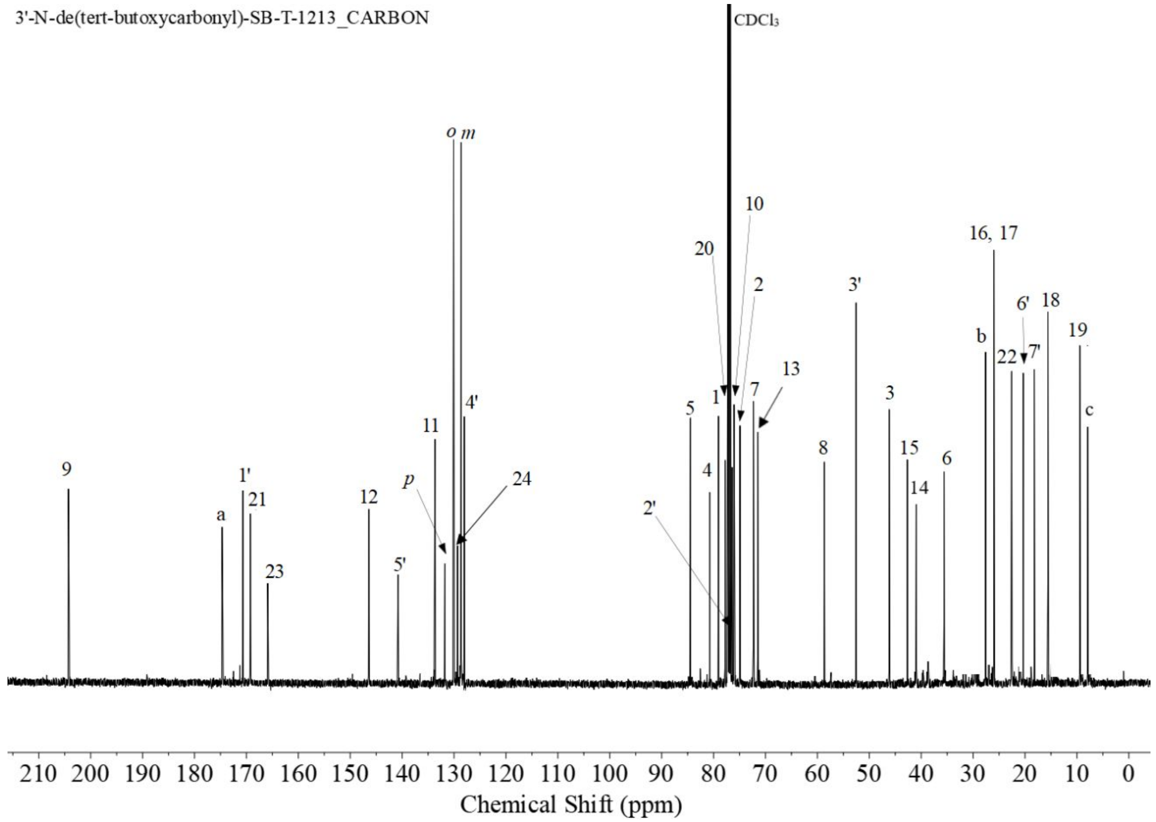

Figure S31. ^{13}C NMR of 3'-*N*-de(*tert*-butoxycarbonyl)-SB-T-1213.

NMR Data for 2-debenzoyl-2-(3-fluorobenzoyl)-3'-*N*-de(*tert*-butoxycarbonyl)-SB-T-1213. (10b). ^{1}H NMR (500 MHz, $CDCl_3$) δ: 8.11 (d, J = 7.4 Hz, 1H), 7.86 (s, 1H) 7.75 (d, J = 7.0 Hz, 1H), 7.49 (dd, J = 8.4, 7.5 Hz, 1H), 6.32 (s, 1H), 6.18 (t, J = 8.5 Hz, 1H), 5.62 (d, J = 7.1 Hz, 1H), 5.42 (d, J = 4.5 Hz, 1H), 5.13 (d, J = 4.6 Hz, 1H), 4.98 (d, J = 9.7 Hz, 1H), 4.64 (dd, J = 4.7, 4.5 Hz, 1H), 4.47 (J = 9, 9Hz, 1H), 4.30 (d, J = 9 Hz, 1H), 4.14 (d, J = 9 Hz, 1H), 3.89 (d, J = 7.1 Hz, 1H), 2.56 (m, 1H), 2.54 (q, J = 8.2 Hz, 2H), 2.31 (q, J = 7.6 Hz, 2H), 2.29 (s, 3H), 2.10 (s, 3H), 2.05 (s, 3H), 1.87 (m, 1H), 1.77 (s, 3H), 1.67 (s, 3H), 1.25 (t, J = 7.6 Hz, 3H), 1.11 (s, 6H) (Figure S32). ^{13}C NMR (126 MHz, CDCl3) δ: 204.26, 174.71, 171,98, 168.91, 167.25, 163.52, 147.56, 142.92, 134.88, 130.46, 129.96, 128.64, 125.85, 120.78, 118.67, 84.89, 81.78, 79.82, 77.85, 76.92, 76.34, 74.95, 72.46, 67.89, 58.78, 63.92, 46.67, 42.82, 39.14, 35.91, 27.46, 67.89, 58.78, 53.92, 46.67, 42.82, 39.14, 35.91, 27.95, 27.17, 22.92, 20.81, 19.18, 16.21, 8.55, 8.37 (Figure S33). LC/ESI-MS monoisotopic exact mass *m/z* 760.3284 [M + H] $^{+}$; calculated for $C_{39}H_{51}FNO_{13}$: 760.3345.

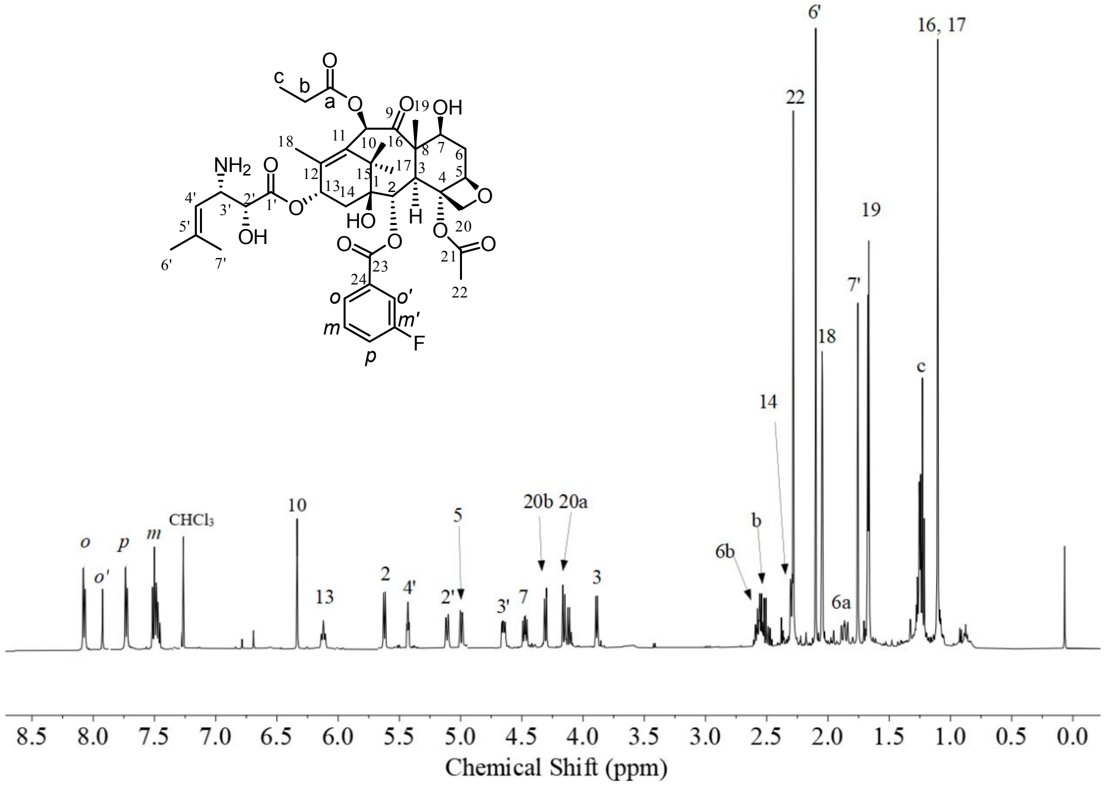

Figure S32. ^{1}H NMR of 2-debenzoyl-2-(3-F-benzoyl)-3'-*N*-de(*tert*-butoxycarbonyl)-SB-T-1213.

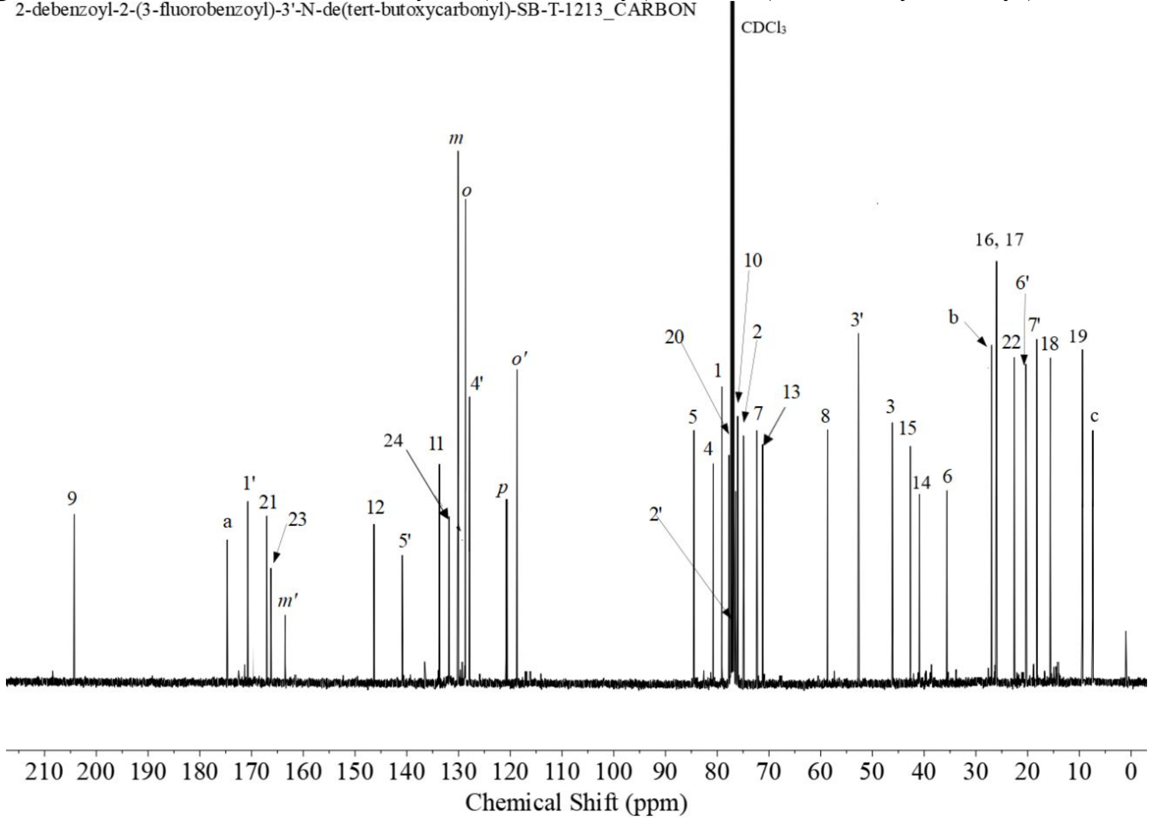

Figure S33. ^{13}C NMR of 2-debenzoyl-2-(3-F-benzoyl)-3'-*N*-de(*tert*-butoxycarbonyl)-SB-T-1213.

NMR Data for 2-debenzoyl-2-(3-chlorobenzoyl)-3'-*N*-de(*tert*-butoxycarbonyl)-SB-T-1213 (10c). ^{1}H NMR (500 MHz, $CDCl_3$) δ: 8.12 (s, 1H), 7.89 (d, *J* = 7.4 Hz, 1H), 7.77 (d, *J* = 7.2 Hz, 1H), 7.49 (dd, *J* = 8.5, 7.4 Hz, 1H), 6.32 (s, 1H), 6.19 (t, *J* = 8.5 Hz, 1H), 5.62 (d, *J* = 7.1 Hz, 1H), 5.42 (d, J = 4.5 Hz, 1H), 5.13 (d, J = 4.6 Hz, 1H), 4.98 (d, *J* = 9.7 Hz, 1H), 4.64 (dd, *J* = 4.7, 4.5 Hz, 1H), 4.47 (*J* = 9, 9Hz, 1H), 4.30 (d, *J* = 9 Hz, 1H), 4.14 (d, *J* = 9 Hz, 1H), 3.89 (d, *J* = 7.1 Hz, 1H), 2.56 (m, 1H), 2.54 (q, *J* = 8.2 Hz, 2H), 2.31 (q, *J* = 7.6 Hz, 2H), 2.29 (s, 3H), 2.10 (s, 3H), 2.05 (s, 3H), 1.87 (m, 1H), 1.77 (s, 3H), 1.67 (s, 3H), 1.25 (t, *J* = 7.6 Hz, 3H), 1.11 (s, 6H) (Figure S34). ^{13}C NMR (126 MHz, $CDCl_3$) δ: 205.26, 174.71, 171,98, 168.91, 167.25, 146.43, 140.91, 135.12, 134.28, 131.62, 130.28, 129.82, 129.76, 127.65, 126.84, 84.89, 81.78, 79.82, 77.85, 76.92, 76.34, 74.95, 72.46, 67.89, 58.78, 63.92, 46.67, 42.82, 39.14, 35.91, 27.46, 67.89, 58.78, 53.92, 46.67, 42.82, 39.14, 35.91, 27.95, 27.17, 22.92, 20.81, 19.18, 16.21, 8.55, 8.37 (Figure S35). LC/ESI-MS monoisotopic exact mass *m/z* 776.2969 [M + H] $^{+}$; calculated for $C_{39}H_{51}ClNO_{13}$: 776.3049.

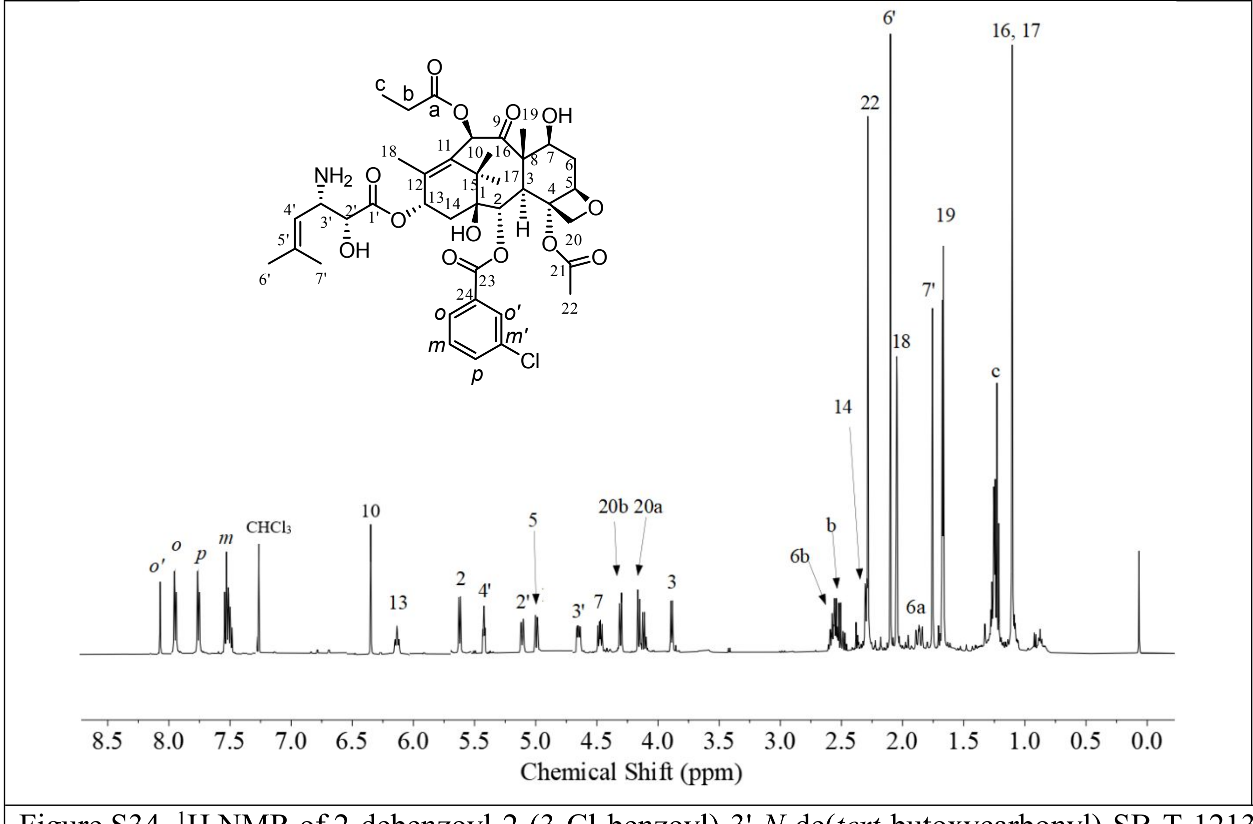

Figure S34. ^{1}H NMR of 2-debenzoyl-2-(3-Cl-benzoyl)-3'-*N*-de(*tert*-butoxycarbonyl)-SB-T-1213.

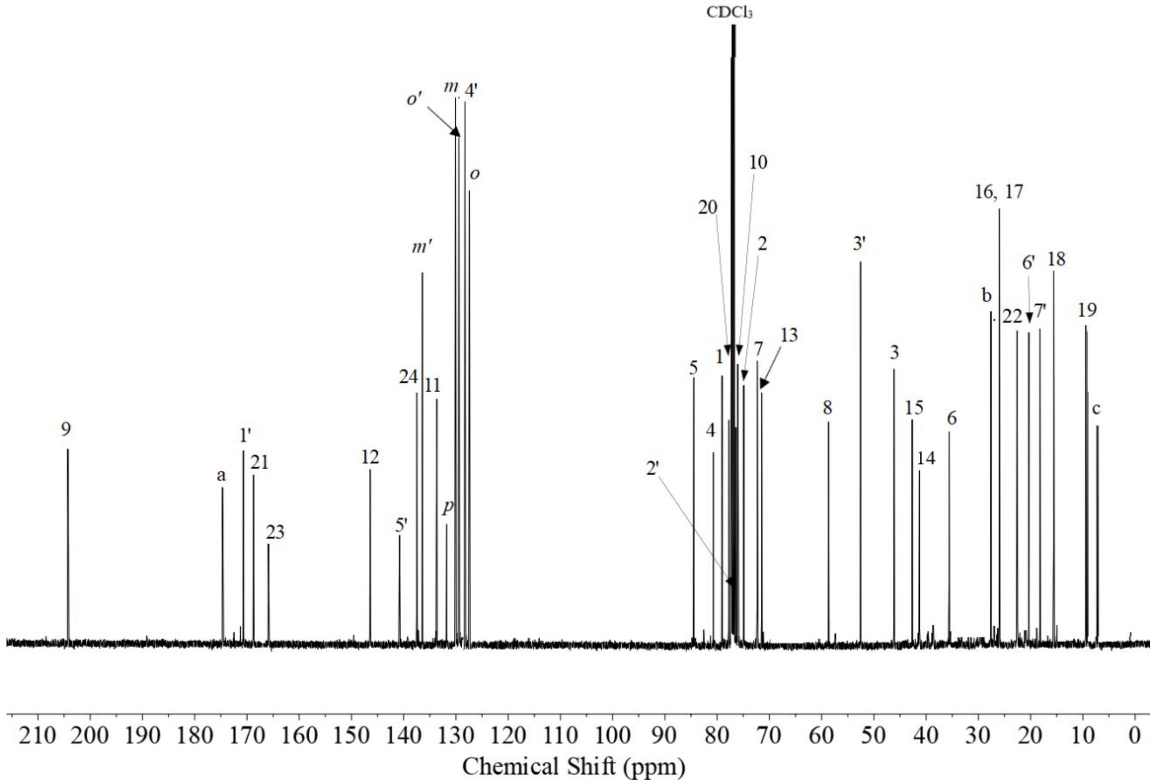

Figure S35. ^{13}C NMR of 2-debenzoyl-2-(3-Cl-benzoyl)-3'-*N*-de(*tert*-butoxycarbonyl)-SB-T-1213.

2-Debenzoyl-2-(3-methoxybenzoyl)-3'-*N*-de(*tert*-butoxycarbonyl)-SB-T-1213 (10d). ^{1}H NMR (500 MHz, $CDCl_3$) δ: 8.08 (d, *J* = 7.4 Hz, 1H), 7.65 (s, 1H) 7.56 (d, *J* = 7.0 Hz, 1H), 7.47 (dd, *J* = 8.4, 7.5 Hz, 1H), 6.32 (s, 1H), 6.18 (t, *J* = 8.5 Hz, 1H), 5.62 (d, *J* = 7.1 Hz, 1H), 5.42 (d, *J* = 4.5 Hz, 1H), 5.13 (d, *J* = 4.6 Hz, 1H), 4.98 (d, *J* = 9.7 Hz, 1H), 4.64 (dd, *J* = 4.7, 4.5 Hz, 1H), 4.47 (*J* = 9, 9Hz, 1H), 4.30 (d, *J* = 9 Hz, 1H), 4.14 (d, *J* = 9 Hz, 1H), 3.89 (d, *J* = 7.1 Hz, 1H), 3.86 (s, 3H), 2.54 (m, 1H), 2.52 (q, *J* = 8.2 Hz, 2H), 2.30 (q, *J* = 7.6 Hz, 2H), 2.27 (s, 3H), 2.10 (s, 3H), 2.04 (s, 3H), 1.86 (m, 1H), 1.76 (s, 3H), 1.66 (s, 3H), 1.23 (t, *J* = 7.6 Hz, 3H), 1.10 (s, 6H) (Figure S36). ^{13}C NMR (126 MHz, CDCl3) δ: 204.26, 174.72, 171.96, 168.78, 166.78, 159.56, 147.33, 141.52, 133.67, 130.49, 129.49, 128.63, 122.59, 120.27, 114.32, 84.78, 81.76, 79.53, 77.84, 76.91, 76.32, 74.91, 72.33, 67.86, 58.67, 55.45, 52.64, 46.62, 42.78, 38.86, 35.84, 27.92, 26.95, 22.89, 20.79, 18.93, 15.96, 8.67, 8.48 (Figure S37). LC/ESI-MS monoisotopic exact mass *m/z* 772.3482 $[M + H]^+$; calculated for $C_{40}H_{54}NO_{14}$: 772.3544.

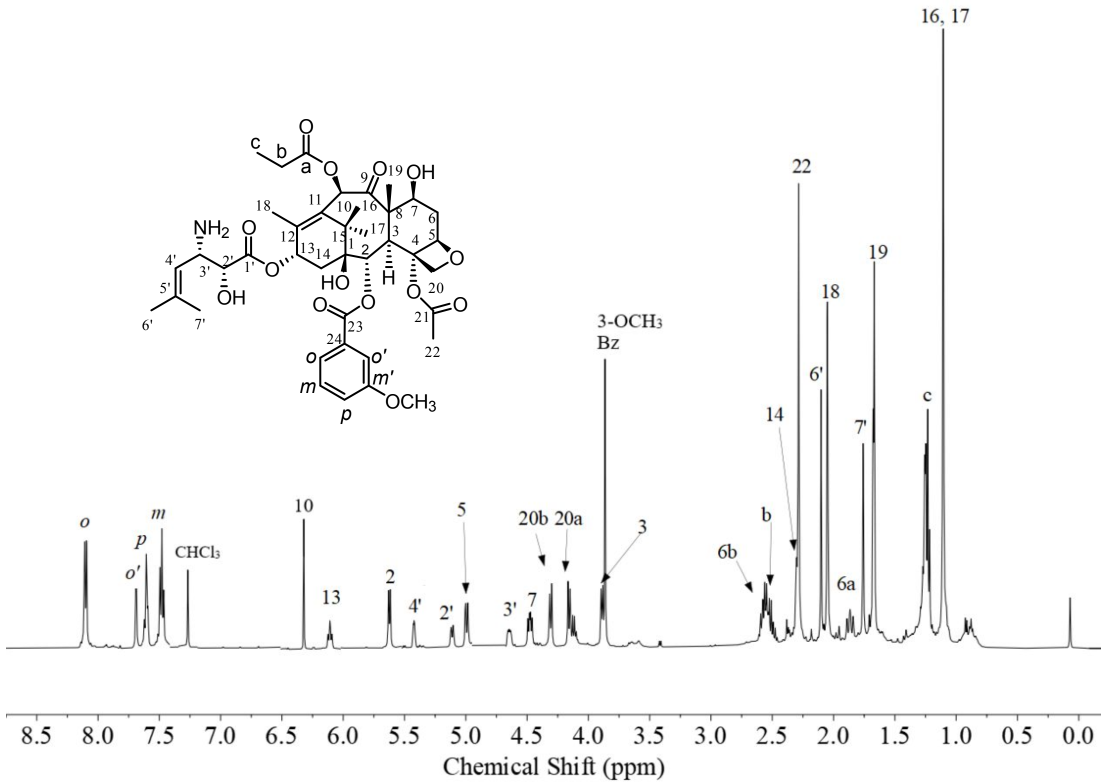

Figure S36. ^{1}H NMR of 2-debenzoyl-2-(3-OCH_3-benzoyl)-3'-*N*-de(*tert*-butoxycarbonyl)-SB-T-1213.

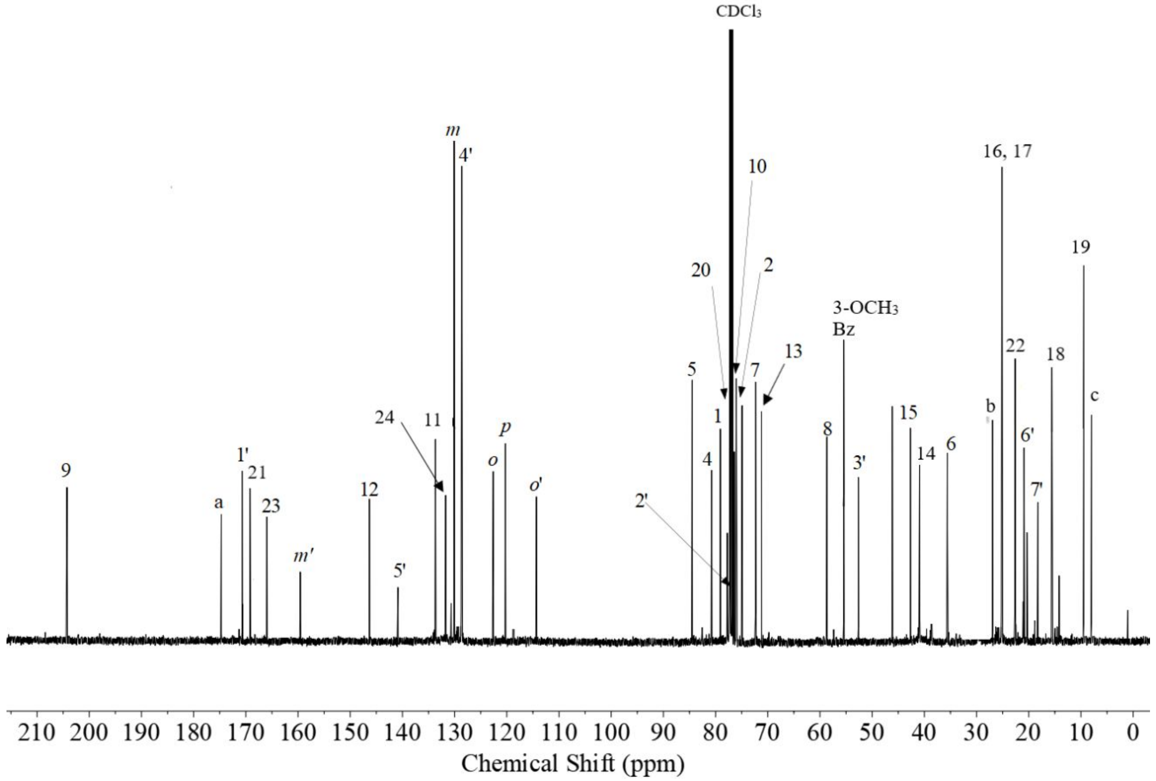

Figure S37. ^{13}C NMR of 2-debenzoyl-2-(3-OCH_3-benzoyl)-3'-*N*-de(*tert*-butoxycarbonyl)-SB-T-1213.

2-Debenzoyl-2-(3-difluoromethylbenzoyl)-3'-*N*-de(*tert*-butoxycarbonyl)-SB-T-1213 (10e). ^{1}H NMR (500 MHz, $CDCl_3$) δ: 8.12 (d, *J* = 7.4 Hz, 1H), 7.85 (s, 1H) 7.71 (d, *J* = 7.1Hz, 1H), 7.64 (s, 1H), 7.48 (dd, *J* = 8.4, 7.5 Hz, 1H), 6.32 (s, 1H), 6.19 (t, *J* = 8.5 Hz, 1H), 5.62 (d, *J* = 7.1 Hz, 1H), 5.42 (d, *J* = 4.5 Hz, 1H), 5.13 (d, *J* = 4.6 Hz, 1H), 4.98 (d, *J* = 9.7 Hz, 1H), 4.64 (dd, *J* = 4.7, 4.5 Hz, 1H), 4.47 (*J* = 9, 9Hz, 1H), 4.30 (d, *J* = 9 Hz, 1H), 4.14 (d, *J* = 9 Hz, 1H), 3.89 (d, *J* = 7.1 Hz, 1H), 2.54 (m, 1H), 2.52 (q, *J* = 8.2 Hz, 2H), 2.30 (q, *J* = 7.6 Hz, 2H), 2.27 (s, 3H), 2.10 (s, 3H), 2.04 (s, 3H), 1.86 (m, 1H), 1.76 (s, 3H), 1.66 (s, 3H), 1.23 (t, *J* = 7.6 Hz, 3H), 1.11 (s, 6H) (Figure S38). ^{13}C NMR (126 MHz, CDCl3) δ: 206.28, 171.45, 170.65, 169.32, 167.46, 164.81, 147.35, 141.23, 132.82, 131.36, 129.52, 128.41, 127.91, 118.65, 116.72, 84.46, 81.75, 79.65, 77.68, 76.53, 76.14, 74.91, 72.43, 67.85, 58.68, 53.69, 46.27, 42.76, 38.96, 35.62, 26.93, 22.56, 20.73, 18.95, 15.92, 8.46, 8.27 (Figure S39). LC/ESI-MS monoisotopic exact mass *m/z* 808.3298 [M + H] $^+$; calculated for $C_{40}H_{52}F_2NO_{14}$: 808.3356.

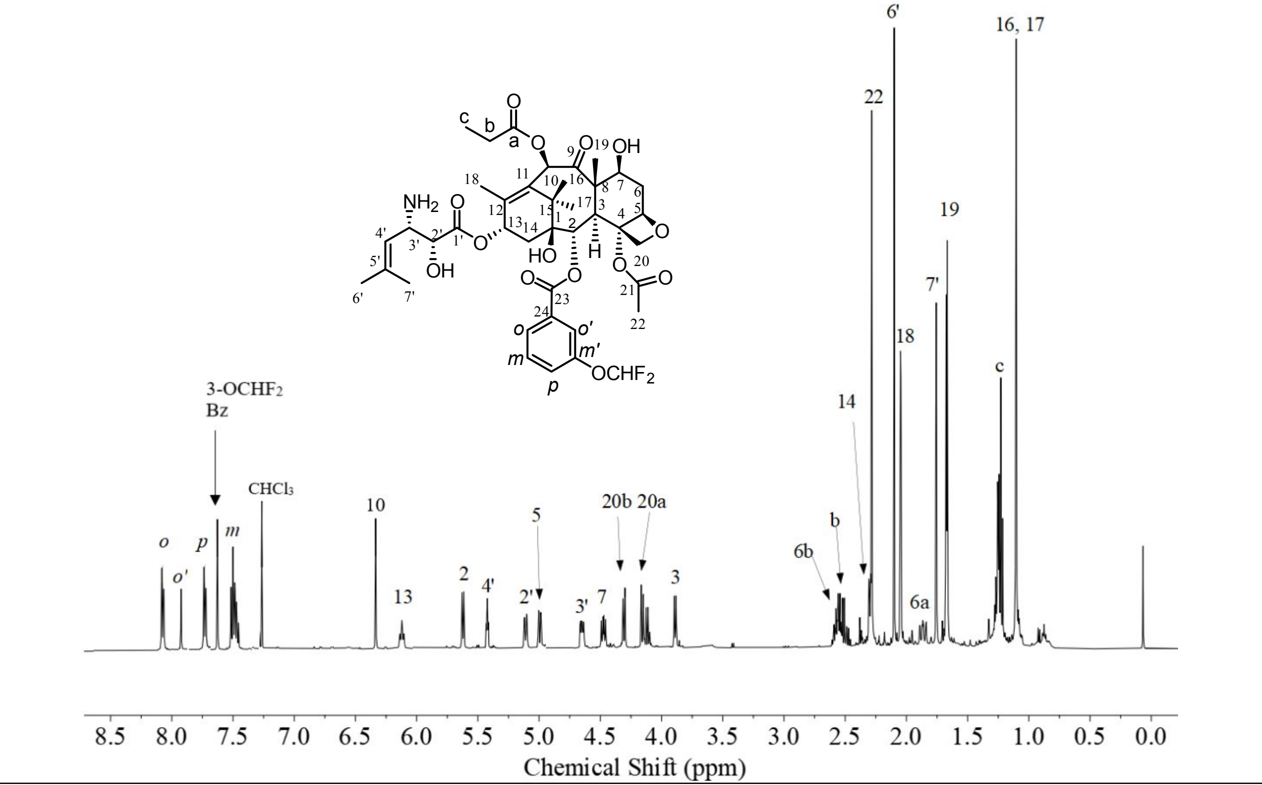

Figure S38. ^{1}H NMR of 2-debenzoyl-2-(3-$OCHF_2$-benzoyl)-3'-*N*-de(*tert*-butoxycarbonyl)-SB-T-1213.

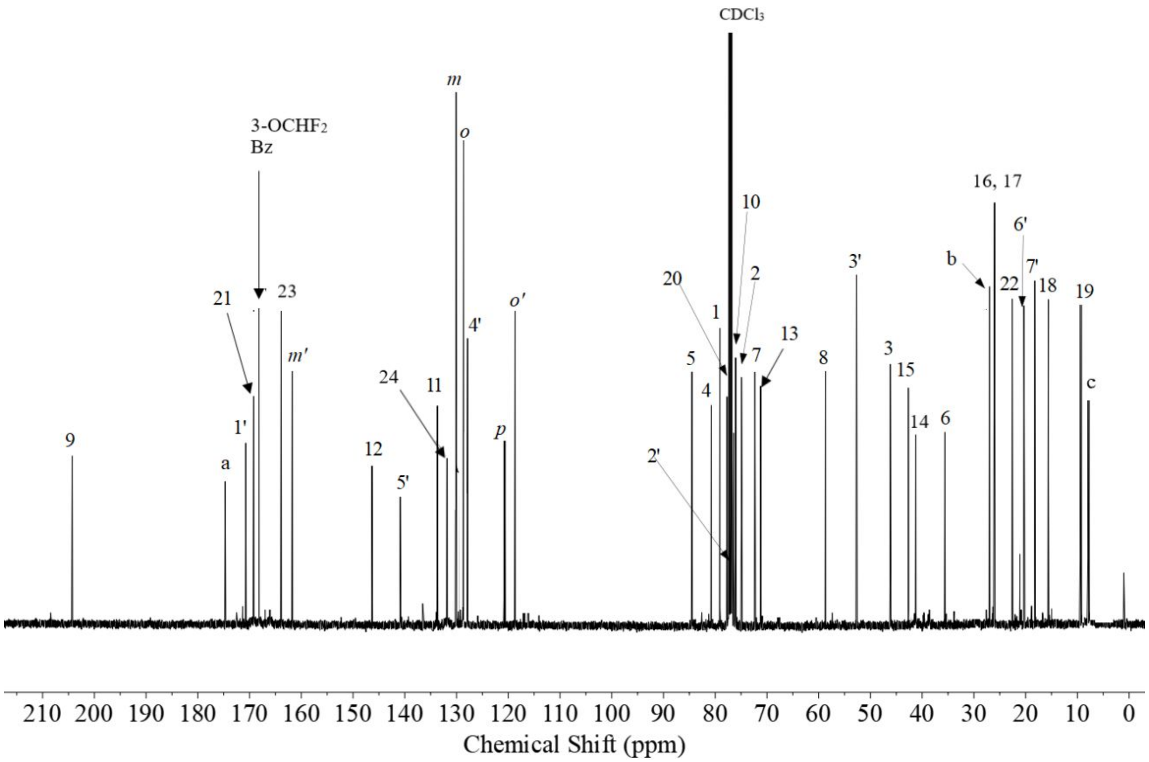

Figure S39. ^{13}C NMR of 2-debenzoyl-2-(3-OCHF$_2$-benzoyl)-3'-*N*-de(*tert*-butoxycarbonyl)-SB-T-1213.

NMR Data for 2-debenzoyl-2-(3-trifluoromethylbenzoyl)-3'-*N*-de(*tert*-butoxycarbonyl)-SB-T-1213 (10f). ^{1}H NMR (500 MHz, CDCl$_3$) δ: 8.09 (d, *J* = 7.5 Hz, 1H), 7.87 (s, 1H) 7.68 (d, *J* = 7.2 Hz, 1H), 7.45 (dd, *J* = 8.2, 7.8 Hz, 1H), 6.32 (s, 1H), 6.19 (t, *J* = 8.5 Hz, 1H), 5.63 (d, *J* = 7.1 Hz, 1H), 5.42 (d, *J* = 4.5 Hz, 1H), 5.13 (d, *J* = 4.6 Hz, 1H), 4.98 (d, *J* = 9.7 Hz, 1H), 4.64 (dd, *J* = 4.7, 4.5 Hz, 1H), 4.47 (*J* = 9, 9Hz, 1H), 4.30 (d, *J* = 9 Hz, 1H), 4.14 (d, *J* = 9 Hz, 1H), 3.89 (d, *J* = 7.1 Hz, 1H), 2.56 (m, 1H), 2.54 (q, *J* = 8.2 Hz, 2H), 2.31 (q, *J* = 7.6 Hz, 2H), 2.29 (s, 3H), 2.11 (s, 3H), 2.05 (s, 3H), 1.88 (m, 1H), 1.78 (s, 3H), 1.68 (s, 3H), 1.25 (t, *J* = 7.6 Hz, 3H), 1.11 (s, 6H) (Figure S40). ^{13}C NMR (126 MHz, CDCl3) δ: 204.27, 171.31, 170.82, 169.12, 167.35, 165.11, 146.62, 141.11, 132.18, 130.47, 129.12, 127.75, 127.72, 126.46, 122.68, 118.55, 84.49, 80.77, 79.10, 77.73, 77.32, 76.05, 74.92, 72.34, 67.91, 58.67, 52.69, 46.17, 42.68, 38.57, 35.59, 26.96, 26.01, 22.60, 20.34, 18.24, 15.61, 8.46, 8.35 (Figure S41). LC/ESI-MS monoisotopic exact mass *m/z* 826.3195 [M + H]$^{+}$; calculated for $C_{40}H_{51}F_3NO_{14}$: 826.3262.

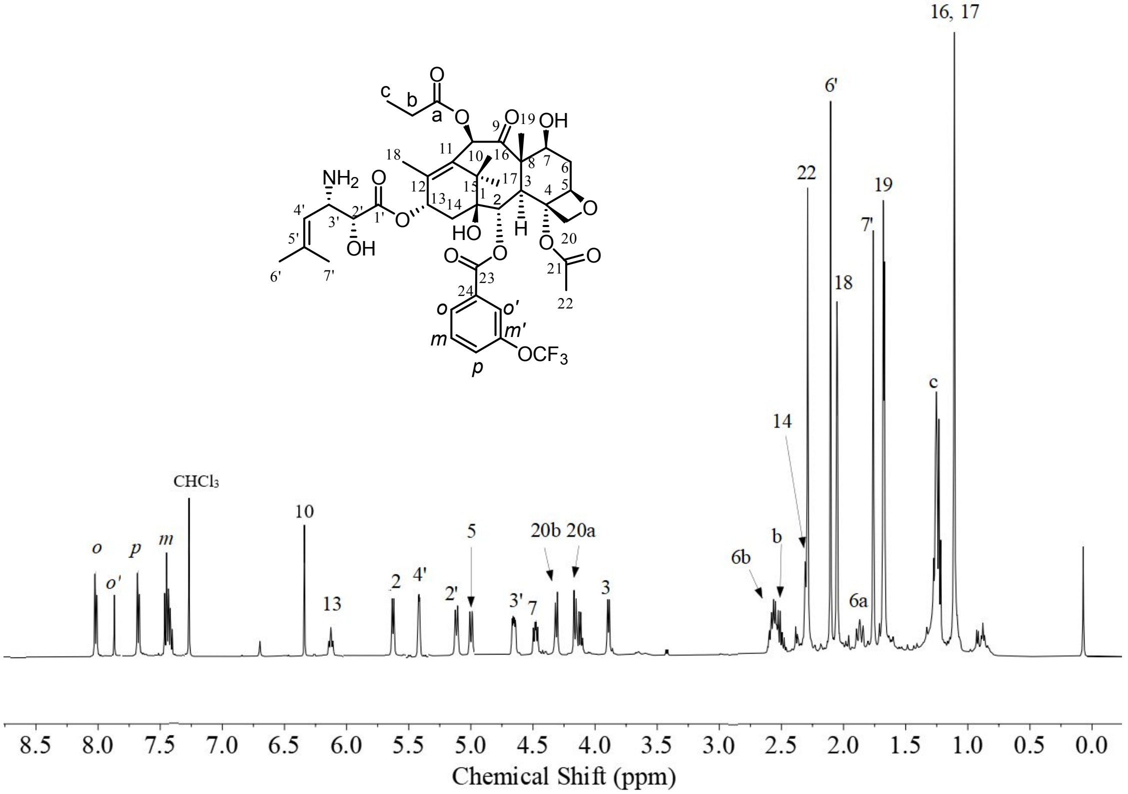

Figure S40. ^{1}H NMR of 2-Bz-2-(3-OCF_3-benzoyl)-3'-*N*-de(*tert*-butoxycarbonyl)-SB-T-1213.

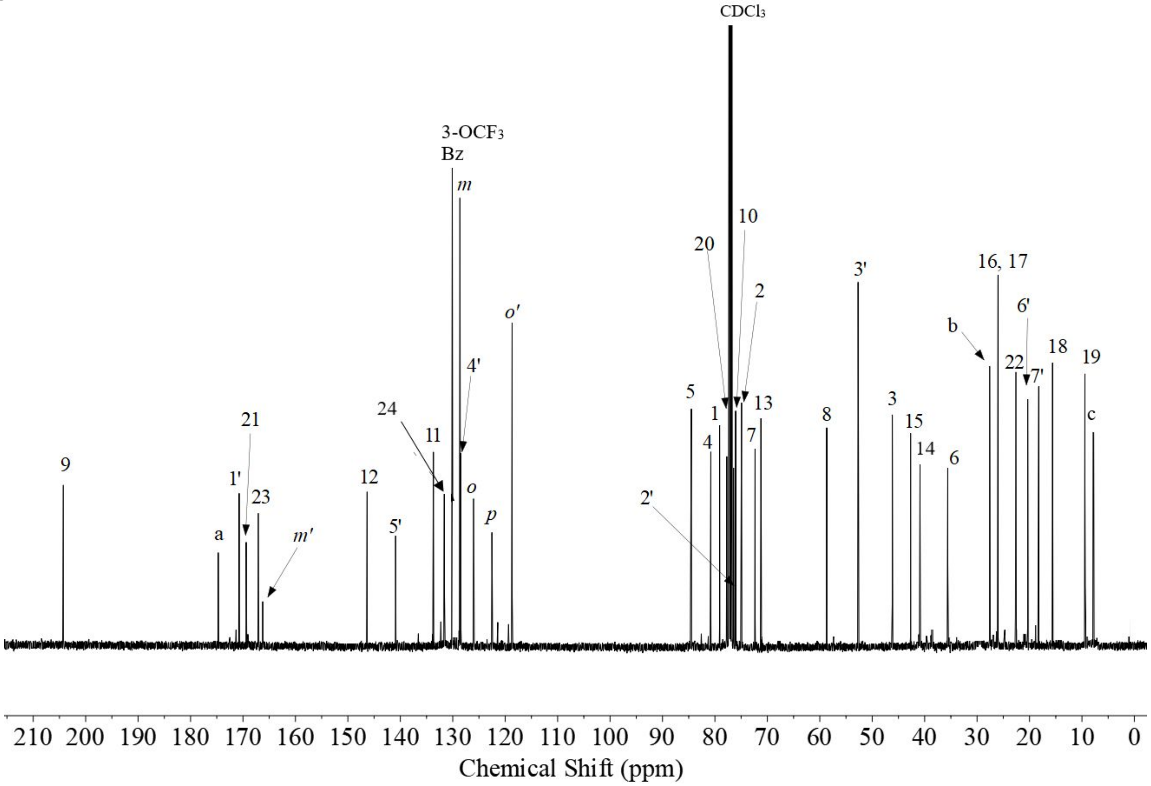

Figure S41. ^{13}C NMR of 2-debenzoyl-2-(3-OCF_3-benzoyl)-3'-*N*-de(*tert*-butoxycarbonyl)-SB-T-1213.

NMR Data for 3′-*N*-de(*tert*-butoxycarbonyl)-SB-T-1214 (11a). ^{1}H NMR (500 MHz, $CDCl_3$) δ: 8.11 (d, *J* = 8.4 Hz, 2H), 7.67 (dd, *J* = 7.4, 7.3 Hz, 1H), 7.46 (dd, *J* = 8.4, 7.5 Hz, 2H), 6.31 (s, 1H), 6.19 (t, *J* = 8.7 Hz, 1H), 5.61 (d, *J* = 7.1 Hz, 1H), 5.42 (d, *J* = 4.5 Hz, 1H), 5.11 (d, *J* = 4.7, 1H), 4.98 (d, *J* = 9.7, 1H), 4.62 (dd, *J* = 4.7, 4.5 Hz, 1H), 4.46 (dd , *J* = 9, 9 Hz, 1H), 4.30 (d, *J* = 9 Hz, 1H), 4.15 (d, *J* = 9 Hz, 1H), 3.85 (d, *J* = 7.1 Hz, 1H), 2.55 (m, 1H), 2.29 (q, *J* = 7.6 Hz, 2H), 2.26 (s, 3H), 2.07 (s, 3H), 2.03 (s, 3H), 1.84 (m, 1H), 1.75 (s, 3H), 1.66 (s, 3H), 1.24 (tt, *J* = 7.6, 4.6 Hz, 1H), 1.13 (dd, *J* = 5.3, 3.3 Hz, 2H), 1.11 (s, 6H), 1.01 (dd, *J* = 6.9, 4.6 Hz, 2H) (Figure S42). ^{13}C NMR (126 MHz, CDCl3) δ: 204.35, 175.17, 171.62, 168.83, 167.31, 148.13, 143.89, 135.43, 133.27, 131.52, 130.17, 129.52, 119.32, 84.39, 81.73, 79.82, 78.56, 77.31, 76.86, 74.27, 72.51, 67.72, 58.76, 53.12, 46.65, 42.46, 39.28, 35.11, 27.24, 22.62, 20.43, 18.62, 13.27, 8.41, 8.32 (Figure S43). LC/ESI-MS monoisotopic exact mass *m/z* 754.3383 [M + H] $^{+}$; calculated for $C_{40}H_{52}NO_{13}$: 754.3438.

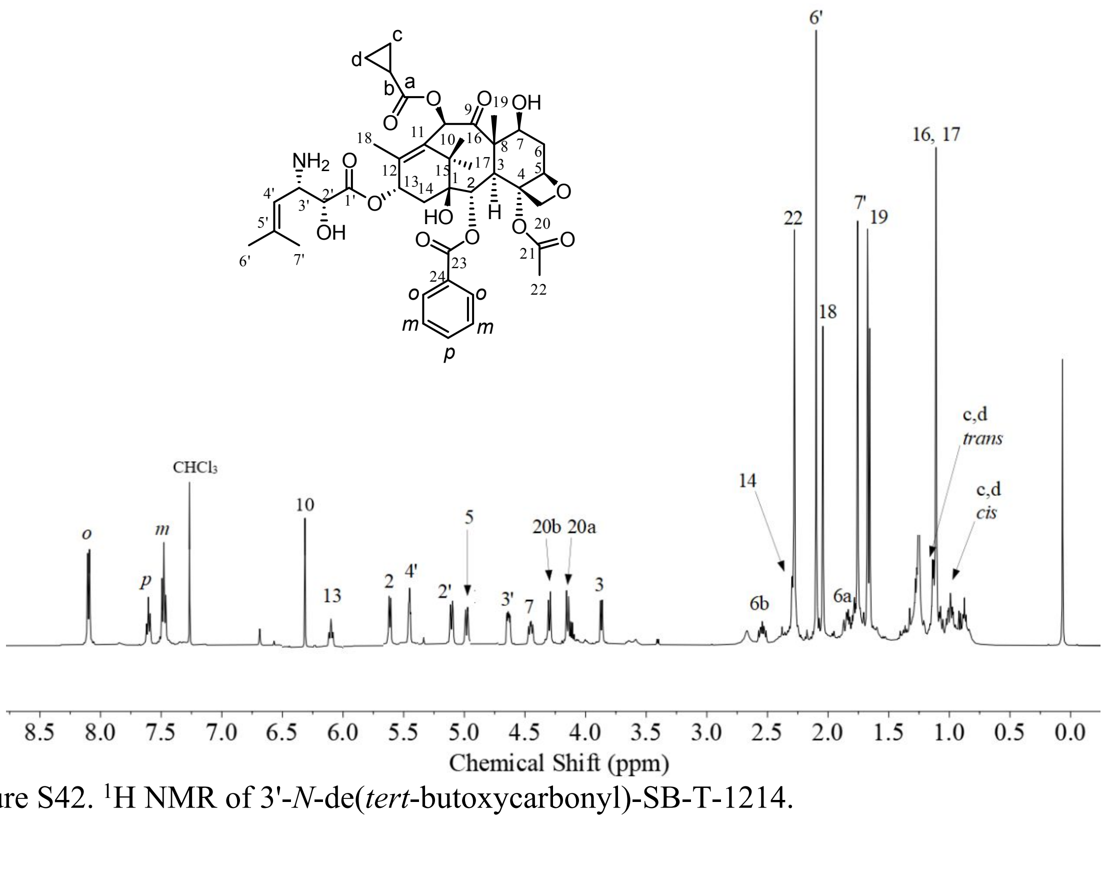

Figure S42. ^{1}H NMR of 3'-*N*-de(*tert*-butoxycarbonyl)-SB-T-1214.

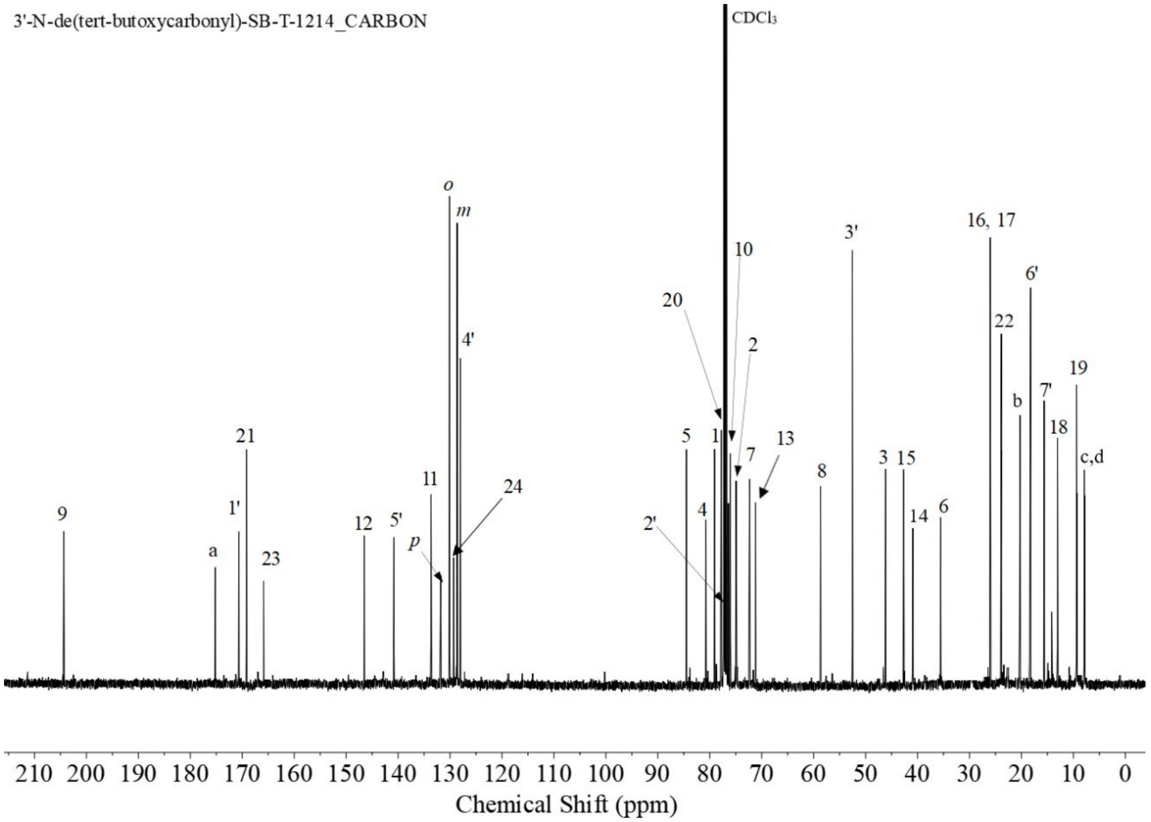

Figure S43. ^{13}C NMR of 3'-*N*-de(*tert*-butoxycarbonyl)-SB-T-1214.

NMR Data for 2-debenzoyl-2-(3-fluorobenzoyl)-3'-*N*-de(*tert*-butoxycarbonyl)-SB-T-1214 (11b). ^{1}H NMR (500 MHz, $CDCl_3$) δ: : 8.09 (d, *J* = 7.4 Hz, 1H), 7.86 (s, 1H) 7.76 (d, *J* = 7.0 Hz, 1H), 7.49 (dd, *J* = 8.4, 7.5 Hz, 1H), 6.32 (s, 1H), 6.19 (t, *J* = 8.5 Hz, 1H), 5.62 (d, *J* = 7.1 Hz, 1H), 5.42 (d, *J* = 4.5 Hz, 1H), 5.13 (d, *J* = 4.6 Hz, 1H), 4.98 (d, *J* = 9.7 Hz, 1H), 4.64 (dd, *J* = 4.7, 4.5 Hz, 1H), 4.47 (*J* = 9, 9Hz, 1H), 4.30 (d, *J* = 9 Hz, 1H), 4.14 (d, *J* = 9 Hz, 1H), 3.89 (d, *J* = 7.1 Hz, 1H), 2.55 (m, 1H), 2.31 (q, *J* = 7.6 Hz, 2H), 2.28 (s, 3H), 2.10 (s, 3H), 2.05 (s, 3H), 1.85 (m, 1H), 1.77 (s, 3H), 1.68 (s, 3H), 1.25 (tt, *J* = 7.6, 4.6 Hz, 1H), 1.14 (dd, *J* = 5.3, 3.3 Hz, 2H), 1.11 (s, 6H), 0.99 (dd, *J* = 6.9, 4.6 Hz, 2H) (Figure S44). ^{13}C NMR (126 MHz, CDCl3) δ: 205.26, 176.71, 171,68, 168.86, 167.25, 163.52, 147.56, 142.92, 134.88, 130.46, 129.96, 128.64, 125.85, 121.78, 116.67, 84.51, 81.79, 79.81, 77.37, 76.31, 74.93, 72.34, 67.92, 58.67, 53.79, 46.67, 42.76, 39.28, 35.93, 27.21, 26.92, 22.88, 20.76, 18.65, 13.61, 8.47, 8.36 (Figure S45). LC/ESI-MS monoisotopic exact mass *m/z* 772.3294 $[M + H]^+$; calculated for $C_{40}H_{51}FNO_{13}$: 772.3345.

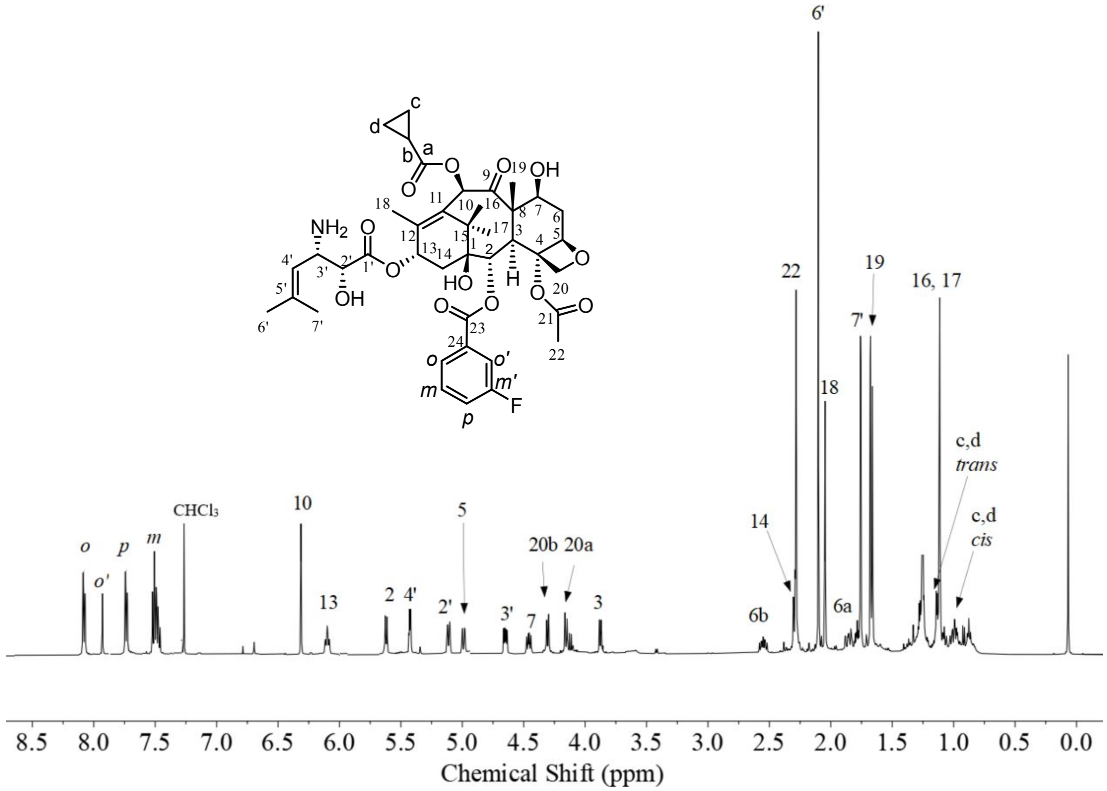

Figure S44. ^{1}H NMR of 2-debenzoyl-2-(3-F-benzoyl)-3'-*N*-de(*tert*-butoxycarbonyl)-SB-T-1214.

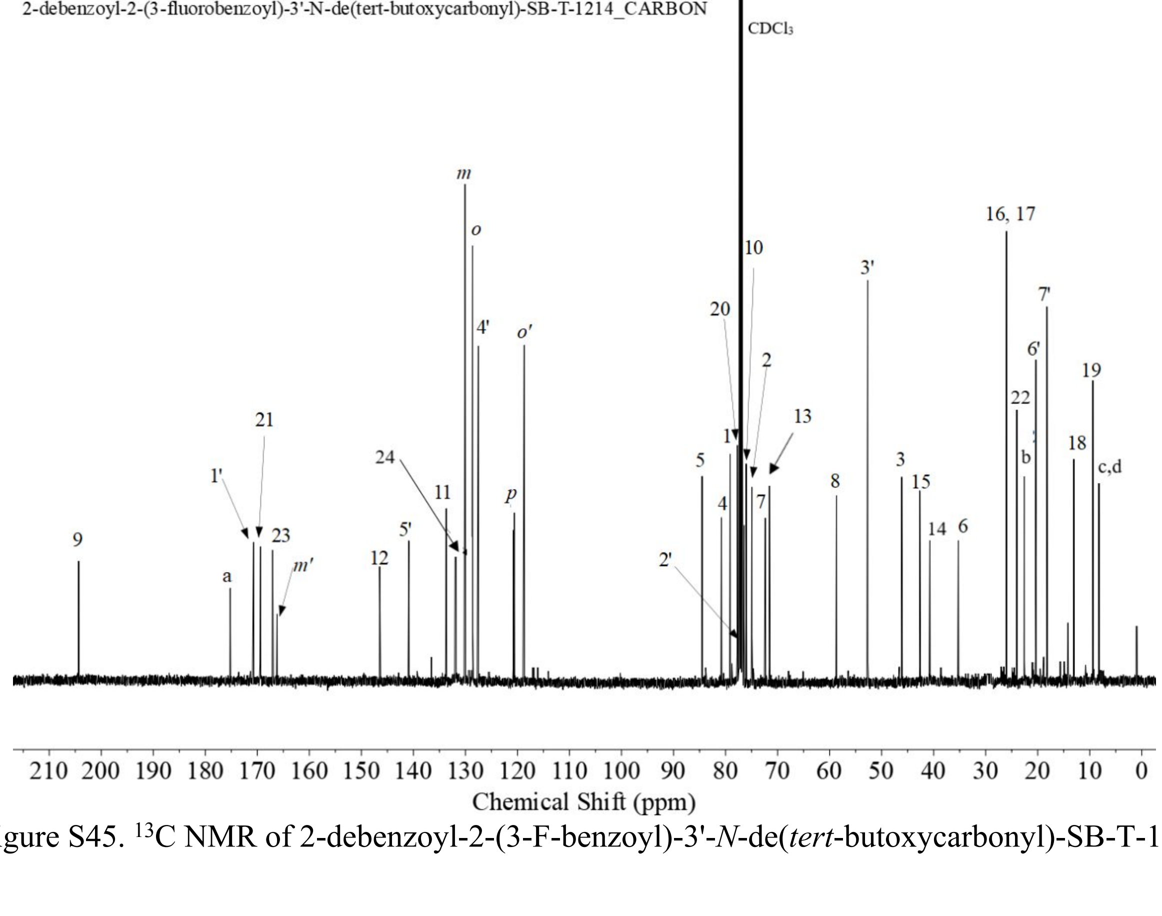

Figure S45. ^{13}C NMR of 2-debenzoyl-2-(3-F-benzoyl)-3'-*N*-de(*tert*-butoxycarbonyl)-SB-T-1214.

NMR Data for 2-debenzoyl-2-(3-chlorobenzoyl)-3'-*N*-de(*tert*-butoxycarbonyl)-SB-T-1214 (11c). ^{1}H NMR (500 MHz, $CDCl_3$) δ: 8.12 (s, 1H), 7.89 (d, *J* = 7.4 Hz, 1H), 7.77 (d, *J* = 7.2 Hz, 1H), 7.49 (dd, *J* = 8.5, 7.4 Hz, 1H), 6.32 (s, 1H), 6.19 (t, *J* = 8.5 Hz, 1H), 5.62 (d, *J* = 7.1 Hz, 1H), 5.42 (d, J = 4.5 Hz, 1H), 5.13 (d, J = 4.6 Hz, 1H), 4.98 (d, *J* = 9.7 Hz, 1H), 4.64 (dd, *J* = 4.7, 4.5 Hz, 1H), 4.47 (*J* = 9, 9Hz, 1H), 4.30 (d, *J* = 9 Hz, 1H), 4.14 (d, *J* = 9 Hz, 1H), 3.89 (d, *J* = 7.1 Hz, 1H), 2.55 (m, 1H), 2.31 (q, *J* = 7.6 Hz, 2H), 2.28 (s, 3H), 2.10 (s, 3H), 2.05 (s, 3H), 1.85 (m, 1H), 1.77 (s, 3H), 1.68 (s, 3H), 1.25 (tt, *J* = 7.6, 4.6 Hz, 1H), 1.14 (dd, *J* = 5.3, 3.3 Hz, 2H), 1.11 (s, 6H), 0.99 (dd, *J* = 6.9, 4.6 Hz, 2H) (Figure S46). ^{13}C NMR (126 MHz, $CDCl_3$) δ: 205.26, 174.71, 171,98, 168.91, 167.25, 146.43, 140.91, 135.12, 134.28, 131.62, 130.28, 129.82, 129.76, 127.65, 126.84, 84.51, 81.79, 79.81, 77.37, 76.31, 74.93, 72.34, 67.92, 58.67, 53.79, 46.67, 42.76, 39.28, 35.93, 27.21, 26.92, 22.88, 20.76, 18.65, 13.61, 8.45, 8.37 (Figure S47). LC/ESI-MS monoisotopic exact mass *m/z* 788.2986 $[M + H]^+$; calculated for $C_{40}H_{51}ClNO_{13}$: 788.3049.

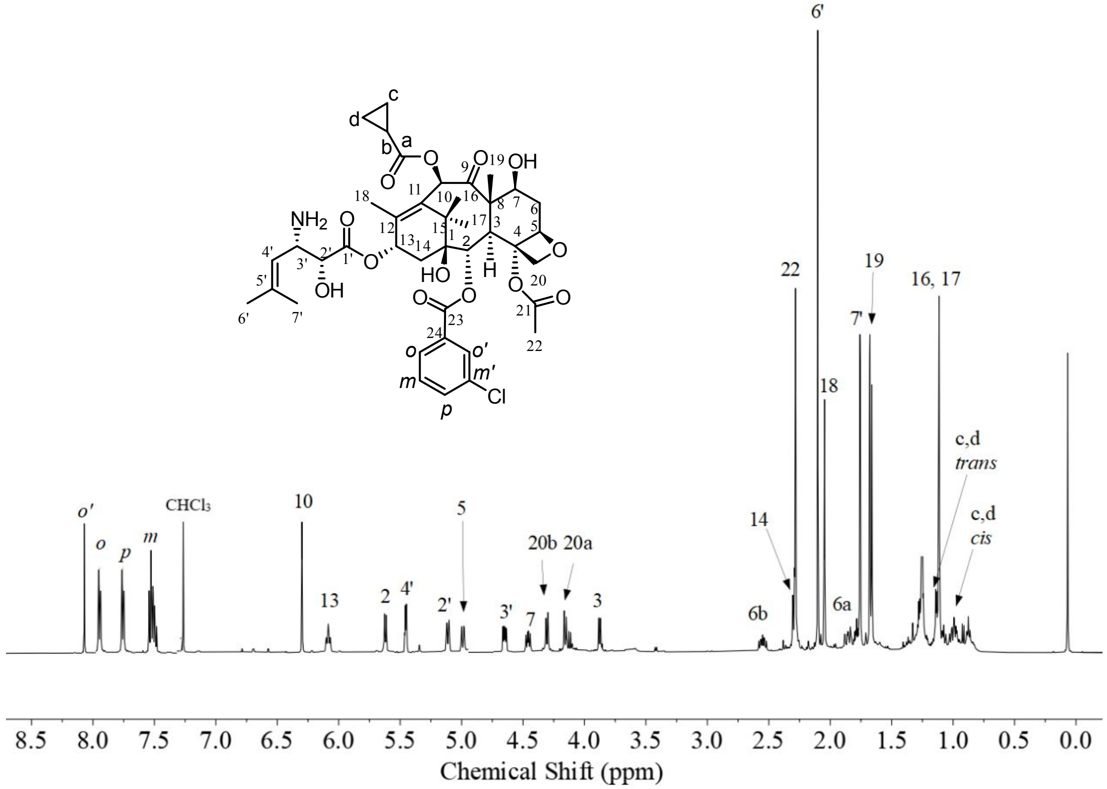

Figure S46. ^{1}H NMR of 2-debenzoyl-2-(3-Cl-benzoyl)-3'-*N*-de(*tert*-butoxycarbonyl)-SB-T-1214.

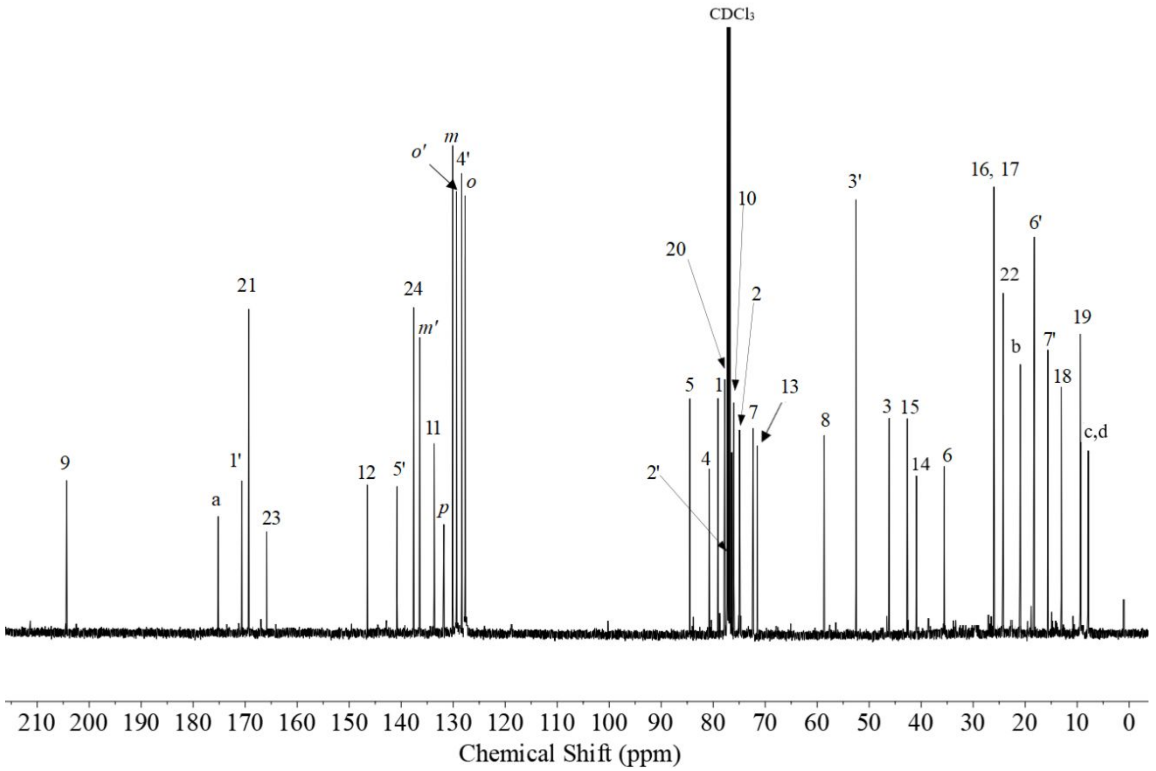

Figure S47. ^{13}C NMR of 2-debenzoyl-2-(3-F-benzoyl)-3'-*N*-de(*tert*-butoxycarbonyl)-SB-T-1214.

2-Debenzoyl-2-(3-OCH$_3$-benzoyl)-3'-*N*-de(*tert*-butoxycarbonyl)-SB-T-1214 (11d). ^{1}H NMR (500 MHz, $CDCl_3$) δ: 8.07 (d, *J* = 7.4 Hz, 1H), 7.68 (s, 1H) 7.57 (d, *J* = 7.2 Hz, 1H), 7.48 (dd, *J* = 8.5, 7.8 Hz, 1H), 6.32 (s, 1H), 6.18 (t, *J* = 8.5 Hz, 1H), 5.63 (d, *J* = 7.1 Hz, 1H), 5.45 (d, *J* = 4.5 Hz, 1H), 5.12 (d, *J* = 4.6 Hz, 1H), 4.98 (d, *J* = 9.7 Hz, 1H), 4.64 (dd, *J* = 4.7, 4.5 Hz, 1H), 4.47 (*J* = 9, 9Hz, 1H), 4.30 (d, *J* = 9 Hz, 1H), 4.14 (d, *J* = 9 Hz, 1H), 3.89 (d, *J* = 7.1 Hz, 1H), 3.86 (s, 3H), 2.54 (m, 1H), 2.30 (q, *J* = 7.6 Hz, 2H), 2.27 (s, 3H), 2.10 (s, 3H), 2.04 (s, 3H), 1.84 (m, 1H), 1.75 (s, 3H), 1.66 (s, 3H), 1.24 (tt, *J* = 7.6, 4.6 Hz, 1H), 1.14 (dd, *J* = 5.3, 3.3 Hz, 2H), 1.11 (s, 6H), 0.99 (dd, *J* = 6.9, 4.6 Hz, 2H) (Figure S48). ^{13}C NMR (126 MHz, CDCl3) δ: 204.35, 175.21, 171.14, 167.59, 166.16, 159.52, 146.17, 141.46, 133.48, 130.83, 129.46, 128.35, 122.33, 118.72, 114.25, 84.28, 81.14, 79.35, 77.31, 76.75, 76.15, 74.59, 72.24, 67.78, 58.56, 55.12, 52.35, 46.15, 42.67, 38.58, 35.54, 27.15, 26.55, 22.57, 20.34, 15.63, 13.62, 8.41, 8.36(Figure S49). LC/ESI-MS monoisotopic exact mass *m/z* 784.3491 $[M + H]^+$; calculated for $C_{41}H_{54}NO_{14}$: 784.3544.

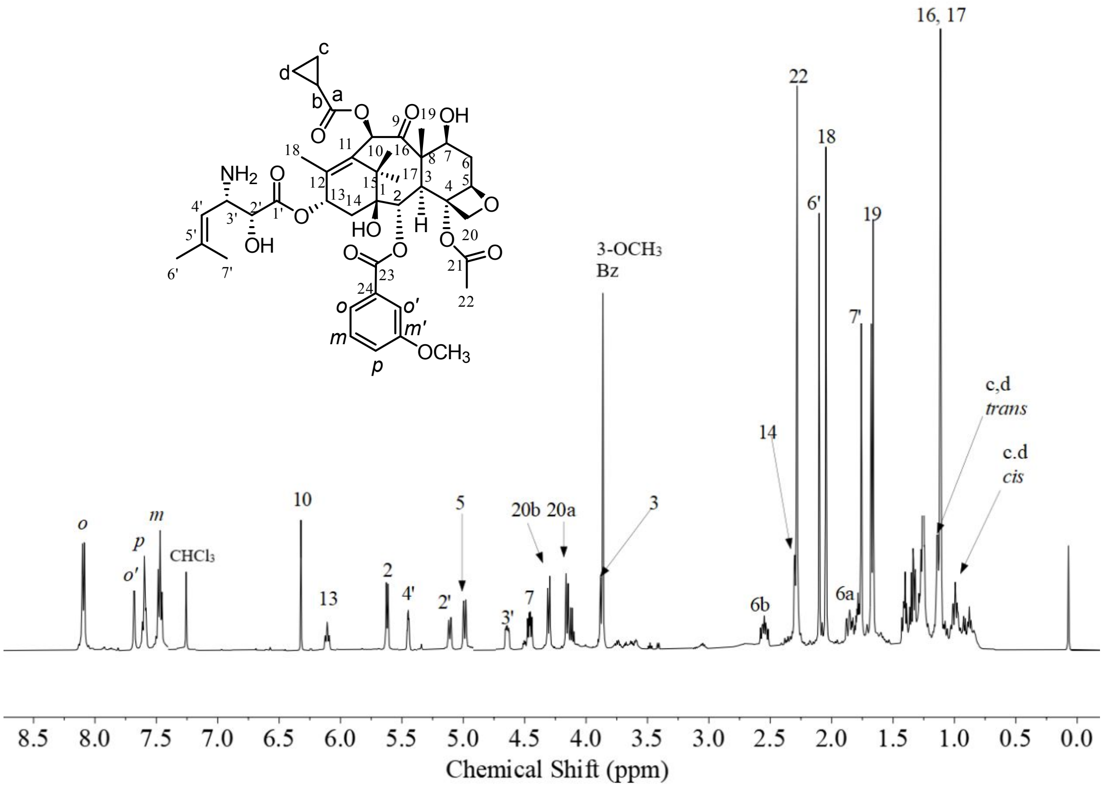

Figure S48. ^{1}H NMR of 2-debenzoyl-2-(3-OCH$_3$-benzoyl)-3'-*N*-de(*tert*-butoxycarbonyl)-SB-T-1214.

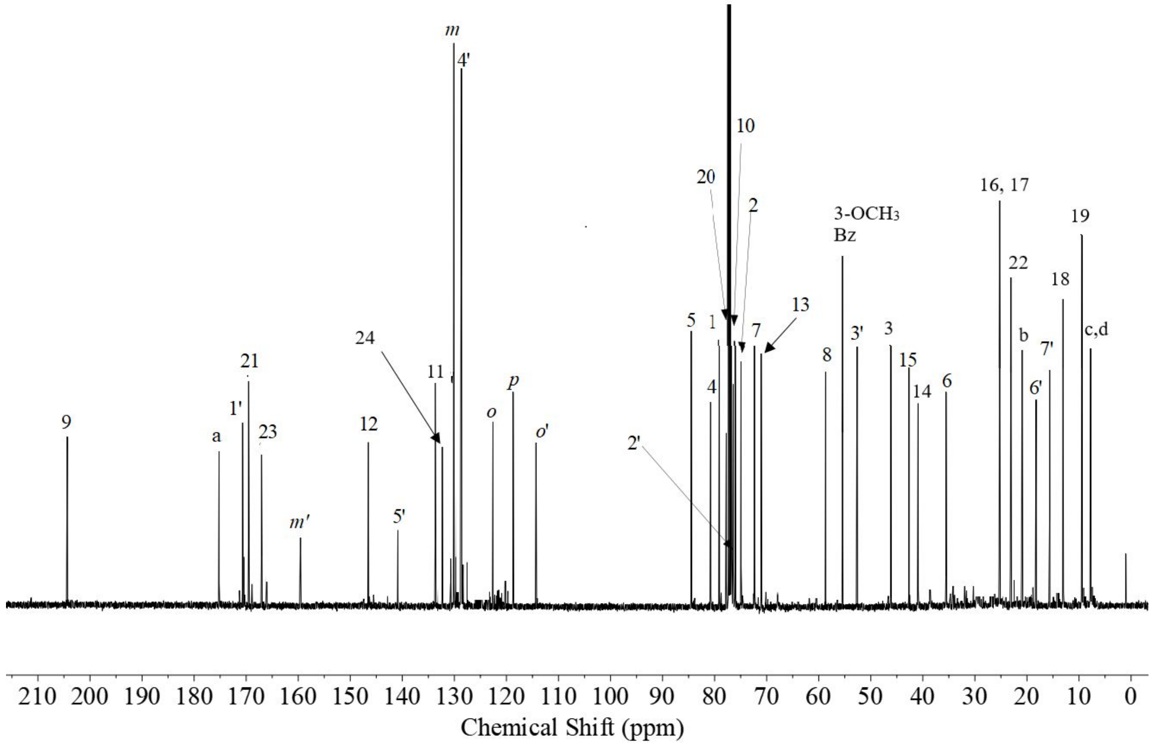

Figure S49. ^{13}CH NMR of 2-debenzoyl-2-(3-OCH$_3$-benzoyl)-3'-*N*-de(*tert*-butoxycarbonyl)-SB-T-1214.

2-Debenzoyl-2-(3-difluoromethylbenzoyl)-3'-*N*-de(*tert*-butoxycarbonyl)-SB-T-1214 (11e). ^{1}H NMR (500 MHz, CDCl3) δ: 8.11 (d, J = 7.4 Hz, 1H), 7.84 (s, 1H), 7.74 (d, J = 7.1Hz, 1H), 7.65 (s, 1H), 7.48 (dd, J = 8.4, 7.5 Hz, 1H), 6.32 (s, 1H), 6.19 (t, J = 8.5 Hz, 1H), 5.62 (d, J = 7.1 Hz, 1H), 5.42 (d, J = 4.5 Hz, 1H), 5.13 (d, J = 4.6 Hz, 1H), 4.98 (d, J = 9.7 Hz, 1H), 4.64 (dd, J = 4.7, 4.5 Hz, 1H), 4.47 (J = 9, 9Hz, 1H), 4.30 (d, J = 9 Hz, 1H), 4.14 (d, J = 9 Hz, 1H), 3.89 (d, J = 7.1 Hz, 1H), 2.55 (m, 1H), 2.29 (q, J = 7.6 Hz, 2H), 2.26 (s, 3H), 2.07 (s, 3H), 2.03 (s, 3H), 1.84 (m, 1H), 1.75 (s, 3H), 1.66 (s, 3H), 1.24 (tt, J = 7.6, 4.6 Hz, 1H), 1.13 (dd, J = 5.3, 3.3 Hz, 2H), 1.11 (s, 6H), 1.01 (dd, J = 6.9, 4.6 Hz, 2H) (Figure S50). 13C NMR (126 MHz, CDCl$_3$) δ: 206.28, 171.45, 170.65, 169.32, 167.46, 164.81, 147.35, 141.23, 132.82, 131.36, 129.52, 128.41, 127.91, 118.65, 116.72, 84.28, 81.14, 79.35, 77.31, 76.75, 76.15, 74.59, 72.24, 67.78, 58.56, 55.12, 52.35, 46.15, 42.67, 38.58, 35.54, 27.15, 26.55, 22.57, 20.34, 15.63, 13.62, 8.41, 8.36 (Figure S51). LC/ESI-MS monoisotopic exact mass m/z 820.3296 [M + H] +; calculated for C41H52F2NO14: 820.3356.

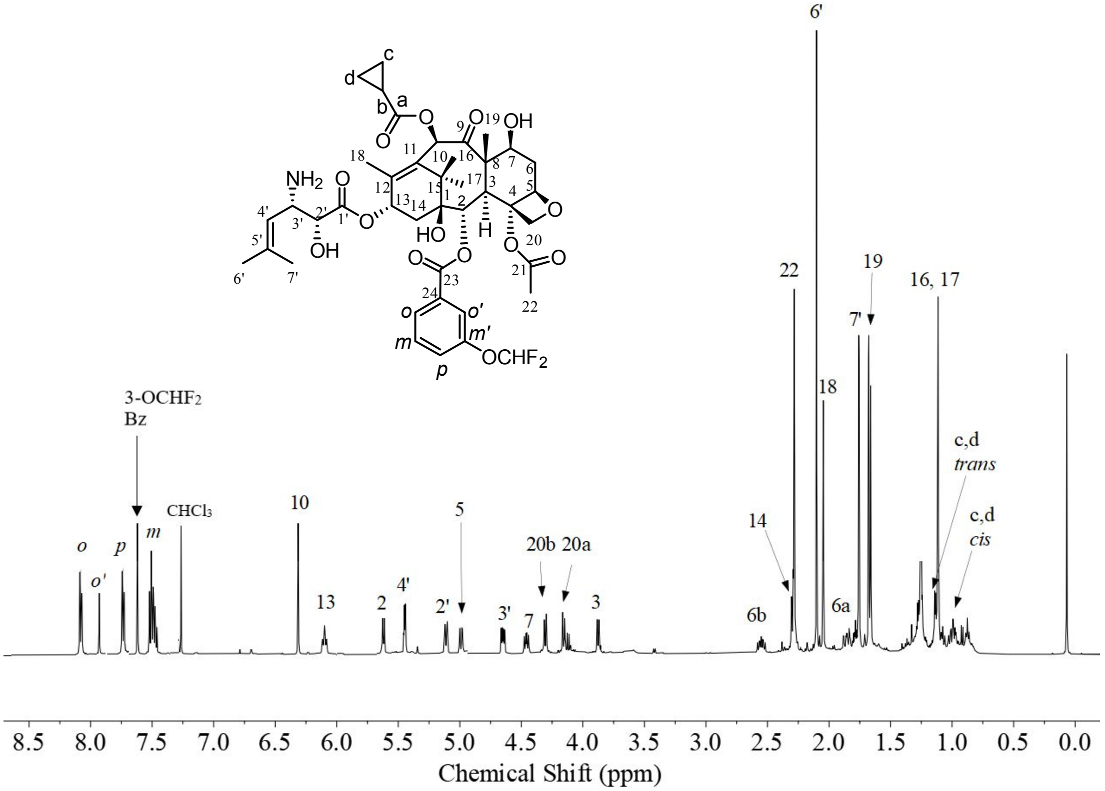

Figure S50. ^{1}H NMR of 2-debenzoyl-2-(3-OCHF$_2$-benzoyl)-3'-*N*-de(*tert*-butoxycarbonyl)-SB-T-1214.

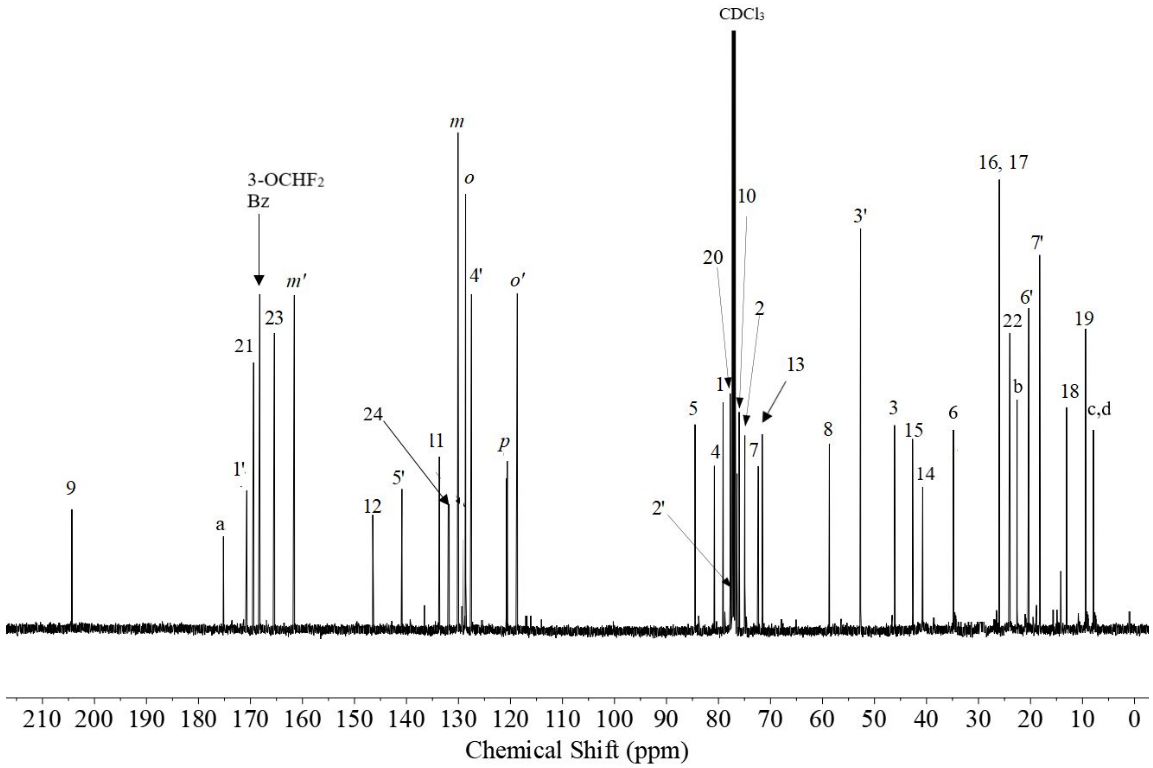

Figure S51. ^{13}C NMR of 2-debenzoyl-2-(3-$OCHF_2$-benzoyl)-3'-*N*-de(*tert*-butoxycarbonyl)-SB-T-1214.

2-Debenzoyl-2-(3-trifluoromethoxybenzoyl)-3'-*N*-de(*tert*-butoxycarbonyl)-SB-T-1214 (11f). ^1H NMR (500 MHz, $CDCl_3$) δ: 8: 8.09 (d, J = 7.5 Hz, 1H), 7.87 (s, 1H) 7.68 (d, J = 7.2 Hz, 1H), 7.45 (dd, J = 8.2, 7.8 Hz, 1H), 6.32 (s, 1H), 6.19 (t, J = 8.5 Hz, 1H), 5.63 (d, J = 7.1 Hz, 1H), 5.42 (d, J = 4.5 Hz, 1H), 5.13 (d, J = 4.6 Hz, 1H), 4.98 (d, J = 9.7 Hz, 1H), 4.64 (dd, J = 4.7, 4.5 Hz, 1H), 4.47 (J = 9, 9Hz, 1H), 4.30 (d, J = 9 Hz, 1H), 4.14 (d, J = 9 Hz, 1H), 3.89 (d, J = 7.1 Hz, 1H), 2.56 (m, 1H), 2.30 (q, J = 7.6 Hz, 2H), 2.29 (s, 3H), 2.11 (s, 3H), 2.06 (s, 3H), 1.86 (m, 1H), 1.76 (s, 3H), 1.68 (s, 3H), 1.25 (tt, J = 7.6, 4.6 Hz, 1H), 1.15 (dd, J = 5.3, 3.3 Hz, 2H), 1.12 (s, 6H), 1.00 (dd, J = 6.9, 4.6 Hz, 2H) (Figure S52). ^{13}C NMR (126 MHz, $CDCl_3$) δ: 204.36, 175.35, 171.72, 169.11, 167.65, 166.63, 146.47, 141.81, 133.68, 131.83, 130.88, 129.31, 128.46, 122.54, 118.84, 84.48, 81.72, 79.75, 77.32, 76.81, 76.28, 74.91, 72.31, 67.87, 58.61, 53.67, 46.62, 42.67, 38.75, 35.54, 27.12, 26.85, 22.58, 20.33, 18.23, 13.58, 8.41, 8.35 (Figure S53). LC/ESI-MS monoisotopic exact mass m/z 838.3184 $[M + H]^+$; calculated for $C_{41}H_{51}F_3NO_{14}$: 838.3262.

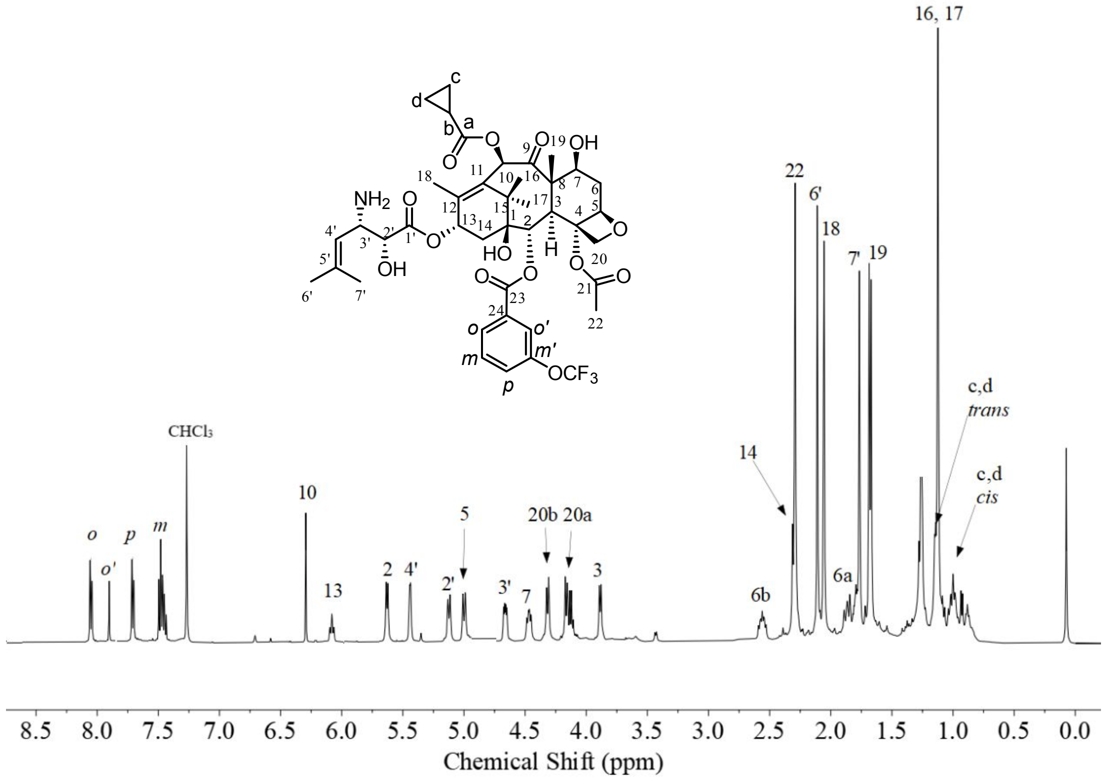

Figure S52. ^{1}H NMR of 2-debenzoyl-2-(3-OCF_3-benzoyl)-3'-*N*-de(*tert*-butoxycarbonyl)-SB-T-1214.

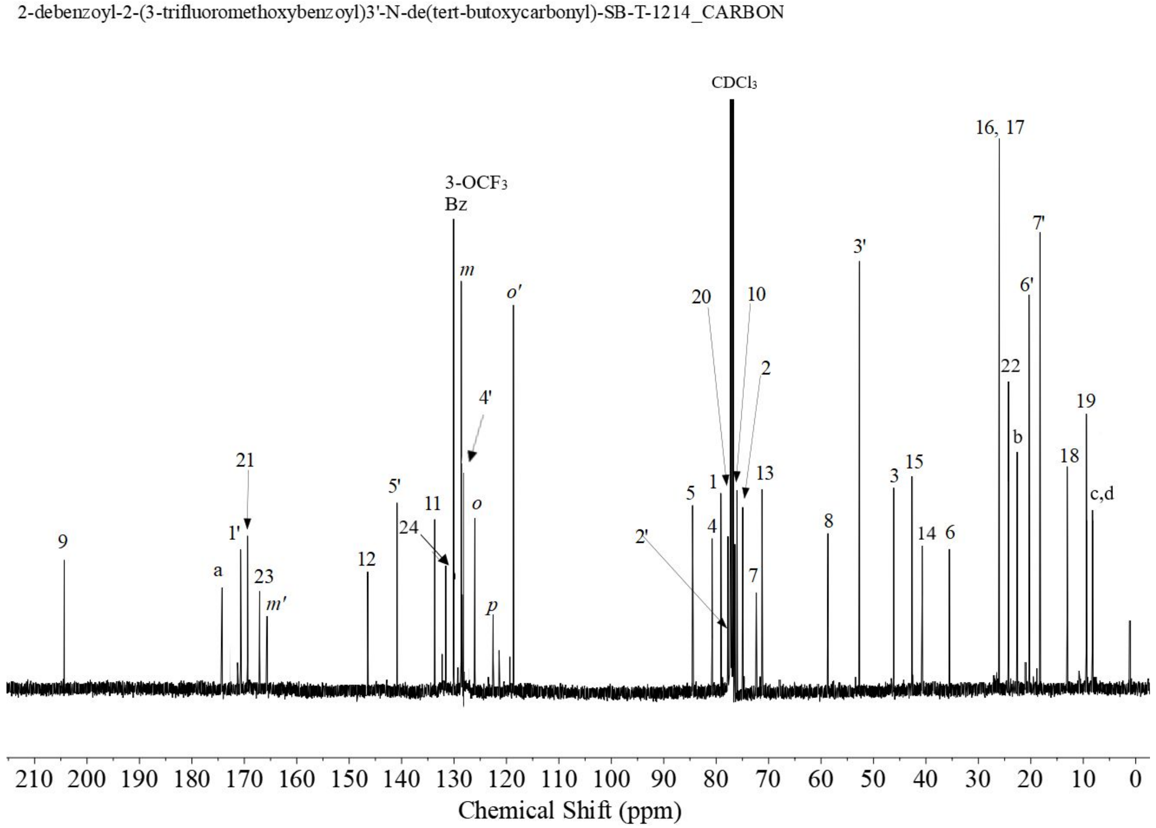

Figure S53. ^{13}C NMR of 2-debenzoyl-2-(3-OCF_3-benzoyl)-3'-*N*-de(*tert*-butoxycarbonyl)-SB-T-1214.

9a

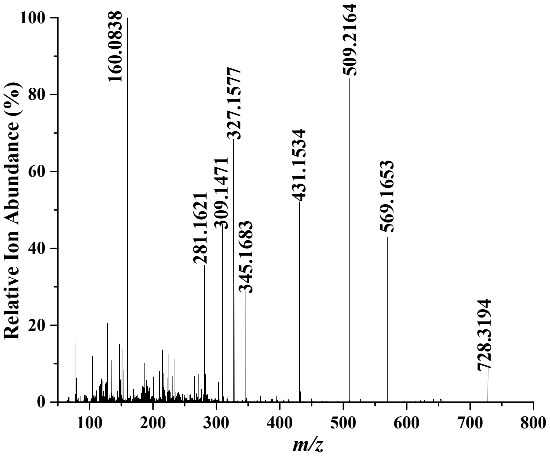

9b

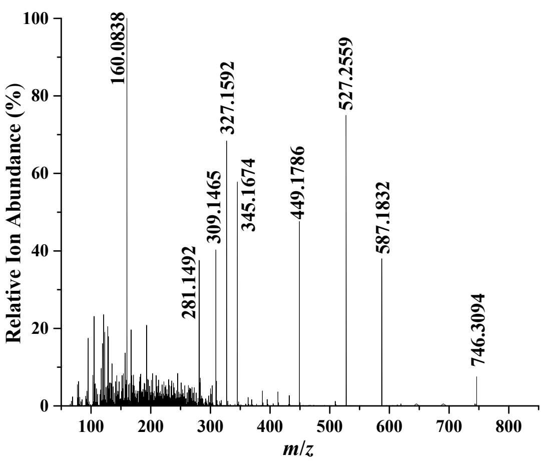

9c

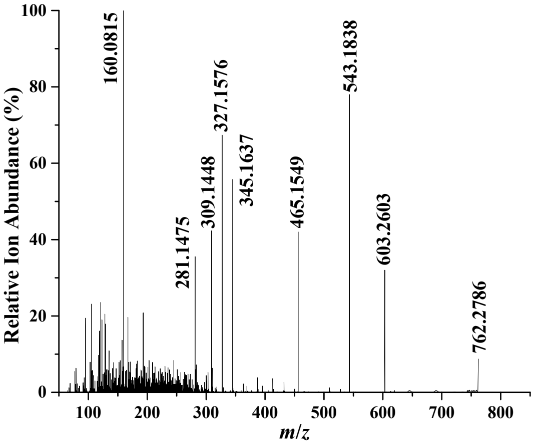

9d

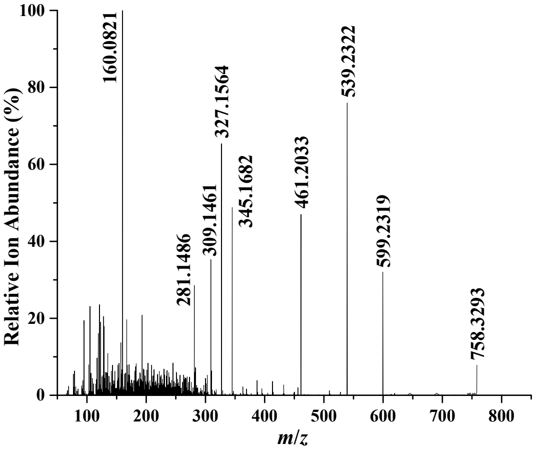

9e

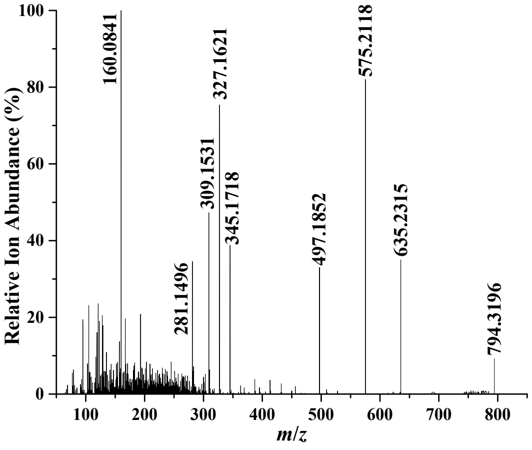

9f

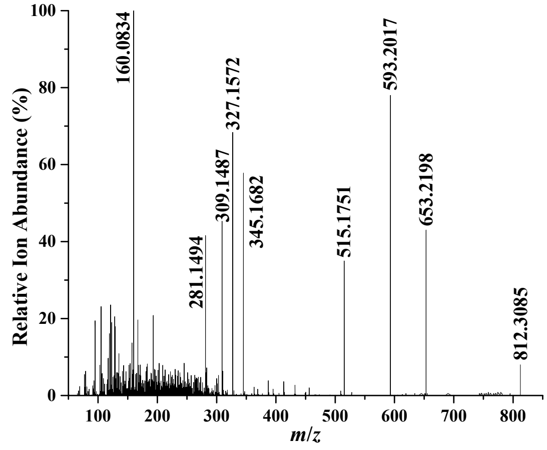

Figure S54. LC/ESI-MS/MS positive-ion mode of purified of 3'-*N*-de(*tert*-butoxycarbonyl)-SB-T-1212 analogs **9a-f**.

10a

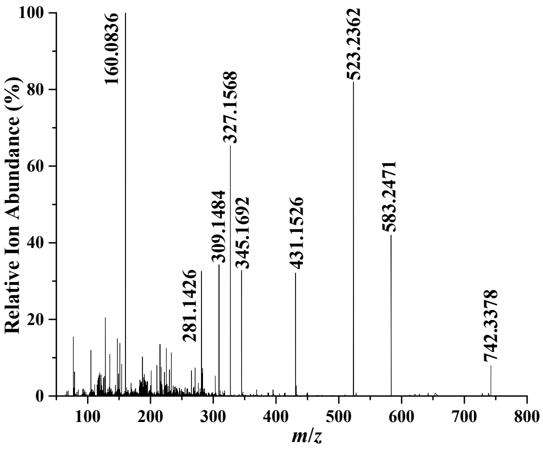

10b

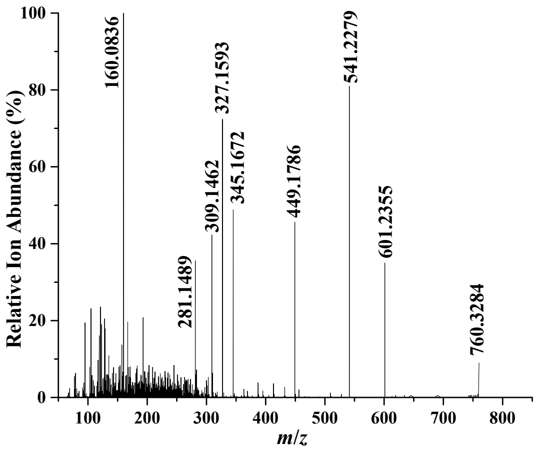

10c

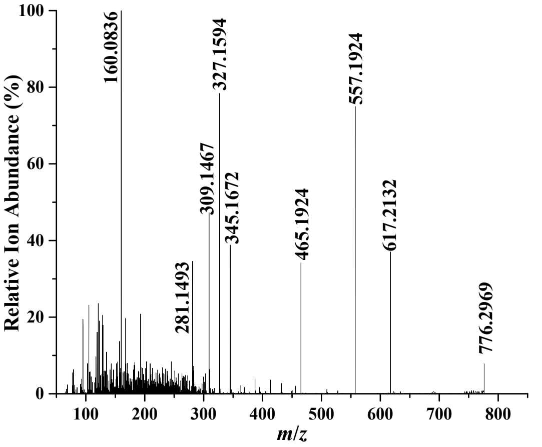

10d

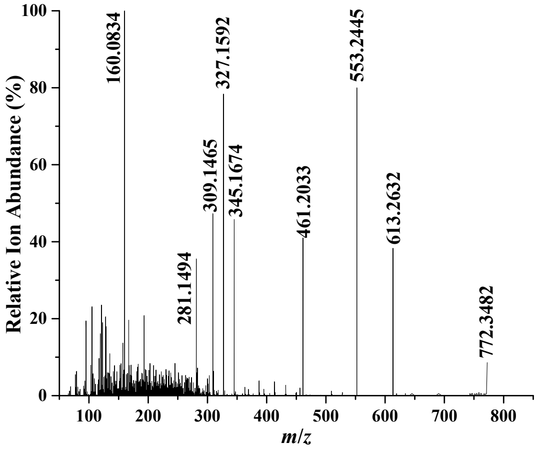

10e

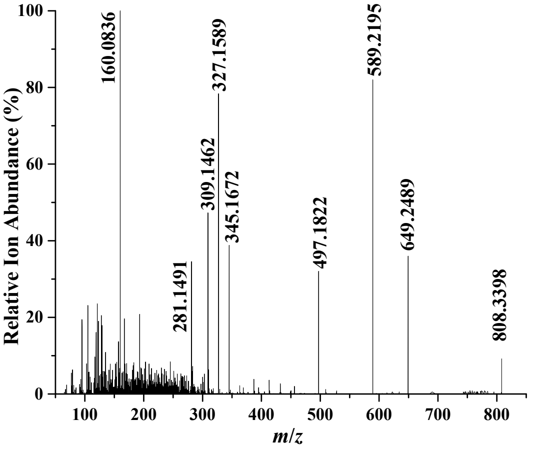

10f

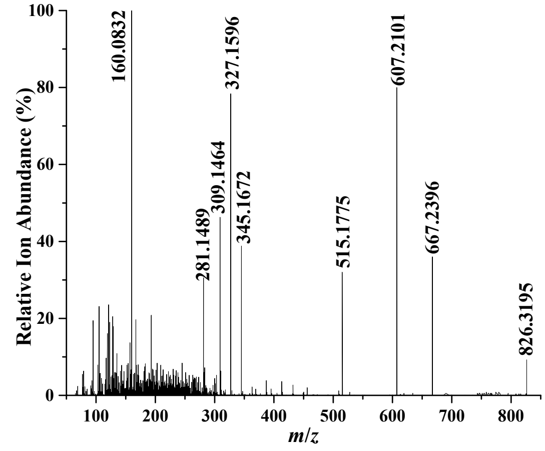

Figure S55. LC/ESI-MS/MS positive-ion mode of purified of 3'-*N*-de(*tert*-butoxycarbonyl)-SB-T-1213 analogs **10a-f**.

11a

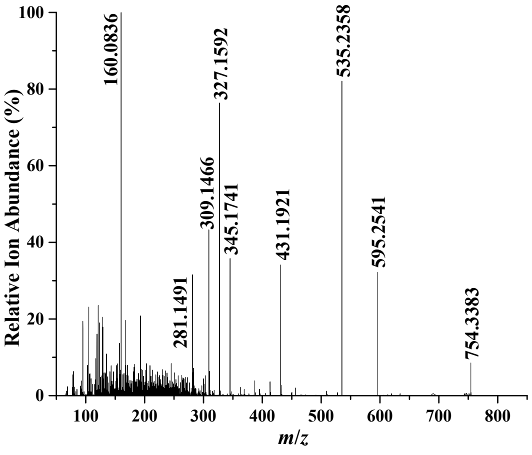

11b

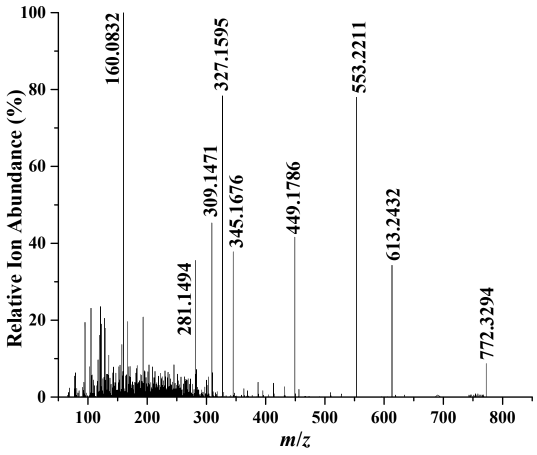

11c

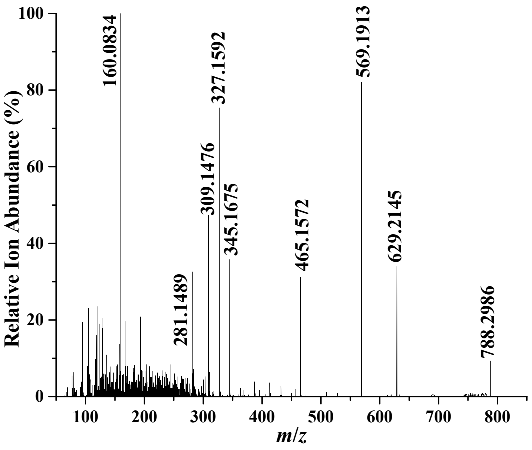

11d

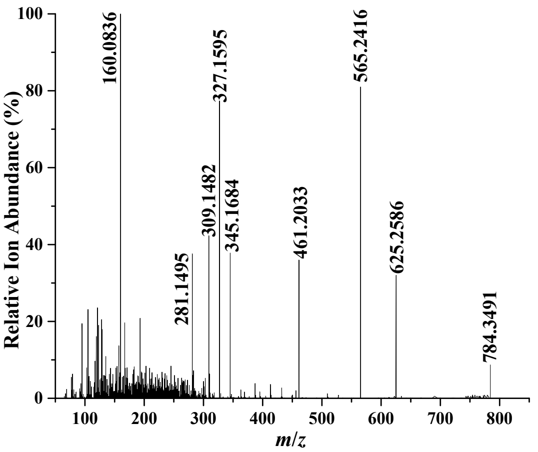

11e

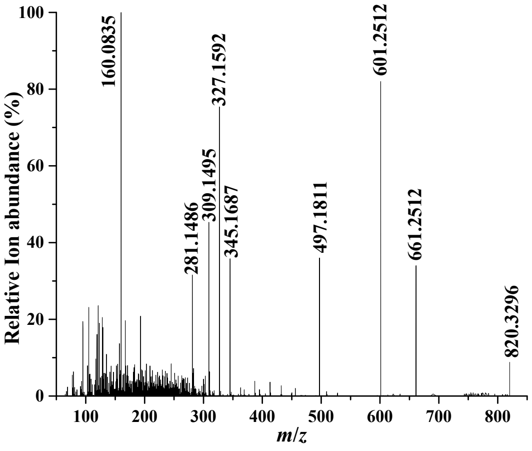

11f

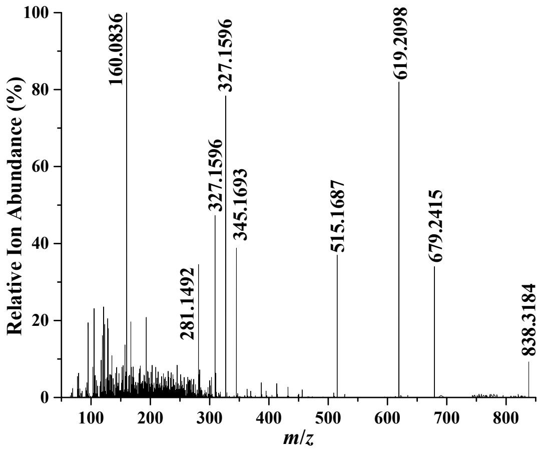

Figure S56. LC/ESI-MS/MS positive-ion mode of purified of 3'-*N*-de(*tert*-butoxycarbonyl)-SB-T-1214 analogs **11a**-**f**.
